# The Diversity of Midgut Bacteria among Wild-Caught *Phlebotomus argentipes* (Psychodidae: Phlebotominae), the Vector of Leishmaniasis in Sri Lanka

**DOI:** 10.1155/2020/5458063

**Published:** 2020-08-19

**Authors:** Nayana Gunathilaka, Hirunika Perera, Tharaka Wijerathna, Wasana Rodrigo, N. D. A. D. Wijegunawardana

**Affiliations:** ^1^Department of Parasitology, Faculty of Medicine, University of Kelaniya, Ragama, Sri Lanka; ^2^Biotechnology Unit, Industrial Technology Institute, Colombo 07, Sri Lanka; ^3^Department of Bioprocess Technology, Faculty of Technology, Rajarata University of Sri Lanka, Sri Lanka

## Abstract

*Phlebotomus argentipes* is the main suspected vector for leishmaniasis in Sri Lanka. Investigations on the presence of aerobic bacteria in the gut of sand flies which evidence a potential approach to control leishmaniasis transmission through a paratransgenic strategy are still not available for the local sand fly populations. Field-caught unfed female sand flies collected from three selected Medical Officer of Health (MOH) areas (Polpithigama, Maho, and Galgamuwa) in Kurunegala District, Sri Lanka from August to December 2018 were used. Prokaryotic 16S ribosomal RNA partial gene was amplified and sequenced. Morphological identification revealed the presence of only one sand fly species, *P. argentipes* (*n* = 1,969). A total of 20 organisms belonging to two phyla (Proteobactericea and Furmicutes) were detected within the gut microbial community of the studied sand fly specimens. This study documents the first-ever observation of *Rhizobium* sp. in the midgut of *P. argentipes*. The presence of *Bacillus megaterium*, which is considered as a nonpathogenic bacterium with potential use for paratransgenic manipulation of *P. argentipes* suggest that it may be used as a delivery vehicle to block the vectorial transmission of *Leishmania* parasites. In addition, *Serratia marcescens* may be used as a potential candidate to block the parasite development in sand fly vectors since it has evidenced antileishmanial activities in previous investigations. Hence, further studies are required to gain full insight into the potential use of this bacterium in the control of *Leishmania* parasites through paratransgenesis.

## 1. Introduction

Leishmaniasis is a vector-borne disease transmitted through female sand flies (Psychodidae: Phlebotomine), and it is caused by a unicellular protozoan parasite belonging to the genus *Leishmania*. It is considered a neglected tropical disease, and at present, this disease is endemic in 102 countries [1]. Female sand flies feed on mammalian blood for egg development and maturation. As both males and females feed on plant nectar as a sugar source, they may acquire plant bacteria [2]. During larval development, they feed on organic detritus which may contain a wide range of microorganisms. Previous studies have indicated that newly emerged sand flies were associated with a large amount of bacterial DNA that could be taken from the environment by feeding or transtadial passage [3].

In Sri Lanka, *Phlebotomus argentipes* is considered the vector for leishmaniasis transmission [4]. Several studies have recorded the prevalence of bacterial community in the midgut sand fly vectors. However, such an investigation has not been conducted in Sri Lanka. The microbial community may be different from country to country as it depends on the geographical distribution of insects [5]. Currently, there is no effective vector control programme against sand flies implemented within the country. Well-planned, integrated vector management practices that combine physical, chemical, and biological methods are essential for the successful control of leishmaniasis through the prevention of transmission.

It is important to emphasize that when considering any means of biological control, its effectiveness, ecological soundness, and sustainability should be determining factors. Most of the available biological control methods focus on killing the insect vector, while other methods such as the sterile insect technique (SIT), release of insects carrying a dominant lethal (RIDL) [6], and paratransgenesis [7] are also being tested to suppress the vector population. Of these, the use of paratransgenic sand flies has emerged recently as a promising option for the control of leishmaniasis transmission [7].

Female sand flies may ingest macrophages infected with amastigotes during feeding on blood meal from an infected vertebrate host. Later in the midgut of sand flies, the parasitized cells digest and release amastigotes. The conditions in the midgut stimulate the transformation of amastigotes into flagellated promastigote form. Thus, possible bacteria-parasite interactions take place between the gut microbial community and parasite [8, 9]. In paratransgenic strategies to control insect vectors, symbiotic gut-associated bacteria of insects are transformed to express molecules with antiparasitic activity [10, 11]. The introduction of these transformed organisms will result in antiparasitic activity in the gut of sand flies by which means the pathogen's transmission is prevented.

Several studies have reported the presence of aerobic bacteria in the gut of sand flies [12, 13], while a more recent study has claimed that this association between sand flies and microbiota may depend on the environment in which they live and may not demonstrate specificity for gut colonization to a particular host fly species [7]. Therefore, a first-hand understanding of which species of gut bacteria are present in the local sand fly population in Sri Lanka would be useful to select a candidate species to be used for transformation experiments in the future. Hence, the proposed study is aimed at screening the availability of such gut microbiome that may have an antiparasitic property or easy transformable species in order to evaluate the effectiveness of a paratransgenic strategy as a means to control leishmaniasis in Sri Lanka.

## 2. Method

### 2.1. Collection of Sand Flies

Sand flies were collected from three selected Medical Officer of Health (MOH) areas (Polpithigama, Maho, and Galgamuwa) in Kurunegala District (228-333°N, 104-178°E), North Western Province of Sri Lanka which is a well-known endemic focus for cutaneous leishmaniasis. It covers a land area of 4,816 km^2^ in the country with 1,610,299 inhabitants. The district receives an average of 2,095 mm of rainfall annually. The average temperature and humidity are 31.7°C and 69.6%, respectively. The major activities of the population are agriculture and animal farming.

**Figure 1:**
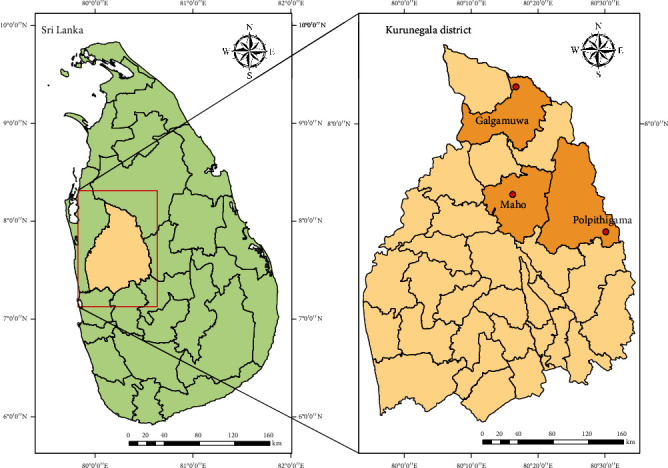
The area map indicating the surveillance sites.

Cattle-baited net traps (CBNT) were used to collect sand flies. The trap was set at 7.00 p.m. at each location during August to December 2018 and searched for adult sand flies from 9.00 to 10.00 p.m. and from 4.00 to 5.00 a.m. of the following day. The sampling locations of sand flies during the field surveys are illustrated in Figure 1.

### 2.2. Processing and Identification of Field-Caught Sand Flies

The live field-caught sand flies were transferred to the laboratory at the Department of Parasitology, Faculty of Medicine, University of Kelaniya, Ragama, Sri Lanka. Sand flies were first immobilized on ice. Each sand fly was sterilized from 60 seconds in 30 *μ*L of 70% ethanol and rinsed thoroughly using phosphate-buffered saline (PBS) (50 *μ*L of 1x sterile PBS (pH 7.3)) prior to dissection. This step was performed to confirm that there was no bacterial contamination from the surface. The final wash of this cleaning procedure was used for subsequent dissection analysis.

### 2.3. Dissection of the Midgut of Sterilized Sand Flies

The sterilized specimens were transferred onto a drop of sterilized PBS placed on a sterile microscope slide separately. The specimens were dissected under a dissecting microscope, and the midgut was removed. Genitalia was used to confirm the species identification referring to morphological features [14, 15], and only *Phlebotomus argentipes* was taken for the present experiment. The midgut of five sand flies were pooled in a 1.5 mL sterile microcentrifuge tube containing 150 *μ*L of sterile 1x PBS (pH 7.3) and homogenized using a disposable pestle. The lysate was diluted to 500 *μ*L with 1x PBS. From this stock solution, a dilution series of the lysate (10^0^-10^−9^) was prepared.

### 2.4. Culturing and Isolation of Bacteria

About 100 *μ*L of each lysate dilution was plated onto 25 mL of brain heart infusion (BHI) agar in petri dishes (9 cm in diameter). BHI was picked as a nonselective medium to promote the growth of microbes including nutritionally fastidious bacteria. Plates were incubated in the dark at 28°C for up to 2 weeks under aerobic conditions. The whole experiment procedure was repeated 10 times with 5 midgut pools of *P. argentipes*.

### 2.5. Morphological, Biochemical, and Physiological Characterization of Bacteria

A record of the phenotypically different colonies was used to determine the occurrence of bacteria in the midgut of each sand fly. These colonies were then subcultured to obtain a pure culture. All isolates were differentiated by Gram staining, biochemical tests, and morphological characterization. Gram's stains, endospore stains, acid fast stains, and motility testing were carried out along with aerobic and anaerobic growth testing. Oxidase, catalase, acid production [16], and O/F (oxidative and fermentative) tests were carried out to determine physiological characteristics [17, 18].

### 2.6. Identification of Isolated Bacteria by DNA Sequencing

Genomic DNA was extracted from individual colonies using a QIAmp DNA Mini Kit (Qiagen GmbH, Hilden, Germany) according to the manufacturer's instructions. Nearly 1000 bp of the bacterial 16S rRNA gene was amplified using universal primers 27F (5′ AGAGTTTGATCCTGGCTCAG 3′) and 1492R (5′ TACGGCTACCTTGTTACGACTT 3′) [19]. Polymerase chain reaction (PCR) amplification was carried out using a reaction mixture containing 1x PCR buffer (Invitrogen), 0.5 *μ*M of each primer, 2.5 mM MgCl_2_, 200 ng of purified DNA, 0.2 mM dNTPs, and 0.3 units of Taq polymerase (Invitrogen), and the total volume was adjusted to 25 *μ*L. The BHI agar media and ddH_2_O were used as negative controls.

Samples were amplified according to the following protocol: initial denaturation at 94°C for 10 min, followed by 35 cycles of denaturation at 95°C for 30 s, annealing at 57.5°C for 40 s, and extension at 72°C for 30 s. The final extension was at 72°C for 8 min. The PCR products were visualized on a 1% *w*/*v* agarose gel containing ethidium bromide using a UV transilluminator.

The PCR amplicons were purified using a QIAquick PCR Purification Kit (Qiagen), and purified products were sent to Macrogen, South Korea (Macrogen Inc., 1001, 254 Beotkkot-ro, Geumcheon-gu, Seoul, Republic of Korea) for 16S ribosomal RNA partial gene sequencing with the same 16S rRNA universal primers (27F and 1492R) by Sanger's method.

Sequencing results were analyzed by the BioEdit sequence alignment editor v7.0.9 software. The database search for homologous sequences was performed by submitting partial 16S rDNA sequences to the Basic Local Alignment Search Tool nucleotide (BLASTn) server of the National Centre for Biotechnology Information (NCBI, USA), using the 16S ribosomal RNA database (Bacteria and Archea) (http://blast.ncbi.nlm.nih.gov/Blast.cgi). The nucleotide similarity thresholds of the 16S rDNA sequences with the nearest neighbor at ≥95% and 97.5% [20] were considered as lower thresholds at the genus and species levels, respectively. Sequences were deposited in NCBI GenBank. Phylogenetic analyses were conducted according to the neighbor-joining method in MEGA7 [21].

## 3. Results

### 3.1. Entomological Investigation

A total of 1,969 specimens of sand flies were collected. Morphological identification revealed the presence of a single species, *P. argentipes*, which is reported to be the vector for cutaneous leishmaniasis in Sri Lanka. The male sand flies are the most represented with 91.4% (*n* = 1,800), whereas females with only 8.6% (*n* = 169) of the entire collection. Among these, 51 blood-fed females were identified from the collection (Table 1). The highest sand fly abundance was reported from the Polpithigama collection site with 84.7% (*n* = 1,668) of the entire collection followed by the Galgamuwa (11.1%, n=219) and Maho (4.2%, *n* = 82) sites.

### 3.2. Biochemical Characterization of Midgut Bacteria

**Figure 2:**
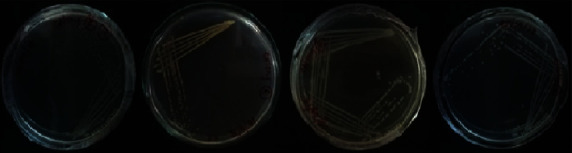
Some bacterial colonies isolated from the midgut grown on brain heart infusion (BHI) agar.

In the present study, a total of 50 randomly selected unfed female *P. argentipes* was examined (10 midgut pools of *P. argentipes*). The average colony forming unit (CFU) for the entire midgut content ranged from 8 × 10^1^ to 130 × 10^2^. There were more than 25 bacterial colonies with different morphological characters that were isolated from sand flies (Figure 2). The isolated organisms were first subjected to biochemical tests for identification up to the genus level. The morphological features of the bacteria colonies isolated from the midgut of sand flies and the results of the biochemical tests are illustrated in Table 2.

Only three strains were identified as gram-positive, and the majority of them were “Rod” shaped. All strains indicated aerobic growth except PaKu-20 and PaKu-23. Some strains such as PaKu-7, 8, 13, 14, 21, 22, 23, and, 25 indicated the ability to grow under anoxic conditions also. The catalase test was positive for all strains, and only PaKu-7, PaKu-8, PaKu-10, and PaKu-11 denoted oxidation ability (Table 2).

### 3.3. Molecular Characterization and Diversity of Midgut Bacteria

A total of 26 bacterial isolates were identified by comparing 16S rRNA partial sequences with those present in the NCBI GenBank. Sequences showed 99-100% identities to the existing database sequences. Isolated bacteria from the midguts of sand flies which were confirmed through molecular characterization are listed in Table 3.

A total of 19 bacterial species were encountered belonging to nine bacterial genera under two families mainly based on the results of 16S rRNA partial gene sequences. Out of the 19 bacterial species isolated from the sand fly midgut, 52.63% belonged to phyla Proteobacteria (*n* = 10) and the rest belonged to Firmicutes (47.37%; *n* = 9). Bacteria that are commonly associated with human infections such as *B. cereus*, *E. cloacae*, *Pseudomonas* spp., and *Staphylococcus* spp. were also recorded from local sand fly species *P. argentipes* investigated in this study. On the other hand, some rare species such as the *Rhizobium* species were also recorded from the present study along with some nonpathogenic organisms such as *B. megaterium*. The highest relative abundance of 15.38% was denoted with *Stenotrophomonas maltophilia* followed by *Bacillus subtilis* (7.69%), *Enterobacter cloacae* (7.69%), and *Aeromonas caviae* (7.69%). All the other species were encountered equally with a relative abundance of 3.85% (Table 4).

### 3.4. Phylogenetic Analysis of Midgut Bacteria Isolated from Sand Flies

**Figure 3:**
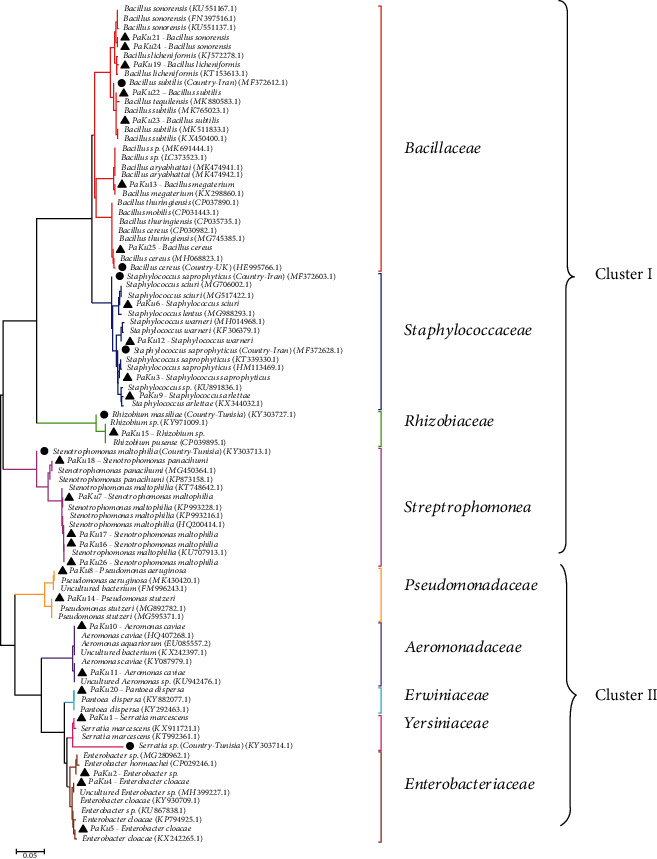
Phylogenetic analysis of gut microbiota isolated from *P. argentipes* sand flies verified by partial 16S rRNA gene sequences. Black circle—gut microbes in sand fly species—black triangle—study clones—gut microbes in sand fly (*P. argentipes*) in Sri Lanka. The sequences were aligned using Clustal Omega Software. The phylogenetic tree was constructed based on the neighbor-joining algorithm using MEGA7 software. Each bacterial family in the phylogenetic tree is represented by a separate colored line. The scale of the genetic distance is shown underneath.

The phylogenetic relationships of the bacteria and their corresponding taxonomic status at the family level are shown in Figure 3. Based on the phylogenetic tree in Figure 3, two main clusters were identified. The bacterial families Bacillaceae, Staphylacoccoceae, Rhizobiaceae, and Streptrophomonea were clustered together (cluster I), and the families Pseudomonadaceae, Aeromonadaceae, Erwiniaceae, Yersiniaceae, and Enterobacteriaceae were clustered together (cluster II).

Genetic distance was 0.02 between two main lineages of cluster I compared to that of cluster II with many sequences being related to existing sequences (Figure 3). Estimates of evolutionary divergence between sequences were analyzed using the Kimura 2-parameter model [22]. The rate variation among sites was modeled with a gamma distribution (shape parameter = 1). The analysis involved 91 nucleotide sequences, with 4,096 individual sequence comparisons. Codon positions included were 1st+2nd+3rd+Noncoding. There were a total of 1,590 positions in the final dataset after removing all ambiguous positions in each sequence pair (Supplementary file 1). The most frequently identified bacterial phylotype, *Stenotrophomonas maltophilia* (PaKu7, 16, 17, and 26), was 100% similar to the reference sequence HQ200414.1 obtained from the NCBI database (isolated from Kerala, in India) (Figure 3). Out of 4,096 sequence comparisons, 251 sequences were recorded to have less than 0.01 base substitutions per site and 1,119 sequence comparisons were recorded to have less than 0.10 base-pair substitutions per site.

The lengths in the phylogenetic tree branches were proportional to the differences between neighbors. Thus, the branch length represents the estimate of their evolutionary distance based on the multiple alignments of *n* positions, and *nd* estimates the total integer number of substitution events that occurred during the evolutionary divergence of the two sequences. Therefore, the phylogenetic relationship between these two *Rhizobium* species computed with the neighbor-joining method revealed that these two strains are closely related with a very short branch length (0.0166). Of the 4,096 sequences compared, about 251 were observed with less than 0.01 base substitutions per site and a comparison of 1119 sequences were observed with less than 0.10 base-pair substitutions per site.

## 4. Discussion

Leishmaniasis is considered one of the neglected tropical diseases. This has become a global public health issue in the world, with nearly 367 million estimated as being at risk [23]. However, only limited efforts have been made to interrupt the transmission of these diseases. In Sri Lanka, the first indigenous case was identified in 1992, which was of the cutaneous type [24]. At present, more than 2,000 cases have been identified from 2000 to 2009 and nearly 8,487 patients have been recorded during 2009-2016 representing at least one case from all 25 administrative districts [25]. In 2018, the caseload has been increased compared to the past years denoting cases from nonendemic regions.

Due to the lack of effective vaccines against the disease, vector control has become the main target to interrupt transmission. In view of the downsides allied with conventional mosquito control measures, such as the development of insecticide resistance, attempts to develop alternative methods to block the transmission of the *Leishmania* parasite are of paramount importance [23]. In some countries, novel vector control strategies such as paratransgenic strategies using commensal or symbiotic bacteria found in the mucosal sites of vectors have been evaluated [7, 26]. According to the previous literature, the presence of microorganisms in the gut of sand flies may impact the development of the *Leishmania* parasites [27]. It is also important to understand the establishment of microbiota in sand flies to clarify the underlying details of sand fly *Leishmania*-microbiota interactions [9]. However, in Sri Lanka, such attempts have rarely been made. Therefore, the present study was conducted to document the commensal bacterial species that inhabit the lumen of *P. argentipes*, the main vector for leishmaniasis transmission in Sri Lanka, to explore suitable candidates to be used for a paratransgenic strategy.

It is well known that there could be variations among midgut microbiota in the insects due to seasonal changes in the environment since varying temperatures may create alternate functional relationships between ectothermic animals and their microbiomes [28].

The present study records the presence of 20 midgut bacteria species. Some other studies have highlighted that more than 57% of the midgut bacteria species in *Lutzomyia* sp. belongs to Proteobacteria Phylum (Gram-negative bacteria) and 45% of Proteobacteria and 40% Firmicutes in other *Phlebotomus* species [29]. The present study denoted the occurrence of 81% of Proteobacteria and 19% of Firmicutes species in the midguts of *P. argentipes*. Such differences in these two previous studies may be due to the environmental changes and microhabitat conditions which may directly affect the occurrence of gut microbes [2]. However, the present study investigated midgut bacteria in a particular sand fly species. Therefore, the results from the present study cannot be directly compared with some of the previous studies as other studies have indicated the diversity among different sand fly species. Studies conducted in Tunisia and Turkey have stated that the diversity in the gut microbes of sand flies was higher among the adults collected from sheep sheds and rabbit holes [1, 9, 30]. Contamination of such environments with excreta of the animals may be the reason they express the higher diversity since animal excreta make the soil a fertile medium for the growth of coprophilic bacteria [30].

The current study observed the highest diversity of *Bacillu*s species recorded from *P. argentipes*, namely, *B. megaterium*, *B. licheniformis*, *B. sonorensis*, *B. subtilis*, and *B. cereus*, as compared to previous investigations conducted for *P. argentipes* [7, 22, 29] which denoted the higher abundance of *Enterobacteriaceae* in *P. argentipes* in India [11]. On the other hand, more *Bacillus* species have been recorded from *P. papatasi* in India [30].

Some studies have indicated the presence of *B. megaterium* [7, 29], which is considered a nonpathogenic bacteria that has potential use for paratransgenic manipulation of *P. argentipes*. The present investigation also detected this species from *P. argentipes*. Furthermore, it is important to note that *B. megaterium* and *B. flexus* were the only bacteria which have been recorded as nonpathogenic with some beneficial effects such as being used as prebiotics by two previous studies [15, 31]. *Bacillus megaterium* has been extensively used in biotechnological and genetic manipulation for the production of different molecules which are recognized to be harmless [23]. Therefore, *B. megaterium* can be used as a delivery vehicle to block vectorial transmission of *L. donovani* [23].

In India, *B. megaterium* has been marketed as a biofertilizer. It can promote cultivable plant growth and induce diseases in plants [11]. Furthermore, this species acts as a probiotic as well [15]. Therefore, the use of the transformed *B. megaterium* in soil may selectively colonize sand fly vectors and could be used by crude introduction to the suspected sand fly breeding habitats.

Species such as *S. marcescens* have been identified with an antileishmanial activity. The *S. marcescens* variant SM 365, a prodigiosin pigment producer has the potential to lyse *L. chagasi* parasites [32]. However, later work showed that lytic activity has no relationship with prodigiosin production and is not a determinant factor in the lysis of *L. braziliensis* upon interaction with *S. marcescens* [33]. Furthermore, *B. licheniformis* has also been recommended as a better candidate for paratransgenesis in *P. papatasi* because it is genetically tractable and can be used as a probiotic [30]. It has been identified as a strong oviposition inducer for gravid *P. papatas*i [34]. Therefore, the feasibility of these candidates as a paratransgenesis control strategy for leishmaniasis vectors should be further investigated.

The presence of *B. subtilis* in sand flies was firstly reported in India in 2008 (*P. argentipes*) [23] then in Tunisia in 2017 (*P. perniciosus*) [29]. This study reports the next evidence of the occurrence of *B. subtilis* in Old Word *P. argentipes.* This bacterium is a nonpathogenic *Bacillus* species which has been proposed as a possible candidate for the paratransgenic approach as it is nonpathogenic, easy to cultivate, and easy to genetically manipulate; its use for the paratransgenic control of *Leishmania* can be challenged by its capacity to establish long-term colonies in the gut of various sand fly species [29]. However, the suitability of *B. subtilis* in the paratransgenic approach may be questionable as this species may be associated with human infection in immunocompromised individuals and considered as a rare pathogen [35, 36].

Even though the species such as *E. cloacae* which were recorded from the current study has been used as a shuttle system to deliver, express, and spread foreign genes in termite colonies, this bacterium cannot be used for paratransgenic manipulation of sand flies since it is commonly associated with human infections [23]. *Stenotrophomonas maltophilia* has been identified as an important opportunistic pathogen and found to be in the gut microflora of sand flies [37]. This bacterium is commonly associated with aqueous habitats, plant rhizosphere, and animal food and water sources [38].

In this strategy of paratransgenesis, a commensal or symbiotic organism is genetically transformed to produce molecules that can kill the parasite. It has been successfully tested for the Chagas disease parasite *Trypanosoma cruzi* transmitted by a triatomine vector [38] and the vector of African sleeping sickness, *Glossina morsitans* [39, 40]. Therefore, further studies are crucial to identify the gut microorganisms in sand flies that could be used as potential candidates for paratransgenesis.

The present study also indicated the occurrence of *Staphylococcus saprophyticus*, *S. sciuri*, *S. arlettae*, *S. warneri*, *Serratia marcescens*, *Aeromonas caviae*, *Stenotrophomonas panacihumi*, *Bacillus licheniformis*, *B. sonorensis*, *Rhizobium* sp., and *B. subtilis* in *P. argentipes*, which is comparable to some previously published studies. It can be deduced that the presence of *S*. *saprophyticus* and *B. licheniformis* may be due to transtadial passage since these two species induce oviposition of gravid female sand flies [34, 41]. There has been evidence that chemicals such as hexanal, which is a byproduct of lipid oxidation and 2-methyl-2-butanol generated through microbial degradation, stimulate oviposition of gravid sand flies [41]. Therefore, the presence of these bacteria are probably due to the ingestion by larval stages from the oviposition site and their passage to nymphs and up to the adult sand flies [34].

The presence of *Rhizobium* species has never been found to be associated with the gut of *P. argentipes*. This species was first found among the *P. perniciosus* screened in Northern Tunisia [29]. Therefore, this study documents the first-ever observation of *Rhizobium* sp. in the midguts of *P. argentipes*.

The present study suffers from some limitations. The gut flora among insects is highly dynamic; therefore, this may influence the findings [42]. According to previous investigations, only 20% of the bacteria in the environment can be grown on culture media [43]. Hence, the presence of microorganisms on artificial cultures may not reflect the complete community structure inside the insect gut [38]. This has been defined in previous investigations also. Nucleic acid-based analysis such as Sanger sequencing, automated ribosomal internal transcribed spacer analysis (ARISA), terminal restriction fragment length polymorphism (T-RFLP), denaturing gradient gel electrophoresis (DGGE), and next-generation sequencing technology require a critical step that must combine an efficient cell disruption without DNA degradation and uniform nucleic acid extraction. The phylogenetic precision of species-level characterization of some bacterial genera is low with 16S rDNA gene sequencing even though it is being widely used for bacterial characterization [44]. However, some recent studies conducted in 2017 has indicated that the 16S rDNA sequencing revealed a highly diverse community composition that lost diversity as parasites developed into their metacyclic state and increased in abundance in infected flies [29]. Therefore, the use of the 16S rDNA region for the sequencing of available gut flora in sand flies is indispensable. Nevertheless, taking into account all the abovementioned limits and drawbacks, it is vital to gather basic knowledge on the occurrence of gut bacteria in the leishmaniasis vector of Sri Lanka, which was not been attempted previously. This may also motivate and encourage researchers to explore these aspects in the country and widen the research capacity.

Overall, the present investigation provides the first attempt to document the presence of bacteria in the midgut of *P. argentipes* sand flies in Sri Lanka. Therefore, this provides an insight into the potential use of symbiotic, nonpathogenic bacteria for the control vector-mediated transmission. However, the bacteria present in the digestive tract of one species may have a significant antiparasitic effect on *Leishmania* development, while gut flora composition may be a crucial factor for parasite growth in another vector species [9, 45].

## 5. Conclusion

*Phlebotomus argentipes* collected during the current study harbor a range of bacteria in their gut including *Rhizobium* sp., *B. megaterium*, *B. subtilis*, *E. cloacae*, and *S. marcescens.* Some are easy to manipulate and can be used in the generation of paratransgenic sand flies, while some have natural antileishmanial properties. Further studies must be focused on the transformation of the bacteria to express antileishmanial molecules, and further studies must also be focused on aspects related to field application.

## Figures and Tables

**Table 1:** Abundance of sand flies among different localities in Kurunegala District.

Collection site	Male	Female	Total
Maho	75	7	82
Galgamuwa	186	33	219
Polpithigama	1,539	129	1,668
*Total*	*1,800*	*169*	*1,969*

**Table 2:** Characterization of bacteria based on morphology and biochemical investigations.

Isolate no.	Gram's stain	Cell shape	Motility	Aerobic growth	Anaerobic growth	Oxidase	Catalase	Acid production	Oxidative/fermentative
PaKu-1	−	R	+	+	+	−	+	+	F
PaKu-2	−	R	+	+	+	−	+	+	F
PaKu-3	+	S	−	+	+	−	+	+	F
PaKu-4	−	R	D	+	+	−	+	+	F
PaKu-5	−	R	D	+	+	−	+	+	F
PaKu-6	+	S	−	+	+	−	+	+	F
PaKu-7	−	R	+	+	−	+	+	−	−
PaKu-8	−	R	+	+	−	+	+	−	−
PaKu-9	+	S	−	+	+	−	+	+	F
PaKu-10	−	R	+	+	+	+	+	+	F
PaKu-11	−	R	+	+	+	+	+	+	F
PaKu-12	+	S	−	+	+	−	+	+	F
PaKu-13	+	R	+	+	−	−	+	+	−
PaKu-14	−	R	+	+	−	−	+	+	O
PaKu-15	−	R	+	+	+	−	+	−	O
PaKu-16	−	R	+	+	+	−	+	−	−
PaKu-17	−	R	+	+	+	−	+	−	−
PaKu-18	+	R	+	+	+	−	−	−	−
PaKu-19	+	R	+	+	+	−	+	+	−
PaKu-20	−	R	−	+	+	−	+	+	F
PaKu-21	+	R	+	+	−	−	+	+	−
PaKu-22	+	R	+	+	−	−	+	+	F
PaKu-23	+	S	−	+	−	−	−	+	F
PaKu-24	+	R	+	+	+	−	+	+	−
PaKu-25	+	R	+	+	−	−	+	+	−
PaKu-26	−	R	+	+	+	−	+	−	−

R: rod; S: spirillum; +: positive; −: negative; F: fermentative; O: oxidative.

**Table 3:** Molecular identification of isolated bacteria from midgut of sand flies.

Phylum	Genus/species identification	Similarity (%)	Accession numbers	Isolate
Firmicutes	*Staphylococcus saprophyticus* ^∗^	100	MK841545	PaKu3
	*Staphylococcus sciuri* ^∗^	100	MK841316	PaKu6
	*Staphylococcus arlettae*	100	MK841329	PaKu9
	*Staphylococcus warneri* ^∗^	99	MK841411	PaKu12
	*Bacillus megaterium*	100	MK841412	PaKu13
	*Bacillus licheniformis*	99	MN067797	PaKu19
	*Bacillus sonorensis*	99	MN067799	PaKu21
	*Bacillus subtilis*	100	MN069586	PaKu22
	*Bacillus subtilis*	100	MN067800	PaKu23
	*Bacillus sonorensis*	99	MN067801	PaKu24
	*Bacillus cereus*	100	MN067802	PaKu25

Proteobacteria	*Serratia marcescens* ^∗^	99	MK841543	PaKu1
	*Enterobacter* sp.^∗^	100	MK841544	PaKu2
	*Enterobacter cloacae* ^∗^	99	MK841569	PaKu4
	*Enterobacter cloacae* ^∗^	100	MK841570	PaKu5
	*Stenotrophomonas maltophilia* ^!^	100	MK841317	PaKu7
	*Pseudomonas aeruginosa* ^∗^	100	MK841321	PaKu8
	*Aeromonas caviae*	100	MK841331	PaKu10
	*Aeromonas caviae*	100	MK841333	PaKu11
	*Pseudomonas stutzeri*	100	MN067779	PaKu14
	*Rhizobium* sp.	100	MN067780	PaKu15
	*Stenotrophomonas maltophilia* ^!^	100	MN067781	PaKu16
	*Stenotrophomonas maltophilia* ^!^	100	MN067783	PaKu17
	*Stenotrophomonas panacihumi*	100	MN067796	PaKu18
	*Stenotrophomonas maltophilia* ^!^	100	MN069585	PaKu26
	*Pantoea dispersa*	99	MN067798	PaKu20

^∗^Human pathogens. ^**!**^Rarely pathogenic on humans.

**Table 4:** Relative abundance of midgut bacteria encountered in different sand fly pools.

Genus/species identification	Relative abundance (%) of the organism in the tested insect pools (*n*)
*Staphylococcus saprophyticus*	3.85 (1)
*Staphylococcus sciuri*	3.85 (1)
*Staphylococcus arlettae*	3.85 (1)
*Staphylococcus warneri*	3.85 (1)
*Bacillus megaterium*	3.85 (1)
*Bacillus licheniformis*	3.85 (1)
*Bacillus sonorensis*	3.85 (1)
*Bacillus subtilis*	7.69 (2)
*Bacillus sonorensis*	3.85 (1)
*Bacillus cereus*	3.85 (1)
*Serratia marcescen*	3.85 (1)
*Enterobacter* sp.	3.85 (1)
*Enterobacter cloacae*	7.69 (2)
*Stenotrophomonas maltophilia*	15.38 (4)
*Pseudomonas aeruginosa*	3.85 (1)
*Aeromonas caviae*	7.69 (2)
*Pseudomonas stutzeri*	3.85 (1)
*Rhizobium* sp.	3.85 (1)
*Stenotrophomonas panacihumi*	3.85 (1)
*Pantoea dispersa*	3.85 (1)

## Data Availability

All the data generated during this study will be available from the corresponding author upon reasonable request.

## Abbreviations

MOH: Medical Officer of Health
PBS: Phosphate-buffered saline
BHI: Brain heart infusion
RIDL: Release of insects carrying a dominant lethal
SIT: Sterile insect technique
CBNT: Cattle-baited net traps
PCR: Polymerase chain reaction.

## Supplementary Materials

Supplementary Materials Sequence alignment of midgut bacteria encountered from *Phlebotomus argentipes* caught from the wild.
