# Supplementary material for: The Diversity of Midgut Bacteria among Wild-Caught Phlebotomus argentipes (Psychodidae: Phlebotominae), the Vector of Leishmaniasis in Sri Lanka
Source: Biomed Res Int. 2020 Aug 19;2020:5458063. doi: 10.1155/2020/5458063 (PMC7453272; doi:10.1155/2020/5458063)
Supplement: Supplementary Materials — Sequence alignment of midgut bacteria encountered from Phlebotomus argentipes caught from the wild. [file 5458063.f1.docx]

**Supplementary material 1:** Sequence alignment of midgut bacteria isolated from the field caught *Phlebotomus argentipes*

PaKu1_-_Serratia_marcescens ---------- ---------- ----GGCTCA GATTGAACGC TGGCGGCAGG CTTAACACAT GCAAGTCGAG CGGTAGCACA AGGG-AGCTT GCTCCCCCGG GTGACGAGCG GCGGACGGGT GAGTAATGTC TGGG-AAACT GCCTGATGGA GGGGGATAAC TACTGGAAAC GGTAGCTAAT ACCGCAT--- -------AAC

Serratia_marcescens_(KX911721. ---------- ---------- ---AGGCTCA GATTGAACGC TGGCGGCAGG CTTAACACAT GCAAGTCGAG CGGTAGCACA AGGG-AGCTT GCTCCCC-GG GTGACGAGCG GCGGACGGGT GAGTAATGTC TGGG-AAACT GCCTGATGGA GGGGGATAAC TACTGGAAAC GGTAGCTAAT ACCGCAT--- -------AAC

Serratia_marcescens_(KT992361. ---------- -AGAGTTTGA TCATGGCTCA GATTGAACGC TGGCGGCAGG CTTAACACAT GCAAGTCGAG CGGTAGCACA AGGG-AGCTT GCTCCCT-GG GTGACGAGCG GCGGACGGGT GAGTAATGTC TGGG-AAACT GCCTGATGGA GGGGGATAAC TACTGGAAAC GGTAGCTAAT ACCGCAT--- -------AAC

PaKu2_-_Enterobacter_sp. ---------- ---------- -----GCTCA GATTGAACGC TGGCGGCAGG CCTAACACAT GCAAGTCGAA CGGTAACAGG AAGC-AGCTT GCTGCTT-CG CTGACGAGTG GCGGACGGGT GAGTAATGTC TGGG-AAACT GCCTGATGGA GGGGGATAAC TACTGGAAAC GGTAGCTAAT ACCGCAT--- -------AAC

Enterobacter_sp._(MG280962.1) ---------- ---------- -----GCTCA GATTGAACGC TGGCGGCAGG CCTAACACAT GCAAGTCGAA CGGTAACAGG AAGC-AGCTT GCTGCTT-CG CTGACGAGTG GCGGACGGGT GAGTAATGTC TGGG-AAACT GCCTGATGGA GGGGGATAAC TACTGGAAAC GGTAGCTAAT ACCGCAT--- -------AAC

Enterobacter_hormaechei_(CP029 ---------- ---------- -----GCTCA GATTGAACGC TGGCGGCAGG CCTAACACAT GCAAGTCGAA CGGTAACAGG AAGC-AGCTT GCTGCTT-CG CTGACGAGTG GCGGACGGGT GAGTAATGTC TGGG-AAACT GCCTGATGGA GGGGGATAAC TACTGGAAAC GGTAGCTAAT ACCGCAT--- -------AAC

PaKu3_-_Staphylococcus_saproph ---------- ---------- ---------- --ATGAACGC TGGCGGCGTG CCTAATACAT GCAAGTCGAG CGAACAGATA AGGA--GCTT GCTCCTT--T GACGTTAGCG GCGGACGGGT GAGTAACACG TGGGTAACCT ACCTATAAGA CTGGGATAAC TTCGGGAAAC CGGAGCTAAT ACCGGATAAC ATTTGGAACC

Staphylococcus_saprophyticus_( ---------- ---------- ---------A GGATGAACGC TGGCGGCGTG CCTAATACAT GCAAGTCGAG CGAACAGATA AGGA--GCTT GCTCCTT--T GACGTTAGCG GCGGACGGGT GAGTAACACG TGGGTAACCT ACCTATAAGA CTGGGATAAC TTCGGGAAAC CGGAGCTAAT ACCGGATAAC ATTTGGAACC

Staphylococcus_saprophyticus_( ---------- -AGAGTTTGA TCCTGGCTCA GGATGAACGC TGGCGGCGTG CCTAATACAT GCAAGTCGAG CGAACAGATA AGGA--GCTT GCTCCTT--T GACGTTAGCG GCGGACGGGT GAGTAACACG TGGGTAACCT ACCTATAAGA CTGGGATAAC TTCGGGAAAC CGGAGCTAAT ACCGGATAAC ATTTGGAACC

PaKu4_-_Enterobacter_cloacae ---------- ---------- ------CTCA GATTGAACGC TGGCGGCAGG CATAACACAT GCAAGTCGAA CGGTAGCACA GAG--AGCTT GCT-CTC-GG GTGACGAGTG GCGGACGGGT GAGTAATGTC TGGG-AAACT GCCTGATGGA GGGGGATAAC TACTGGAAAC GGTAGCTAAT ACCGCAT--- -------AAC

Uncultured_Enterobacter_sp._(M ---------- AGAGTTTGA- TCATGGCTCA GATTGAACGC TGGCGGCAGG CCTAACACAT GCAAGTCGAA CGGTAGCACA GAG--AGCTT GCT-CTC-GG GTGACGAGTG GCGGACGGGT GAGTAATGTC TGGG-AAACT GCCTGATGGA GGGGGATAAC TACTGGAAAC GGTAGCTAAT ACCGCAT--- -------AAC

Enterobacter_cloacae_(KY930709 ---------- --AGTTTGGA TCCTGGCTCA GATTGAACGC TGGCGGCAGG CCTAACACAT GCAAGTCGAA CGGTAGCACA GAG--AGCTT GCT-CTC-GG GTGACGAGTG GCGGACGGGT GAGTAATGTC TGGG-AAACT GCCTGATGGA GGGGGATAAC TACTGGAAAC GGTAGCTAAT ACCGCAT--- -------AAC

PaKu5_-_Enterobacter_cloacae ---------- ---------- ------CTCA GATTGAACGC TGGCGGCAGG CCTAACACAT GCAAGTCGAA CGGTAGCACA GAG--AGCTT GCT-CTC-GG GTGACGAGTG GCGGACGGGT GAGTAATGTC TGGG-AAACT GCCTGATGGA GGGGGATAAC TACTGGAAAC GGTAGCTAAT ACCGCAT--- -------AAT

Enterobacter_sp._(KU867838.1) ---------- ---------- -CCTGGCTCA GATTGAACGC TGGCGGCAGG CCTAACACAT GCAAGTCGAA CGGTAGCACA GAG--AGCTT GCT-CTC-GG GTGACGAGTG GCGGACGGGT GAGTAATGTC TGGG-AAACT GCCTGATGGA GGGGGATAAC TACTGGAAAC GGTAGCTAAT ACCGCAT--- -------AAT

Enterobacter_cloacae_(KX242265 ---------T AGAGTTTTAG AACAGGCTCA GATTGAACGC TGGCGGCAGG CCTAACACAT GCAAGTCGAA CGGTAGCACA GAG--AGCTT GCT-CTC-GG GTGACGAGTG GCGGACGGGT GAGTAATGTC TGGG-AAACT GCCTGATGGA GGGGGATAAC TACTGGAAAC GGTAGCTAAT ACCGCAT--- -------AAC

Enterobacter_cloacae_(KP794925 ---------- AGAGTTTGAA TCATGGCTCA GATTGAACGC TGGCGGCAGG CCTAACACAT GCAAGTCGAA CGGTAGCACA GAG--AGCTT GCT-CTC-GG GTGACGAGTG GCGGACGGGT GAGTAATGTC TGGG-AAACT GCCTGATGGA GGGGGATAAC TACTGGAAAC GGTAGCTAAT ACCGCAT--- -------AAT

PaKu6_-_Staphylococcus_sciuri ---------- ---------- -------TCA GGATGAACGC TGGCGGCGTG CCTAATACAT GCAAGTCGAG CGAACAGATG AGAA--GCTT GCTTCTC--T GATGTTAGCG GCGGACGGGT GAGTAACACG TGGGTAACCT ACCTATAAGA CTGGGATAAC TCCGGGAAAC CGGGGCTAAT ACCGGATAAT ATTTTGAACC

Staphylococcus_sciuri_(MG70600 ---TTTTATG GAGAGTTTGA TCCTGGCTCA GGATGAACGC TGGCGGCGTG CCTAATACAT GCAAGTCGAG CGAACAGATG AGAA--GCTT GCTTCTC--T GATGTTAGCG GCGGACGGGT GAGTAACACG TGGGTAACCT ACCTATAAGA CTGGGATAAC TCCGGGAAAC CGGGGCTAAT ACCGGATAAT ATTTTGAACC

Staphylococcus_lentus_(MG98829 ---------T TAGAGTTTTG AACAGGCTCA GGATGAACGC TGGCGGCGTG CCTAATACAT GCAAGTCGAG CGAACAGATG AGAA--GCTT GCTTCTC--T GATGTTAGCG GCGGACGGGT GAGTAACACG TGGGTAACCT ACCTATAAGA CTGGGATAAC TCCGGGAAAC CGGGGCTAAT ACCGGATAAT ATTTTGAACC

Staphylococcus_sciuri_(MG51742 ---TTTTATG GAGAGTTTGA TCCTGGCTCA GGATGAACGC TGGCGGCGTG CCTAATACAT GCAAGTCGAG CGAACAGATG AGAA--GCTT GCTTCTC--T GATGTTAGCG GCGGACGGGT GAGTAACACG TGGGTAACCT ACCTATAAGA CTGGGATAAC TCCGGGAAAC CGGGGCTAAT ACCGGATAAT ATTTTGAACC

PaKu8_-_Pseudomonas_aeruginosa ---------- ---------- ----GGCTCA GATTGAACGC TGGCGGCAGG CCTAACACAT GCAAGTCGAG CGGATGAAGG GAG----CTT GCTCCTG--- -GATTCAGCG GCGGACGGGT GAGTAATGCC TAGG-AATCT GCCTGGTAGT GGGGGATAAC GTCCGGAAAC GGGCGCTAAT ACCGCAT--- -------ACG

Uncultured_bacterium_(FM996243 ---------- ---------- ---TGGCTCA GATTGAACGC TGGCGGCAGG CCTAACACAT GCAAGTCGAG CGGATGAAGG GAG----CTT GCTCCTG--- -GATTCAGCG GCGGACGGGT GAGTAATGCC TAGG-AATCT GCCTGGTAGT GGGGGATAAC GTCCGGAAAC GGGCGCTAAT ACCGCAT--- -------ACG

Pseudomonas_aeruginosa_(MK4304 ---------- -AGAGTTTGA TCCTGGCTCA GATTGAACGC TGGCGGCAGG CCTAACACAT GCAAGTCGAG CGGATGAAGG GAG----CTT GCTCCTG--- -GATTCAGCG GCGGACGGGT GAGTAATGCC TAGG-AATCT GCCTGGTAGT GGGGGATAAC GTCCGGAAAC GGGCGCTAAT ACCGCAT--- -------ACG

PaKu9_-_Staphylococcus_arletta ---------- ---------- -------TCA GGATGAACGC TGGCGGCGTG CCTAATACAT GCAAGTCGAG CGAACAGATA AGGA--GCTT GCTCCTT--T GACGTTAGCG GCGGACGGGT GAGTAACACG TGGGTAACCT ACCTATAAGA CTGGAATAAC TCCGGGAAAC CGGGGCTAAT GCCGGATAAC ATTTAGAACC

Staphylococcus_arlettae_(KX344 ---------- -GAGTTTTGA TTCTGGCTCA GGATGAACGC TGGCGGCGTG CCTAATACAT GCAAGTCGAG CGAACAGATA AGGA--GCTT GCTCCTT--T GACGTTAGCG GCGGACGGGT GAGTAACACG TGGGTAACCT ACCTATAAGA CTGGAATAAC TCCGGGAAAC CGGGGCTAAT GCCGGATAAC ATTTAGAACC

Staphylococcus_sp._(KU891836.1 ---------- -AGAGTTTGA TCCTGGCTCA GGATGAACGC TGGCGGCGTG CCTAATACAT GCAAGTCGAG CGAACAGATA AGGA--GCTT GCTCCTT--T GACGTTAGCG GCGGACGGGT GAGTAACACG TGGGTAACCT ACCTATAAGA CTGGAATAAC TCCGGGAAAC CGGGGCTAAT GCCGGATAAC ATTTAGAACC

PaKu10_-_Aeromonas_caviae ---------- ---------- -----GCTCA GATTGAACGC TGGCGGCAGG CCTAACACAT GCAAGTCGAG CGGCAGCGGG AAAGTAGCTT GCTACTTTTG CCGGCGAGCG GCGGACGGGT GAGTAATGCC TGGG-AAATT GCCCAGTCGA GGGGGATAAC AGTTGGAAAC GACTGCTAAT ACCGCAT--- -------ACG

Uncultured_bacterium_(KX242397 ---------- --------GA TCATGGCTCA GATTGAACGC TGGCGGCAGG CCTAACACAT GCAAGTCGAG CGGCAGCGGG AAAGTAGCTT GCTACTTTTG CCGGCGAGCG GCGGACGGGT GAGTAATGCC TGGG-AAATT GCCCAGTCGA GGGGGATAAC AGTTGGAAAC GACTGCTAAT ACCGCAT--- -------ACG

Aeromonas_caviae_(HQ407268.1) ---------- -AGAGTTTGA TCATGGCTCA GATTGAACGC TGGCGGCAGG CCTAACACAT GCAAGTCGAG CGGCAGCGGG AAAGTAGCTT GCTACTTTTG CCGGCGAGCG GCGGACGGGT GAGTAATGCC TGGG-AAATT GCCCAGTCGA GGGGGATAAC AGTTGGAAAC GACTGCTAAT ACCGCAT--- -------ACG

Aeromonas_aquariorum_(EU085557 ---------- --GAGTTTGA TCATGGCTCA GATTGAACGC TGGCGGCAGG CCTAACACAT GCAAGTCGAG CGGCAGCGGG AAAGTAGCTT GCTACTTTTG CCGGCGAGCG GCGGACGGGT GAGTAATGCC TGGG-AAATT GCCCAGTCGA GGGGGATAAC AGTTGGAAAC GACTGCTAAT ACCGCAT--- -------ACG

PaKu11_-_Aeromonas_caviae ---------- ---------- ------CTCA GATTGAACGC TGGCGGCAGG CCTAACACAT GCAAGTCGAG CGGCAGCGGG AAAGTAGCTT GCTACTTTTG CCGGCGAGCG GCGGACGGGT GAGTAATGCC TGGG-AAATT GCCCAGTCGA GGGGGATAAC AGTTGGAAAC GACTGCTAAT ACCGCAT--- -------ACG

Aeromonas_caviae_(KY087979.1) ---------- ---------- ----GGCTCA GATTGAACGC TGGCGGCAGG CCTAACACAT GCAAGTCGAG CGGCAGCGGG AAAGTAGCTT GCTACTTTTG CCGGCGAGCG GCGGACGGGT GAGTAATGCC TGGG-AAATT GCCCAGTCGA GGGGGATAAC AGTTGGAAAC GACTGCTAAT ACCGCAT--- -------ACG

Uncultured_Aeromonas_sp._(KU94 ---------- ---------- ----GGCTCA GATTGAACGC TGGCGGCAGG CCTAACACAT GCAAGTCGAG CGGCAGCGGG AAAGTAGCTT GCTACTTTTG CCGGCGAGCG GCGGACGGGT GAGTAATGCC TGGG-AAATT GCCCAGTCGA GGGGGATAAC AGTTGGAAAC GACTGCTAAT ACCGCAT--- -------ACG

PaKu12_-_Staphylococcus_warner ---------- ---------- -----GCTCA GGATGAACGC TGGCGGCGTG CCTAATACAT GCAAGTCGAG CGAACAGATA AGGA--GCTT GCTCCTT--T GACGTTAGCG GCGGACGGGT GAGTAACACG TGGATAACCT ACCTATAAGA CTGGGATAAC TTCGGGAAAC CGGAGCTAAT ACCGGATAAC ATATTGAACC

Staphylococcus_warneri_(MH0149 ---------- -AGAGTTTGA TCATGGCTCA GGATGAACGC TGGCGGCGTG CCTAATACAT GCAAGTCGAG CGAACAGATA AGGA--GCTT GCTCCTT--T GACGTTAGCG GCGGACGGGT GAGTAACACG TGGATAACCT ACCTATAAGA CTGGGATAAC TTCGGGAAAC CGGAGCTAAT ACCGGATAAC ATATTGAACC

Staphylococcus_warneri_(KF3063 ---------- TAGAGTTTGA TCATGGCTCA GAATGAACGC TGGCGGCGTG CCTAATACAT GCAAGTCGAG CGAACAGATA AGGA--GCTT GCTCCTT--T GACGTTAGCG GCGGACGGGT GAGTAACACG TGGATAACCT ACCTATAAGA CTGGGATAAC TTCGGGAAAC CGGAGCTAAT ACCGGATAAC ATATTGAACC

PaKu13_-_Bacillus_megaterium ---------- ---------- ---------- -GATGAACGC TGGCGGCGTG CCTAATACAT GCAAGTCGAG CGAACTGATT AGAA--GCTT GCTTCTA--T GACGTTAGCG GCGGACGGGT GAGTAACACG TGGGCAACCT GCCTGTAAGA CTGGGATAAC TTCGGGAAAC CGAAGCTAAT ACCGGATAGG ATCTTCTCCT

Bacillus_sp._(MK691444.1) ---------- -AGAGTTTGA TCCTGGCTCA GGATGAACGC TGGCGGCGTG CCTAATACAT GCAAGTCGAG CGAACTGATT AGAA--GCTT GCTTCTA--T GACGTTAGCG GCGGACGGGT GAGTAACACG TGGGCAACCT GCCTGTAAGA CTGGGATAAC TTCGGGAAAC CGAAGCTAAT ACCGGATAGG ATCTTCTCCT

Bacillus_sp._(LC373523.1) ---------- -AGAGTT-GA TCCTGGCTCA GGATGAACGC TGGCGGCGTG CCTAATACAT GCAAGTCGAG CGAACTGATT AGAA--GCTT GCTTCTA--T GACGTTAGCG GCGGACGGGT GAGTAACACG TGGGCAACCT GCCTGTAAGA CTGGGATAAC TTCGGGAAAC CGAAGCTAAT ACCGGATAGG ATCTTCTCCT

Bacillus_aryabhattai_(MK474942 ---------- ---------- ----GGCTCA GGATGAACGC TGGCGGCGTG CCTAATACAT GCAAGTCGAG CGAACTGATT AGAA--GCTT GCTTCTA--T GACGTTAGCG GCGGACGGGT GAGTAACACG TGGGCAACCT GCCTGTAAGA CTGGGATAAC TTCGGGAAAC CGAAGCTAAT ACCGGATAGG ATCTTCTCCT

Bacillus_aryabhattai_(MK474941 ---------- ---------- TCCTGGCTCA GGATGAACGC TGGCGGCGTG CCTAATACAT GCAAGTCGAG CGAACTGATT AGAA--GCTT GCTTCTA--T GACGTTAGCG GCGGACGGGT GAGTAACACG TGGGCAACCT GCCTGTAAGA CTGGGATAAC TTCGGGAAAC CGAAGCTAAT ACCGGATAGG ATCTTCTCCT

Bacillus_megaterium_(KX298860. ---------- ---------- -CCCTGCTCA GGATGAACGC TGGCGGCGTG CCTAATACAT GCAAGTCGAG CGAACTGATT AGAA--GCTT GCTTCTA--T GACGTTAGCG GCGGACGGGT GAGTAACACG TGGGCAACCT GCCTGTAAGA CTGGGATAAC TTCGGGAAAC CGAAGCTAAT ACCGGATAGG ATCTTCTCCT

PaKu14_-_Pseudomonas_stutzeri ---------- ---------- ------CTCA GATTGAACGC TGGCGGCAGG CCTAACACAT GCAAGTCGAG CGGATGAGTG GAG----CTT GCTCCAT--- -GATTCAGCG GCGGACGGGT GAGTAATGCC TAGG-AATCT GCCTGGTAGT GGGGGACAAC GTTTCGAAAG GAACGCTAAT ACCGCAT--- -------ACG

Pseudomonas_stutzeri_(MG892782 ---------- ---------- ----AGCTCA GATTGAACGC TGGCGGCAGG CCTAACACAT GCAAGTCGAG CGGATGAGTG GAG----CTT GCTCCAT--- -GATTCAGCG GCGGACGGGT GAGTAATGCC TAGG-AATCT GCCTGGTAGT GGGGGACAAC GTTTCGAAAG GAACGCTAAT ACCGCAT--- -------ACG

Pseudomonas_stutzeri_(MG595371 ---------- ---------- ----AGCTCA GATTGAACGC TGGCGGCAGG CCTAACACAT GCAAGTCGAG CGGATGAGTG GAG----CTT GCTCCAT--- -GATTCAGCG GCGGACGGGT GAGTAATGCC TAGG-AATCT GCCTGGTAGT GGGGGACAAC GTTTCGAAAG GAACGCTAAT ACCGCAT--- -------ACG

PaKu15_-_Rhizobium_sp. ---------- ---------- -------TCA GAACGAACGC TGGCGGCAGG CTTAACACAT GCAAGTCGAA CGC------- ---------- ---CCCG--C AAGGGGAGTG GCAGACGGGT GAGTAACGCG TGGG-AACAT ACCCTTTCCT GCGGAATAGC TCCGGGAAAC TGGAATTAAT ACCGCAT--- ----------

Rhizobium_pusense_(CP039895.1) ---------- ---------- -------TCA GAACGAACGC TGGCGGCAGG CTTAACACAT GCAAGTCGAA CGC------- ---------- ---CCCG--C AAGGGGAGTG GCAGACGGGT GAGTAACGCG TGGG-AACAT ACCCTTTCCT GCGGAATAGC TCCGGGAAAC TGGAATTAAT ACCGCAT--- ----------

Rhizobium_sp._(KY971009.1) -------CAA CAGAGTTTGA TCCTGGCTCA GAACGAACGC TGGCGGCAGG CTTAACACAT GCAAGTCGAA CGC------- ---------- ---CCCG--C AAGGGGAGTG GCAGACGGGT GAGTAACGCG TGGG-AACAT ACCCTTTCCT GCGGAATAGC TCCGGGAAAC TGGAATTAAT ACCGCAT--- ----------

PaKu7_-_Stenotrophomonas_malto ---------- ---------- -----GCTCA GAGTGAACGC TGGCGGTAGG CCTAACACAT GCAAGTCGAA CGGCAGCACA G-GAGAGCTT GCTCTC-TGG GTGGCGAGTG GCGGACGGGT GAGGAATACA TCGG-AATCT ACTCTGTCGT GGGGGATAAC GTAGGGAAAC TTACGCTAAT ACCGCAT--- -------ACG

Stenotrophomonas_maltophilia_( ---------- TAGAGTTTGA TCCTGGCTCA GAGTGAACGC TGGCGGTAGG CCTAACACAT GCAAGTCGAA CGGCAGCACA G-GAGAGCTT GCTCTC-TGG GTGGCGAGTG GCGGACGGGT GAGGAATACA TCGG-AATCT ACTCTGTCGT GGGGGATAAC GTAGGGAAAC TTACGCTAAT ACCGCAT--- -------ACG

Stenotrophomonas_maltophilia_( ---------C CTGAGTTTGA TCCTGGCTCA GAGTGAACGC TGGCGGTAGG CCTAACACAT GCAAGTCGAA CGGCAGCACA G-GAGAGCTT GCTCTC-TGG GTGGCGAGTG GCGGACGGGT GAGGAATACA TCGG-AATCT ACTCTGTCGT GGGGGATAAC GTAGGGAAAC TTACGCTAAT ACCGCAT--- -------ACG

PaKu16_-_Stenotrophomonas_malt ---------- ---------- ---------A GAGTGAACGC TGGCGGTAGG CCTAACACAT GCAAGTCGAA CGGCAGCACA G-GAGAGCTT GCTCTC-TGG GTGGCGAGTG GCGGACGGGT GAGGAATACA TCGG-AATCT ACTCTGTCGT GGGGGATAAC GTAGGGAAAC TTACGCTAAT ACCGCAT--- -------ACG

Stenotrophomonas_maltophilia_( ---------- ---------- -------TCA GAGTGAACGC TGGCGGTAGG CCTAACACAT GCAAGTCGAA CGGCAGCACA G-GAGAGCTT GCTCTC-TGG GTGGCGAGTG GCGGACGGGT GAGGAATACA TCGG-AATCT ACTCTGTCGT GGGGGATAAC GTAGGGAAAC TTACGCTAAT ACCGCAT--- -------ACG

Stenotrophomonas_maltophilia_( ---------- ------TTGA TCCTGGCTCA GAGTGAACGC TGGCGGTAGG CCTAACACAT GCAAGTCGAA CGGCAGCACA G-GAGAGCTT GCTCTC-TGG GTGGCGAGTG GCGGACGGGT GAGGAATACA TCGG-AATCT ACTCTGTCGT GGGGGATAAC GTAGGGAAAC TTACGCTAAT ACCGCAT--- -------ACG

PaKu17_-_Stenotrophomonas_malt ---------- ---------- ---------A GAGTGAACGC TGGCGGTAGG CCTAACACAT GCAAGTCGAA CGGCAGCACA G-GAGAGCTT GCTCTC-TGG GTGGCGAGTG GCGGACGGGT GAGGAATACA TCGG-AATCT ACTCTGTCGT GGGGGATAAC GTAGGGAAAC TTACGCTAAT ACCGCAT--- -------ACG

PaKu26_-_Stenotrophomonas_malt ---------- ---------- ---------A GAGTGAACGC TGGCGGTAGG CCTAACACAT GCAAGTCGAA CGGCAGCACA G-GAGAGCTT GCTCTC-TGG GTGGCGAGTG GCGGACGGGT GAGGAATACA TCGG-AATCT ACTCTGTCGT GGGGGATAAC GTAGGGAAAC TTACGCTAAT ACCGCAT--- -------ACG

Stenotrophomonas_maltophilia_( ---------- --AGAGTTGA TCCTGGCTCA GAGTGAACGC TGGCGGTAGG CCTAACACAT GCAAGTCGAA CGGCAGCACA G-GAGAGCTT GCTCTC-TGG GTGGCGAGTG GCGGACGGGT GAGGAATACA TCGG-AATCT ACTCTGTCGT GGGGGATAAC GTAGGGAAAC TTACGCTAAT ACCGCAT--- -------ACG

PaKu18_-_Stenotrophomonas_pana ---------- ---------- ---------A GAGTGAACGC TGGCGGCAGG CCTAACACAT GCAAGTCGAA CGGCAGCACA GTAAGAGCTT GCTCTTATGG GTGGCGAGTG GCGGACGGGT GAGGAATACA TCGG-AATCT ACCTTTTCGT GGGGGATAAC GTAGGGAAAC TTACGCTAAT ACCGCAT--- -------ACG

Stenotrophomonas_panacihumi_(M ---------- ---------- ------CTCA GAGTGAACGC TGGCGGCAGG CCTAACACAT GCAAGTCGAA CGGCAGCACA GTAAGAGCTT GCTCTTATGG GTGGCGAGTG GCGGACGGGT GAGGAATACA TCGG-AATCT ACCTTTTCGT GGGGGATAAC GTAGGGAAAC TTACGCTAAT ACCGCAT--- -------ACG

Stenotrophomonas_panacihumi_(K -----CCCTC TTGAGTTTGA TCCTGGCTCA GAGTGAACGC TGGCGGCAGG CCTAACACAT GCAAGTCGAA CGGCAGCACA G-GAGAGCTT GCTCTC-TGG GTGGCGAGTG GCGGACGGGT GAGGAATACA TCGG-AATCT ACCTTTTCGT GGGGGATAAC GTAGGGAAAC TTACGCTAAT ACCGCAT--- -------ACG

PaKu19_-_Bacillus_licheniformi ---------- ---------- --------TC AGACGAACGC TGGCGGCGTG CTTAATACAT GCAAGTCGAG CGGACCGAC- GGGA--GCTT GCTCCC---T TAGGTCAGCG GCGGACGGGT GAGTAACACG TGGGTAACCT GCCTGTAAGA CTGGGATAAT TCCGGGAAAC CGGGGCTAAT ACCGGATGCT TGATTGAACC

Bacillus_licheniformis_(KJ5722 ---------- ---------- ---------A GGACGAACGC TGGCGGCGTG CTTAATACAT GCAAGTCGAG CGGACCGAC- GGGA--GCTT GCTCCC---T TAGGTCAGCG GCGGACGGGT GAGTAACACG TGGGTAACCT GCCTGTAAGA CTGGGATAAT TCCGGGAAAC CGGGGCTAAT ACCGGATGCT TGATTGAACC

Bacillus_licheniformis_(KT1536 ---------- ---------- ---------- -GACGAACGC TGGCGGCGTG CCTAATACAT GCAAGTCGAG CGGACCGAC- GGGA--GCTT GCTCCC---T TAGGTCAGCG GCGGACGGGT GAGTAACACG TGGGTAACCT GCCTGTAAGA CTGGGATAAC TCCGGGAAAC CGGGGCTAAT ACCGGATGCT TGATTGAACC

PaKu20_-__Pantoea_dispersa ---------- ---------- ---------A GATTGAACGC TGGCGGCAGG CCTAACACAT GCAAGTCGAA CGGCAGCACA GAAG-AGCTT GCTCTTT-GG GTGGCGAGTG GCGGACGGGT GAGTAATGTC TGGG-AAACT GCCCGATGGA GGGGGATAAC TACTGGAAAC GGTAGCTAAT ACCGCAT--- -------AAC

Pantoea_dispersa_(KY292463.1) ---------- ---------- -CCTGGCTCA GATTGAACGC TGGCGGCAGG CCTAACACAT GCAAGTCGAA CGGCAGCACA GAAG-AGCTT GCTCTTT-GG GTGGCGAGTG GCGGACGGGT GAGTAATGTC TGGG-AAACT GCCCGATGGA GGGGGATAAC TACTGGAAAC GGTAGCTAAT ACCGCAT--- -------AAC

Pantoea_dispersa_(KY882077.1) ---------- ---------- -----GCTCA GATTGAACGC TGGCGGCAGG CCTAACACAT GCAAGTCGAA CGGCAGCACA GAAG-AGCTT GCTCTTT-GG GTGGCGAGTG GCGGACGGGT GAGTAATGTC TGGG-AAACT GCCCGATGGA GGGGGATAAC TACTGGAAAC GGTAGCTAAT ACCGCAT--- -------AAC

PaKu21_-_Bacillus_sonorensis ---------- ---------- ------CTCA GGACGAACGC TGGCGGCGTG CTTAATACAT GCAAGTCGAG CGAACCGAC- GAGA--GCTT GCTCCC---T TAGGTTAGCG GCGGACGGGT GAGTAACACG TGGGTAACCT GCCTGTAAGA CTGGGATAAC TCCGGGAAAC CGGGGCTAAT ACCGGATGCT CGATTGAACC

Bacillus_sonorensis_(KU551167. ---------- -AGAGTTTGA TCCTGGCTCA GGACGAACGC TGGCGGCGTG CCTAATACAT GCAAGTCGAG CGAACCGAC- GGGA--GCTT GCTCCC---T TAGGTTAGCG GCGGACGGGT GAGTAACACG TGGGTAACCT GCCTGTAAGA CTGGGATAAC TCCGGGAAAC CGGGGCTAAT ACCGGATGCT TGATTGAACC

Bacillus_sonorensis_(FN397516. ---------- ---------- ---TGGCTCA GGACGAACGC TGGCGGCGTG CCTAATACAT GCAAGTCGAG CGAACCGAC- GGGA--GCTT GCTCCC---T TAGGTTAGCG GCGGACGGGT GAGTAACACG TGGGTAACCT GCCTGTAAGA CTGGGATAAC TCCGGGAAAC CGGGGCTAAT ACCGGATGCT TGATTGAACC

Bacillus_sonorensis_(KU551137. ---------- -AGAGTTTGA TCCTGGCTCA GGACGAACGC TGGCGGCGTG CCTAATACAT GCAAGTCGAG CGAACCGAC- GGGA--GCTT GCTCCC---T TAGGTTAGCG GCGGACGGGT GAGTAACACG TGGGTAACCT GCCTGTAAGA CTGGGATAAC TCCGGGAAAC CGGGGCTAAT ACCGGATGCT TGATTGAACC

PaKu24_-_Bacillus_sonorensis ---------- ---------- -----GCTCA GGACGAACGC GGGCGGCGTG CTTAATACAT GCAAGTCGAG CGAACCGAC- GAGA--GCTT GCTCCC---T TAGGTTAGCG GCGGACGGGT GAGTAACACG TGGGTAACCT GCCTGTAAGA CTGGGATAAC TCCGGGAAAC CGGGGCTAAT ACCGGATGCT CGATTGAACC

PaKu22_-_Bacillus_subtilis ---------- ---------- --------TC AGACGAACGC TGGCGGCGTG CCTAATACAT GCAAGTCGAG CGGACAGAT- GGGA--GCTT GCTCCC---T GATGTTAGCG GCGGACGGGT GAGTAACACG TGGGTAACCT GCCTGTAAGA CTGGGATAAC TCCGGGAAAC CGGGGCTAAT ACCGGATGGT TGTTTGAACC

Bacillus_tequilensis_(MK880583 TTTTTTTTTT TTTTTTTTTT TTATTCTGTC AGACGAACGC TGGCGGCGTG CCTAATACAT GCAAGTCGAG CGGACAGAT- GGGA--GCTT GCTCCC---T GATGTTAGCG GCGGACGGGT GAGTAACACG TGGGTAACCT GCCTGTAAGA CTGGGATAAC TCCGGGAAAC CGGGGCTAAT ACCGGATGGT TGTTTGAACC

Bacillus_subtilis_(MK765023.1) ---------- ---------- --------TC AGACGAACGC TGGCGGCGTG CCTAATACAT GCAAGTCGAG CGGACAGAT- GGGA--GCTT GCTCCC---T GATGTTAGCG GCGGACGGGT GAGTAACACG TGGGTAACCT GCCTGTAAGA CTGGGATAAC TCCGGGAAAC CGGGGCTAAT ACCGGATGGT TGTTTGAACC

PaKu23_-_Bacillus_subtilis ---------- ---------- -------GTC AGACGAACGC TGGCGGCGTG CCTAATACAT GCAAGTCGAG CGGACAGAT- GGGA--GCTT GCTCCC---T GATGTTAGCG GCGGACGGGT GAGTAACACG TGGGTAACCT GCCTGTAAGA CTGGGATAAC TCCGGGAAAC CGGGGCTAAT ACCGGATGGT TGTTTGAACC

Bacillus_subtilis_(MK511833.1) ---TTTATAT ATATGTTTTT TTTTTCTGTC AGACGAACGC TGGCGGCGTG CCTAATACAT GCAAGTCGAG CGGACAGAT- GGGA--GCTT GCTCCC---T GATGTTAGCG GCGGACGGGT GAGTAACACG TGGGTAACCT GCCTGTAAGA CTGGGATAAC TCCGGGAAAC CGGGGCTAAT ACCGGATGGT TGTTTGAACC

Bacillus_subtilis_(KX450400.1) ---TTTATAT ATATGTTTTT TTTTTCTGTC AGACGAACGC TGGCGGCGTG CCTAATACAT GCAAGTCGAG CGGACAGAT- GGGA--GCTT GCTCCC---T GATGTTAGCG GCGGACGGGT GAGTAACACG TGGGTAACCT GCCTGTAAGA CTGGGATAAC TCCGGGAAAC CGGGGCTAAT ACCGGATGGT TGTTTGAACC

PaKu25_-_Bacillus_cereus ---------- ---------- -----GCTCA GGATGAACGC TGGCGGCGTG CCTAATACAT GCAAGTCGAG CGAATGGATT GAGA--GCTT GCTCTCA--A GAAGTTAGCG GCGGACGGGT GAGTAACACG TGGGTAACCT GCCCATAAGA CTGGGATAAC TCCGGGAAAC CGGGGCTAAT ACCGGATAAC ATTTTGAACT

Bacillus_thuringiensis_(CP0378 ---------- ---------- ---------A GGATGAACGC TGGCGGCGTG CCTAATACAT GCAAGTCGAG CGAATGGATT GAGA--GCTT GCTCTCA--A GAAGTTAGCG GCGGACGGGT GAGTAACACG TGGGTAACCT GCCCATAAGA CTGGGATAAC TCCGGGAAAC CGGGGCTAAT ACCGGATAAC ATTTTGAACT

Bacillus_mobilis_(CP031443.1) ---------- ---------- ---------A GGATGAACGC TGGCGGCGTG CCTAATACAT GCAAGTCGAG CGAATGGATT GAGA--GCTT GCTCTCA--A GAAGTTAGCG GCGGACGGGT GAGTAACACG TGGGTAACCT GCCCATAAGA CTGGGATAAC TCCGGGAAAC CGGGGCTAAT ACCGGATAAC ATTTTGAACT

Bacillus_thuringiensis_(CP0357 ---------- ---------- ---------A GGATGAACGC TGGCGGCGTG CCTAATACAT GCAAGTCGAG CGAATGGATT GAGA--GCTT GCTCTCA--A GAAGTTAGCG GCGGACGGGT GAGTAACACG TGGGTAACCT GCCCATAAGA CTGGGATAAC TCCGGGAAAC CGGGGCTAAT ACCGGATAAC ATTTTGAACT

Bacillus_cereus_(CP030982.1) ---------- ---------- ---------A GGATGAACGC TGGCGGCGTG CCTAATACAT GCAAGTCGAG CGAATGGATT GAGA--GCTT GCTCTCA--A GAAGTTAGCG GCGGACGGGT GAGTAACACG TGGGTAACCT GCCCATAAGA CTGGGATAAC TCCGGGAAAC CGGGGCTAAT ACCGGATAAC ATTTTGAACT

Bacillus_cereus_(MH068823.1) ---------- -AGAGTTTGA TCCTGGCTCA GGATGAACGC TGGCGGCGTG CCTAATACAT GCAAGTCGAG CGAATGGATT GAGA--GCTT GCTCTCA--A GAAGTTAGCG GCGGACGGGT GAGTAACACG TGGGTAACCT GCCCATAAGA CTGGGATAAC TCCGGGAAAC CGGGGCTAAT ACCGGATAAC ATTTTGAACT

Bacillus_thuringiensis_(MG7453 ---------- ---------- ---------A GGATGAACGC TGGCGGCGTG CCTAATACAT GCAAGTCGAG CGAATGGATT GAGA--GCTT GCTCTCA--A GAAGTTAGCG GCGGACGGGT GAGTAACACG TGGGTAACCT GCCCATAAGA CTGGGATAAC TCCGGGAAAC CGGGGCTAAT ACCGGATAAC ATTTTGAACT

Rhizobium_massiliae(Country-_T ---------- ---------- ---------- ---------- ---------- ---------- ---------- ---------- ---------- ---------- ---------- ---------- ---------- ---------- ---------- ---------- ---------- ---------- ---------- ----------

Stenotrophomonas_maltophilia_( ---------- ---------- ---------- ---------- ---------- ---------- ---------- ---------- ---------- ---------- ---------- ---------- ---------- ---------- ---------- ---------- ---------- ---------- ---------- ----------

Serratia_sp._(Country-_Tunisia ---------- ---------- ---------- ---------- ---------- ---------- ---------- ---------- ---------- ---------- ---------- ---------- ---------- ---------- ---------- ---------- ---------- ---------- ---------- ----------

Staphylococcus_saprophyticus_( ---------- ---------- ---------- ---------- ---------- ---------- ---------- ---------- ---------- ---------- ---------- ---------- ---------- ---------- ---------- ---------- ---------- ---------- ---------- ----------

Staphylococcus_saprophyticus_( ---------- ---------- ---------- ---------- ---------- ---------- ---------- ---------- ---------- ---------- ---------- ---------- ---------- ---------- ---------- ---------- ---------- ---------- ---------- ----------

Bacillus_subtilis_(Country-Ira ---------- ---------- ---------- ---------- ---------- ---------- ---------- ---------- ---------- ---------- ---------- ---------- ---------- ---------- ---------- ---------- ---------- ---------- ---------- ----------

Bacillus_cereus_(Country-UK)_( ---------- ---------- ---------- ---------- ---------- ---------- -------GAG CGA-TGGATT -AGA--GCTT GCTCTTA--T GAAGTTAGCG GCGGACGGGT GAGTAACACG TGGGTAACCT GCCCATAAGA CTGGGATAAC TCCGGGAAAC CGGGGCTAAT ACCGGATAAC ATTTTGAACT

....|....| ....|....| ....|....| ....|....| ....|....| ....|....| ....|....| ....|....| ....|....| ....|....| ....|....| ....|....| ....|....| ....|....| ....|....| ....|....| ....|....| ....|....| ....|....| ....|....|

205 215 225 235 245 255 265 275 285 295 305 315 325 335 345 355 365 375 385 395

PaKu1_-_Serratia_marcescens GTCGCAAGAC CAAAGAGGGG GACCTTCGGG CCTCTTGCCA TCAGATGTGC CCAGATGGGA TTAGCTAGTA GGTGGGGTAA TGGCTCACCT AGGCGACGAT CCCTAGCTGG TCTGAGAGGA TGACCAGCCA CACTGGAACT GAGACACGGT CCAGACTCCT ACGGGAGGCA GCAGTGGGGA ATATTGCACA ATGGGCGCAA

Serratia_marcescens_(KX911721. GTCGCAAGAC CAAAGAGGGG GACCTTCGGG CCTCTTGCCA TCAGATGTGC CCAGATGGGA TTAGCTAGTA GGTGGGGTAA TGGCTCACCT AGGCGACGAT CCCTAGCTGG TCTGAGAGGA TGACCAGCCA CACTGGAACT GAGACACGGT CCAGACTCCT ACGGGAGGCA GCAGTGGGGA ATATTGCACA ATGGGCGCAA

Serratia_marcescens_(KT992361. GTCGCAAGAC CAAAGAGGGG GACCTTCGGG CCTCTTGCCA TCAGATGTGC CCAGATGGGA TTAGCTAGTA GGTGGGGTAA TGGCTCACCT AGGCGACGAT CCCTAGCTGG TCTGAGAGGA TGACCAGCCA CACTGGAACT GAGACACGGT CCAGACTCCT ACGGGAGGCA GCAGTGGGGA ATATTGCACA ATGGGCGCAA

PaKu2_-_Enterobacter_sp. GTCGCAAGAC CAAAGAGGGG GACCTTCGGG CCTCTTGCCA TCGGATGTGC CCAGATGGGA TTAGCTAGTA GGTGGGGTAA CGGCTCACCT AGGCGACGAT CCCTAGCTGG TCTGAGAGGA TGACCAGCCA CACTGGAACT GAGACACGGT CCAGACTCCT ACGGGAGGCA GCAGTGGGGA ATATTGCACA ATGGGCGCAA

Enterobacter_sp._(MG280962.1) GTCGCAAGAC CAAAGAGGGG GACCTTCGGG CCTCTTGCCA TCGGATGTGC CCAGATGGGA TTAGCTAGTA GGTGGGGTAA CGGCTCACCT AGGCGACGAT CCCTAGCTGG TCTGAGAGGA TGACCAGCCA CACTGGAACT GAGACACGGT CCAGACTCCT ACGGGAGGCA GCAGTGGGGA ATATTGCACA ATGGGCGCAA

Enterobacter_hormaechei_(CP029 GTCGCAAGAC CAAAGAGGGG GACCTTCGGG CCTCTTGCCA TCGGATGTGC CCAGATGGGA TTAGCTAGTA GGTGGGGTAA CGGCTCACCT AGGCGACGAT CCCTAGCTGG TCTGAGAGGA TGACCAGCCA CACTGGAACT GAGACACGGT CCAGACTCCT ACGGGAGGCA GCAGTGGGGA ATATTGCACA ATGGGCGCAA

PaKu3_-_Staphylococcus_saproph GCATGGTTCT AAAGTGAAAG ATGGTTTT-G -CTATCACTT ATAGATGGAC CCGCGCCGTA TTAGCTAGTT GGTAAGGTAA CGGCTTACCA AGGCGACGAT ACGTAGCCGA CCTGAGAGGG TGATCGGCCA CACTGGAACT GAGACACGGT CCAGACTCCT ACGGGAGGCA GCAGTAGGGA ATCTTCCGCA ATGGGCGAAA

Staphylococcus_saprophyticus_( GCATGGTTCT AAAGTGAAAG ATGGTTTT-G -CTATCACTT ATAGATGGAC CCGCGCCGTA TTAGCTAGTT GGTAAGGTAA CGGCTTACCA AGGCGACGAT ACGTAGCCGA CCTGAGAGGG TGATCGGCCA CACTGGAACT GAGACACGGT CCAGACTCCT ACGGGAGGCA GCAGTAGGGA ATCTTCCGCA ATGGGCGAAA

Staphylococcus_saprophyticus_( GCATGGTTCT AAAGTGAAAG ATGGTTTT-G -CTATCACTT ATAGATGGAC CCGCGCCGTA TTAGCTAGTT GGTAAGGTAA CGGCTTACCA AGGCGACGAT ACGTAGCCGA CCTGAGAGGG TGATCGGCCA CACTGGAACT GAGACACGGT CCAGACTCCT ACGGGAGGCA GCAGTAGGGA ATCTTCCGCA ATGGGCGAAA

PaKu4_-_Enterobacter_cloacae GTCGCAAGAC CAAAGAGGGG GACCTTCGGG CCTCTTGCCA TCAGATGTGC CCAGATGGGA TTAGCTAGTA GGTGGGGTAA CGGCTCACCT AGGCGACGAT CCCTAGCTGG TCTGAGAGGA TGACCAGCCA CACTGGAACT GAGACACGGT CCAGACTCCT ACGGGAGGCA GCAGTGGGGA ATATTGCACA ATGGGCGCAA

Uncultured_Enterobacter_sp._(M GTCGCAAGAC CAAAGAGGGG GACCTTCGGG CCTCTTGCCA TCAGATGTGC CCAGATGGGA TTAGCTAGTA GGTGGGGTAA CGGCTCACCT AGGCGACGAT CCCTAGCTGG TCTGAGAGGA TGACCAGCCA CACTGGAACT GAGACACGGT CCAGACTCCT ACGGGAGGCA GCAGTGGGGA ATATTGCACA ATGGGCGCAA

Enterobacter_cloacae_(KY930709 GTCGCAAGAC CAAAGAGGGG GACCTTCGGG CCTCTTGCCA TCAGATGTGC CCAGATGGGA TTAGCTAGTA GGTGGGGTAA CGGCTCACCT AGGCGACGAT CCCTAGCTGG TCTGAGAGGA TGACCAGCCA CACTGGAACT GAGACACGGT CCAGACTCCT ACGGGAGGCA GCAGTGGGGA ATATTGCACA ATGGGCGCAA

PaKu5_-_Enterobacter_cloacae GTCGCAAGAC CAAAGAGGGG GACCTTCGGG CCTCTTGCCA TCAGATGTGC CCAGATGGGA TTAGCTAGTA GGTGGGGTAA CGGCTCACCT AGGCGACGAT CCCTAGCTGG TCTGAGAGGA TGACCAGCCA CACTGGAACT GAGACACGGT CCAGACTCCT ACGGGAGGCA GCAGTGGGGA ATATTGCACA ATGGGCGCAA

Enterobacter_sp._(KU867838.1) GTCGCAAGAC CAAAGAGGGG GACCTTCGGG CCTCTTGCCA TCAGATGTGC CCAGATGGGA TTAGCTAGTA GGTGGGGTAA CGGCTCACCT AGGCGACGAT CCCTAGCTGG TCTGAGAGGA TGACCAGCCA CACTGGAACT GAGACACGGT CCAGACTCCT ACGGGAGGCA GCAGTGGGGA ATATTGCACA ATGGGCGCAA

Enterobacter_cloacae_(KX242265 GTCGCAAGAC CAAAGAGGGG GACCTTCGGG CCTCTTGCCA TCAGATGTGC CCAGATGGGA TTAGCTAGTA GGTGGGGTAA CGGCTCACCT AGGCGACGAT CCCTAGCTGG TCTGAGAGGA TGACCAGCCA CACTGGAACT GAGACACGGT CCAGACTCCT ACGGGAGGCA GCAGTGGGGA ATATTGCACA ATGGGCGCAA

Enterobacter_cloacae_(KP794925 GTCGCAAGAC CAAAGAGGGG GACCTTCGGG CCTCTTGCCA TCAGATGTGC CCAGATGGGA TTAGCTAGTA GGTGGGGTAA CGGCTCACCT AGGCGACGAT CCCTAGCTGG TCTGAGAGGA TGACCAGCCA CACTGGAACT GAGACACGGT CCAGACTCCT ACGGGAGGCA GCAGTGGGGA ATATTGCACA ATGGGCGCAA

PaKu6_-_Staphylococcus_sciuri GCATGGTTCA ATAGTGAAAG ACGGTTTC-G GCTGTCACTT ATAGATGGAC CCGCGCCGTA TTAGCTAGTT GGTAAGGTAA CGGCTTACCA AGGCGACGAT ACGTAGCCGA CCTGAGAGGG TGATCGGCCA CACTGGAACT GAGACACGGT CCAGACTCCT ACGGGAGGCA GCAGTAGGGA ATCTTCCGCA ATGGGCGAAA

Staphylococcus_sciuri_(MG70600 GCATGGTTCA ATAGTGAAAG ACGGTTTC-G GCTGTCACTT ATAGATGGAC CCGCGCCGTA TTAGCTAGTT GGTAAGGTAA CGGCTTACCA AGGCGACGAT ACGTAGCCGA CCTGAGAGGG TGATCGGCCA CACTGGAACT GAGACACGGT CCAGACTCCT ACGGGAGGCA GCAGTAGGGA ATCTTCCGCA ATGGGCGAAA

Staphylococcus_lentus_(MG98829 GCATGGTTCA ATAGTGAAAG ACGGTTTC-G GCTGTCACTT ATAGATGGAC CCGCGCCGTA TTAGCTAGTT GGTAAGGTAA CGGCTTACCA AGGCGACGAT ACGTAGCCGA CCTGAGAGGG TGATCGGCCA CACTGGAACT GAGACACGGT CCAGACTCCT ACGGGAGGCA GCAGTAGGGA ATCTTCCGCA ATGGGCGAAA

Staphylococcus_sciuri_(MG51742 GCATGGTTCA ATAGTGAAAG ACGGTTTC-G GCTGTCACTT ATAGATGGAC CCGCGCCGTA TTAGCTAGTT GGTAAGGTAA CGGCTTACCA AGGCGACGAT ACGTAGCCGA CCTGAGAGGG TGATCGGCCA CACTGGAACT GAGACACGGT CCAGACTCCT ACGGGAGGCA GCAGTAGGGA ATCTTCCGCA ATGGGCGAAA

PaKu8_-_Pseudomonas_aeruginosa TCCTGAGGGA GAAAGTGGGG GATCTTCGGA CCTCACGCTA TCAGATGAGC CTAGGTCGGA TTAGCTAGTT GGTGGGGTAA AGGCCTACCA AGGCGACGAT CCGTAACTGG TCTGAGAGGA TGATCAGTCA CACTGGAACT GAGACACGGT CCAGACTCCT ACGGGAGGCA GCAGTGGGGA ATATTGGACA ATGGGCGAAA

Uncultured_bacterium_(FM996243 TCCTGAGGGA GAAAGTGGGG GATCTTCGGA CCTCACGCTA TCAGATGAGC CTAGGTCGGA TTAGCTAGTT GGTGGGGTAA AGGCCTACCA AGGCGACGAT CCGTAACTGG TCTGAGAGGA TGATCAGTCA CACTGGAACT GAGACACGGT CCAGACTCCT ACGGGAGGCA GCAGTGGGGA ATATTGGACA ATGGGCGAAA

Pseudomonas_aeruginosa_(MK4304 TCCTGAGGGA GAAAGTGGGG GATCTTCGGA CCTCACGCTA TCAGATGAGC CTAGGTCGGA TTAGCTAGTT GGTGGGGTAA AGGCCTACCA AGGCGACGAT CCGTAACTGG TCTGAGAGGA TGATCAGTCA CACTGGAACT GAGACACGGT CCAGACTCCT ACGGGAGGCA GCAGTGGGGA ATATTGGACA ATGGGCGAAA

PaKu9_-_Staphylococcus_arletta GCATGGTTCT AAAGTGAAAG ATGGTTTT-G -CTATCACTT ATAGATGGAC CCGCGCCGTA TTAGCTAGTT GGTAAGGTAA TGGCTTACCA AGGCAACGAT ACGTAGCCGA CCTGAGAGGG TGATCGGCCA CACTGGAACT GAGACACGGT CCAGACTCCT ACGGGAGGCA GCAGTAGGGA ATCTTCCGCA ATGGGCGAAA

Staphylococcus_arlettae_(KX344 GCATGGTTCT AAAGTGAAAG ATGGTTTT-G -CTATCACTT ATAGATGGAC CCGCGCCGTA TTAGCTAGTT GGTAAGGTAA TGGCTTACCA AGGCAACGAT ACGTAGCCGA CCTGAGAGGG TGATCGGCCA CACTGGAACT GAGACACGGT CCAGACTCCT ACGGGAGGCA GCAGTAGGGA ATCTTCCGCA ATGGGCGAAA

Staphylococcus_sp._(KU891836.1 GCATGGTTCT AAAGTGAAAG ATGGTTTT-G -CTATCACTT ATAGATGGAC CCGCGCCGTA TTAGCTAGTT GGTAAGGTAA TGGCTTACCA AGGCAACGAT ACGTAGCCGA CCTGAGAGGG TGATCGGCCA CACTGGAACT GAGACACGGT CCAGACTCCT ACGGGAGGCA GCAGTAGGGA ATCTTCCGCA ATGGGCGAAA

PaKu10_-_Aeromonas_caviae CCCTACGGGG GAAAGCAGGG GACCTTCGGG CCTTGCGCGA TTGGATATGC CCAGGTGGGA TTAGCTAGTT GGTGAGGTAA TGGCTCACCA AGGCGACGAT CCCTAGCTGG TCTGAGAGGA TGATCAGCCA CACTGGAACT GAGACACGGT CCAGACTCCT ACGGGAGGCA GCAGTGGGGA ATATTGCACA ATGGGGGAAA

Uncultured_bacterium_(KX242397 CCCTACGGGG GAAAGCAGGG GACCTTCGGG CCTTGCGCGA TTGGATATGC CCAGGTGGGA TTAGCTAGTT GGTGAGGTAA TGGCTCACCA AGGCGACGAT CCCTAGCTGG TCTGAGAGGA TGATCAGCCA CACTGGAACT GAGACACGGT CCAGACTCCT ACGGGAGGCA GCAGTGGGGA ATATTGCACA ATGGGGGAAA

Aeromonas_caviae_(HQ407268.1) CCCTACGGGG GAAAGCAGGG GACCTTCGGG CCTTGCGCGA TTGGATATGC CCAGGTGGGA TTAGCTAGTT GGTGAGGTAA TGGCTCACCA AGGCGACGAT CCCTAGCTGG TCTGAGAGGA TGATCAGCCA CACTGGAACT GAGACACGGT CCAGACTCCT ACGGGAGGCA GCAGTGGGGA ATATTGCACA ATGGGGGAAA

Aeromonas_aquariorum_(EU085557 CCCTACGGGG GAAAGCAGGG GACCTTCGGG CCTTGCGCGA TTGGATATGC CCAGGTGGGA TTAGCTAGTT GGTGAGGTAA TGGCTCACCA AGGCGACGAT CCCTAGCTGG TCTGAGAGGA TGATCAGCCA CACTGGAACT GAGACACGGT CCAGACTCCT ACGGGAGGCA GCAGTGGGGA ATATTGCACA ATGGGGGAAA

PaKu11_-_Aeromonas_caviae CCCTACGGGG GAAAGCAGGG GACCTTCGGG CCTTGCGCGA TTGGATATGC CCAGGTGGGA TTAGCTAGTT GGTGAGGTAA TGGCTCACCA AGGCGACGAT CCCTAGCTGG TCTGAGAGGA TGATCAGCCA CACTGGAACT GAGACACGGT CCAGACTCCT ACGGGAGGCA GCAGTGGGGA ATATTGCACA ATGGGGGAAA

Aeromonas_caviae_(KY087979.1) CCCTACGGGG GAAAGCAGGG GACCTTCGGG CCTTGCGCGA TTGGATATGC CCAGGTGGGA TTAGCTAGTT GGTGAGGTAA TGGCTCACCA AGGCGACGAT CCCTAGCTGG TCTGAGAGGA TGATCAGCCA CACTGGAACT GAGACACGGT CCAGACTCCT ACGGGAGGCA GCAGTGGGGA ATATTGCACA ATGGGGGAAA

Uncultured_Aeromonas_sp._(KU94 CCCTACGGGG GAAAGCAGGG GACCTTCGGG CCTTGCGCGA TTGGATATGC CCAGGTGGGA TTAGCTAGTT GGTGAGGTAA TGGCTCACCA AGGCGACGAT CCCTAGCTGG TCTGAGAGGA TGATCAGCCA CACTGGAACT GAGACACGGT CCAGACTCCT ACGGGAGGCA GCAGTGGGGA ATATTGCACA ATGGGGGAAA

PaKu12_-_Staphylococcus_warner GCATGGTTCA ATAGTGAAAG GCGGCTTT-G -CTGTCACTT ATAGATGGAT CCGCGCCGTA TTAGCTAGTT GGTAAGGTAA CGGCTTACCA AGGCAACGAT ACGTAGCCGA CCTGAGAGGG TGATCGGCCA CACTGGAACT GAGACACGGT CCAGACTCCT ACGGGAGGCA GCAGTAGGGA ATCTTCCGCA ATGGGCGAAA

Staphylococcus_warneri_(MH0149 GCATGGTTCA ATAGTGAAAG GCGGCTTT-G -CTGTCACTT ATAGATGGAT CCGCGCCGTA TTAGCTAGTT GGTAAGGTAA CGGCTTACCA AGGCAACGAT ACGTAGCCGA CCTGAGAGGG TGATCGGCCA CACTGGAACT GAGACACGGT CCAGACTCCT ACGGGAGGCA GCAGTAGGGA ATCTTCCGCA ATGGGCGAAA

Staphylococcus_warneri_(KF3063 GCATGGTTCA ATAGTGAAAG GCGGCTTT-G -CTGTCACTT ATAGATGGAT CCGCGCCGTA TTAGCTAGTT GGTAAGGTAA CGGCTTACCA AGGCAACGAT ACGTAGCCGA CCTGAGAGGG TGATCGGCCA CACTGGAACT GAGACACGGT CCAGACTCCT ACGGGAGGCA GCAGTAGGGA ATCTTCCGCA ATGGGCGAAA

PaKu13_-_Bacillus_megaterium TCATGGGAGA TGATTGAAAG ATGGTTTC-G GCTATCACTT ACAGATGGGC CCGCGGTGCA TTAGCTAGTT GGTGAGGTAA CGGCTCACCA AGGCAACGAT GCATAGCCGA CCTGAGAGGG TGATCGGCCA CACTGGGACT GAGACACGGC CCAGACTCCT ACGGGAGGCA GCAGTAGGGA ATCTTCCGCA ATGGACGAAA

Bacillus_sp._(MK691444.1) TCATGGGAGA TGATTGAAAG ATGGTTTC-G GCTATCACTT ACAGATGGGC CCGCGGTGCA TTAGCTAGTT GGTGAGGTAA CGGCTCACCA AGGCAACGAT GCATAGCCGA CCTGAGAGGG TGATCGGCCA CACTGGGACT GAGACACGGC CCAGACTCCT ACGGGAGGCA GCAGTAGGGA ATCTTCCGCA ATGGACGAAA

Bacillus_sp._(LC373523.1) TCATGGGAGA TGATTGAAAG ATGGTTTC-G GCTATCACTT ACAGATGGGC CCGCGGTGCA TTAGCTAGTT GGTGAGGTAA CGGCTCACCA AGGCAACGAT GCATAGCCGA CCTGAGAGGG TGATCGGCCA CACTGGGACT GAGACACGGC CCAGACTCCT ACGGGAGGCA GCAGTAGGGA ATCTTCCGCA ATGGACGAAA

Bacillus_aryabhattai_(MK474942 TCATGGGAGA TGATTGAAAG ATGGTTTC-G GCTATCACTT ACAGATGGGC CCGCGGTGCA TTAGCTAGTT GGTGAGGTAA CGGCTCACCA AGGCAACGAT GCATAGCCGA CCTGAGAGGG TGATCGGCCA CACTGGGACT GAGACACGGC CCAGACTCCT ACGGGAGGCA GCAGTAGGGA ATCTTCCGCA ATGGACGAAA

Bacillus_aryabhattai_(MK474941 TCATGGGAGA TGATTGAAAG ATGGTTTC-G GCTATCACTT ACAGATGGGC CCGCGGTGCA TTAGCTAGTT GGTGAGGTAA CGGCTCACCA AGGCAACGAT GCATAGCCGA CCTGAGAGGG TGATCGGCCA CACTGGGACT GAGACACGGC CCAGACTCCT ACGGGAGGCA GCAGTAGGGA ATCTTCCGCA ATGGACGAAA

Bacillus_megaterium_(KX298860. TCATGGGAGA TGATTGAAAG ATGGTTTC-G GCTATCACTT ACAGATGGGC CCGCGGTGCA TTAGCTAGTT GGTGAGGTAA CGGCTCACCA AGGCAACGAT GCATAGCCGA CCTGAGAGGG TGATCGGCCA CACTGGGACT GAGACACGGC CCAGACTCCT ACGGGAGGCA GCAGTAGGGA ATCTTCCGCA ATGGACGAAA

PaKu14_-_Pseudomonas_stutzeri TCCTACGGGA GAAAGTGGGG GATCTTCGGA CCTCACGCTA TCAGATGAGC CTAGGTCGGA TTAGCTAGTT GGTGAGGTAA AGGCTCACCA AGGCGACGAT CCGTAACTGG TCTGAGAGGA TGATCAGTCA CACTGGAACT GAGACACGGT CCAGACTCCT ACGGGAGGCA GCAGTGGGGA ATATTGGACA ATGGGCGAAA

Pseudomonas_stutzeri_(MG892782 TCCTACGGGA GAAAGTGGGG GATCTTCGGA CCTCACGCTA TCAGATGAGC CTAGGTCGGA TTAGCTAGTT GGTGAGGTAA AGGCTCACCA AGGCGACGAT CCGTAACTGG TCTGAGAGGA TGATCAGTCA CACTGGAACT GAGACACGGT CCAGACTCCT ACGGGAGGCA GCAGTGGGGA ATATTGGACA ATGGGCGAAA

Pseudomonas_stutzeri_(MG595371 TCCTACGGGA GAAAGTGGGG GATCTTCGGA CCTCACGCTA TCAGATGAGC CTAGGTCGGA TTAGCTAGTT GGTGAGGTAA AGGCTCACCA AGGCGACGAT CCGTAACTGG TCTGAGAGGA TGATCAGTCA CACTGGAACT GAGACACGGT CCAGACTCCT ACGGGAGGCA GCAGTGGGGA ATATTGGACA ATGGGCGAAA

PaKu15_-_Rhizobium_sp. --ACGCCCTA CGGGGGAAAG AT-------- -TTATCGGGG AAGGATTGGC CCGCGTTGGA TTAGCTAGTT GGTGGGGTAA AGGCCTACCA AGGCGACGAT CCATAGCTGG TCTGAGAGGA TGATCAGCCA CATTGGGACT GAGACACGGC CCAAACTCCT ACGGGAGGCA GCAGTGGGGA ATATTGGACA ATGGGCGCAA

Rhizobium_pusense_(CP039895.1) --ACGCCCTA CGGGGGAAAG AT-------- -TTATCGGGG AAGGATTGGC CCGCGTTGGA TTAGCTAGTT GGTGGGGTAA AGGCCTACCA AGGCGACGAT CCATAGCTGG TCTGAGAGGA TGATCAGCCA CATTGGGACT GAGACACGGC CCAAACTCCT ACGGGAGGCA GCAGTGGGGA ATATTGGACA ATGGGCGCAA

Rhizobium_sp._(KY971009.1) --ACGCCCTA CGGGGGAAAG AT-------- -TTATCGGGG AAGGATTGGC CCGCGTTGGA TTAGCTAGTT GGTGGGGTAA AGGCCTACCA AGGCGACGAT CCATAGCTGG TCTGAGAGGA TGATCAGCCA CATTGGGACT GAGACACGGC CCAAACTCCT ACGGGAGGCA GCAGTGGGGA ATATTGGACA ATGGGCGCAA

PaKu7_-_Stenotrophomonas_malto ACCTACGGGT GAAAGCAGGG GACCTTCGGG CCTTGCGCGA TTGAATGAGC CGATGTCGGA TTAGCTAGTT GGCGGGGTAA AGGCCCACCA AGGCGACGAT CCGTAGCTGG TCTGAGAGGA TGATCAGCCA CACTGGAACT GAGACACGGT CCAGACTCCT ACGGGAGGCA GCAGTGGGGA ATATTGGACA ATGGGCGCAA

Stenotrophomonas_maltophilia_( ACCTACGGGT GAAAGCAGGG GACCTTCGGG CCTTGCGCGA TTGAATGAGC CGATGTCGGA TTAGCTAGTT GGCGGGGTAA AGGCCCACCA AGGCGACGAT CCGTAGCTGG TCTGAGAGGA TGATCAGCCA CACTGGAACT GAGACACGGT CCAGACTCCT ACGGGAGGCA GCAGTGGGGA ATATTGGACA ATGGGCGCAA

Stenotrophomonas_maltophilia_( ACCTACGGGT GAAAGCAGGG GACCTTCGGG CCTTGCGCGA TTGAATGAGC CGATGTCGGA TTAGCTAGTT GGCGGGGTAA AGGCCCACCA AGGCGACGAT CCGTAGCTGG TCTGAGAGGA TGATCAGCCA CACTGGAACT GAGACACGGT CCAGACTCCT ACGGGAGGCA GCAGTGGGGA ATATTGGACA ATGGGCGCAA

PaKu16_-_Stenotrophomonas_malt ACCTACGGGT GAAAGCAGGG GACCTTCGGG CCTTGCGCGA TTGAATGAGC CGATGTCGGA TTAGCTAGTT GGCGGGGTAA AGGCCCACCA AGGCGACGAT CCGTAGCTGG TCTGAGAGGA TGATCAGCCA CACTGGAACT GAGACACGGT CCAGACTCCT ACGGGAGGCA GCAGTGGGGA ATATTGGACA ATGGGCGCAA

Stenotrophomonas_maltophilia_( ACCTACGGGT GAAAGCAGGG GACCTTCGGG CCTTGCGCGA TTGAATGAGC CGATGTCGGA TTAGCTAGTT GGCGGGGTAA AGGCCCACCA AGGCGACGAT CCGTAGCTGG TCTGAGAGGA TGATCAGCCA CACTGGAACT GAGACACGGT CCAGACTCCT ACGGGAGGCA GCAGTGGGGA ATATTGGACA ATGGGCGCAA

Stenotrophomonas_maltophilia_( ACCTACGGGT GAAAGCAGGG GACCTTCGGG CCTTGCGCGA TTGAATGAGC CGATGTCGGA TTAGCTAGTT GGCGGGGTAA AGGCCCACCA AGGCGACGAT CCGTAGCTGG TCTGAGAGGA TGATCAGCCA CACTGGAACT GAGACACGGT CCAGACTCCT ACGGGAGGCA GCAGTGGGGA ATATTGGACA ATGGGCGCAA

PaKu17_-_Stenotrophomonas_malt ACCTACGGGT GAAAGCAGGG GACCTTCGGG CCTTGCGCGA TTGAATGAGC CGATGTCGGA TTAGCTAGTT GGCGGGGTAA AGGCCCACCA AGGCGACGAT CCGTAGCTGG TCTGAGAGGA TGATCAGCCA CACTGGAACT GAGACACGGT CCAGACTCCT ACGGGAGGCA GCAGTGGGGA ATATTGGACA ATGGGCGCAA

PaKu26_-_Stenotrophomonas_malt ACCTACGGGT GAAAGCAGGG GACCTTCGGG CCTTGCGCGA TTGAATGAGC CGATGTCGGA TTAGCTAGTT GGCGGGGTAA AGGCCCACCA AGGCGACGAT CCGTAGCTGG TCTGAGAGGA TGATCAGCCA CACTGGAACT GAGACACGGT CCAGACTCCT ACGGGAGGCA GCAGTGGGGA ATATTGGACA ATGGGCGCAA

Stenotrophomonas_maltophilia_( ACCTACGGGT GAAAGCAGGG GACCTTCGGG CCTTGCGCGA TTGAATGAGC CGATGTCGGA TTAGCTAGTT GGCGGGGTAA AGGCCCACCA AGGCGACGAT CCGTAGCTGG TCTGAGAGGA TGATCAGCCA CACTGGAACT GAGACACGGT CCAGACTCCT ACGGGAGGCA GCAGTGGGGA ATATTGGACA ATGGGCGCAA

PaKu18_-_Stenotrophomonas_pana ACCTTCGGGT GAAAGCAGGG GACCTTCGGG CCTTGCGCGA TTAGATGAGC CGATGTCGGA TTAGCTAGTT GGTGAGGTAA AGGCTCACCA AGGCGACGAT CCGTAGCTGG TCTGAGAGGA TGATCAGCCA CACTGGAACT GAGACACGGT CCAGACTCCT ACGGGAGGCA GCAGTGGGGA ATATTGGACA ATGGGCGCAA

Stenotrophomonas_panacihumi_(M ACCTTCGGGT GAAAGCAGGG GACCTTCGGG CCTTGCGCGA TTAGATGAGC CGATGTCGGA TTAGCTAGTT GGTGAGGTAA AGGCTCACCA AGGCGACGAT CCGTAGCTGG TCTGAGAGGA TGATCAGCCA CACTGGAACT GAGACACGGT CCAGACTCCT ACGGGAGGCA GCAGTGGGGA ATATTGGACA ATGGGCGCAA

Stenotrophomonas_panacihumi_(K ACCTTCGGGT GAAAGCAGGG GACCTTCGGG CCTTGCGCGA TTAGATGAGC CGATGTCGGA TTAGCTAGTT GGTGAGGTAA AGGCTCACCA AGGCGACGAT CCGTAGCTGG TCTGAGAGGA TGATCAGCCA CACTGGAACT GAGACACGGT CCAGACTCCT ACGGGAGGCA GCAGTGGGGA ATATTGGACA ATGGGCGCAA

PaKu19_-_Bacillus_licheniformi GCATGGTTCA ATCATAAAAG GTGGCTTTTA GCTACCACTT ACAGATGGAC CCGCGGCGCA TTAGCTAGTT GGTGAGGTAA CGGCTCACCA AGGCGACGAT GCGTAGCCGA CCTGAGAGGG TGATCGGCCA CACTGGGACT GAGACACGGC CCAGACTCCT ACGGGAGGCA GCAGTAGGGA ATCTTCCGCA ATGGACGAAA

Bacillus_licheniformis_(KJ5722 GCATGGTTCA ATCATAAAAG GTGGCTTTTA GCTACCACTT ACAGATGGAC CCGCGGCGCA TTAGCTAGTT GGTGAGGTAA CGGCTCACCA AGGCGACGAT GCGTAGCCGA CCTGAGAGGG TGATCGGCCA CACTGGGACT GAGACACGGC CCAGACTCCT ACGGGAGGCA GCAGTAGGGA ATCTTCCGCA ATGGACGAAA

Bacillus_licheniformis_(KT1536 GCATGGTTCA ATCATAAAAG GTGGCTTTTA GCTACCACTT ACAGATGGAC CCGCGGCGCA TTAGCTAGTT GGTGAGGTAA CGGCTCACCA AGGCGACGAT GCGTAGCCGA CCTGAGAGGG TGATCGGCCA CACTGGGACT GAGACACGGC CCAGACTCCT ACGGGAGGCA GCAGTAGGGA ATCTTCCGCA ATGGACGAAA

PaKu20_-__Pantoea_dispersa GTCGCAAGAC CAAAGTGGGG GACCTTCGGG CCTCACACCA TCGGATGTGC CCAGATGGGA TTAGCTAGTA GGTGGGGTAA TGGCTCACCT AGGCGACGAT CCCTAGCTGG TCTGAGAGGA TGACCAGCCA CACTGGAACT GAGACACGGT CCAGACTCCT ACGGGAGGCA GCAGTGGGGA ATATTGCACA ATGGGCGCAA

Pantoea_dispersa_(KY292463.1) GTCGCAAGAC CAAAGTGGGG GACCTTCGGG CCTCACACCA TCGGATGTGC CCAGATGGGA TTAGCTAGTA GGTGGGGTAA TGGCTCACCT AGGCGACGAT CCCTAGCTGG TCTGAGAGGA TGACCAGCCA CACTGGAACT GAGACACGGT CCAGACTCCT ACGGGAGGCA GCAGTGGGGA ATATTGCACA ATGGGCGCAA

Pantoea_dispersa_(KY882077.1) GTCGCAAGAC CAAAGTGGGG GACCTTCGGG CCTCACACCA TCGGATGTGC CCAGATGGGA TTAGCTAGTA GGTGGGGTAA TGGCTCACCT AGGCGACGAT CCCTAGCTGG TCTGAGAGGA TGACCAGCCA CACTGGAACT GAGACACGGT CCAGACTCCT ACGGGAGGCA GCAGTGGGGA ATATTGCACA ATGGGCGCAA

PaKu21_-_Bacillus_sonorensis GCATGGTTCA ATTATAAAAG GTGGCTTTTA GCTACCACTT ACAGATGGAC CCGCGGCGCA TTAGCTAGTT GGTGAGGTAA CGGCTCACCA AGGCGACGAT GCGTAGCCGA CCTGAGAGGG TGATCGGCCA CACTGGGACT GAGACACGGC CCAGACTCCT ACGGGAGGCA GCAGTAGGGA ATCTTCCGCA ATGGACGAAA

Bacillus_sonorensis_(KU551167. GCATGGTTCA ATTATAAAAG GTGGCTTTTA GCTACCACTT ACAGATGGAC CCGCGGCGCA TTAGCTAGTT GGTGAGGTAA CGGCTCACCA AGGCGACGAT GCGTAGCCGA CCTGAGAGGG TGATCGGCCA CACTGGGACT GAGACACGGC CCAGACTCCT ACGGGAGGCA GCAGTAGGGA ATCTTCCGCA ATGGACGAAA

Bacillus_sonorensis_(FN397516. GCATGGTTCA ATTATAAAAG GTGGCTTTTA GCTACCACTT ACAGATGGAC CCGCGGCGCA TTAGCTAGTT GGTGAGGTAA CGGCTCACCA AGGCGACGAT GCGTAGCCGA CCTGAGAGGG TGATCGGCCA CACTGGGACT GAGACACGGC CCAGACTCCT ACGGGAGGCA GCAGTAGGGA ATCTTCCGCA ATGGACGAAA

Bacillus_sonorensis_(KU551137. GCATGGTTCA ATTATAAAAG GTGGCTTTTA GCTATCACTT ACAGATGGAC CCGCGGCGCA TTAGCTAGTT GGTGAGGTAA CGGCTCACCA AGGCGACGAT GCGTAGCCGA CCTGAGAGGG TGATCGGCCA CACTGGGACT GAGACACGGC CCAGACTCCT ACGGGAGGCA GCAGTAGGGA ATCTTCCGCA ATGGACGAAA

PaKu24_-_Bacillus_sonorensis GCATGGTTCA ATTATAAAAG GTGGCTTTTA GCTACCACTT ACAGATGGAC CCGCGGCGCA TTAGCTAGTT GGTGAGGTAA CGGCTCACCA AGGCGACGAT GCGTAGCCGA CCTGAGAGGG TGATCGGCCA CACTGGGACT GAGACACGGC CCAGACTCCT ACGGGAGGCA GCAGTAGGGA ATCTTCCGCA ATGGACGAAA

PaKu22_-_Bacillus_subtilis GCATGGTTCA AACATAAAAG GTGGCTTC-G GCTACCACTT ACAGATGGAC CCGCGGCGCA TTAGCTAGTT GGTGAGGTAA CGGCTCACCA AGGCAACGAT GCGTAGCCGA CCTGAGAGGG TGATCGGCCA CACTGGGACT GAGACACGGC CCAGACTCCT ACGGGAGGCA GCAGTAGGGA ATCTTCCGCA ATGGACGAAA

Bacillus_tequilensis_(MK880583 GCATGGTTCA AACATAAAAG GTGGCTTC-G GCTACCACTT ACAGATGGAC CCGCGGCGCA TTAGCTAGTT GGTGAGGTAA CGGCTCACCA AGGCAACGAT GCGTAGCCGA CCTGAGAGGG TGATCGGCCA CACTGGGACT GAGACACGGC CCAGACTCCT ACGGGAGGCA GCAGTAGGGA ATCTTCCGCA ATGGACGAAA

Bacillus_subtilis_(MK765023.1) GCATGGTTCA AACATAAAAG GTGGCTTC-G GCTACCACTT ACAGATGGAC CCGCGGCGCA TTAGCTAGTT GGTGAGGTAA CGGCTCACCA AGGCAACGAT GCGTAGCCGA CCTGAGAGGG TGATCGGCCA CACTGGGACT GAGACACGGC CCAGACTCCT ACGGGAGGCA GCAGTAGGGA ATCTTCCGCA ATGGACGAAA

PaKu23_-_Bacillus_subtilis GCATGGTTCA AACATAAAAG GTGGCTTC-G GCTACCACTT ACAGATGGAC CCGCGGCGCA TTAGCTAGTT GGTGAGGTAA CGGCTCACCA AGGCAACGAT GCGTAGCCGA CCTGAGAGGG TGATCGGCCA CACTGGGACT GAGACACGGC CCAGACTCCT ACGGGAGGCA GCAGTAGGGA ATCTTCCGCA ATGGACGAAA

Bacillus_subtilis_(MK511833.1) GCATGGTTCA AACATAAAAG GTGGCTTC-G GCTACCACTT ACAGATGGAC CCGCGGCGCA TTAGCTAGTT GGTGAGGTAA CGGCTCACCA AGGCAACGAT GCGTAGCCGA CCTGAGAGGG TGATCGGCCA CACTGGGACT GAGACACGGC CCAGACTCCT ACGGGAGGCA GCAGTAGGGA ATCTTCCGCA ATGGACGAAA

Bacillus_subtilis_(KX450400.1) GCATGGTTCA AACATAAAAG GTGGCTTC-G GCTACCACTT ACAGATGGAC CCGCGGCGCA TTAGCTAGTT GGTGAGGTAA CGGCTCACCA AGGCAACGAT GCGTAGCCGA CCTGAGAGGG TGATCGGCCA CACTGGGACT GAGACACGGC CCAGACTCCT ACGGGAGGCA GCAGTAGGGA ATCTTCCGCA ATGGACGAAA

PaKu25_-_Bacillus_cereus GCATGGTTCG AAATTGAAAG GCGGCTTC-G GCTGTCACTT ATGGATGGAC CCGCGTCGCA TTAGCTAGTT GGTGAGGTAA CGGCTCACCA AGGCAACGAT GCGTAGCCGA CCTGAGAGGG TGATCGGCCA CACTGGGACT GAGACACGGC CCAGACTCCT ACGGGAGGCA GCAGTAGGGA ATCTTCCGCA ATGGACGAAA

Bacillus_thuringiensis_(CP0378 GCATGGTTCG AAATTGAAAG GCGGCTTC-G GCTGTCACTT ATGGATGGAC CCGCGTCGCA TTAGCTAGTT GGTGAGGTAA CGGCTCACCA AGGCAACGAT GCGTAGCCGA CCTGAGAGGG TGATCGGCCA CACTGGGACT GAGACACGGC CCAGACTCCT ACGGGAGGCA GCAGTAGGGA ATCTTCCGCA ATGGACGAAA

Bacillus_mobilis_(CP031443.1) GCATGGTTCG AAATTGAAAG GCGGCTTC-G GCTGTCACTT ATGGATGGAC CCGCGTCGCA TTAGCTAGTT GGTGAGGTAA CGGCTCACCA AGGCAACGAT GCGTAGCCGA CCTGAGAGGG TGATCGGCCA CACTGGGACT GAGACACGGC CCAGACTCCT ACGGGAGGCA GCAGTAGGGA ATCTTCCGCA ATGGACGAAA

Bacillus_thuringiensis_(CP0357 GCATGGTTCG AAATTGAAAG GCGGCTTC-G GCTGTCACTT ATGGATGGAC CCGCGTCGCA TTAGCTAGTT GGTGAGGTAA CGGCTCACCA AGGCAACGAT GCGTAGCCGA CCTGAGAGGG TGATCGGCCA CACTGGGACT GAGACACGGC CCAGACTCCT ACGGGAGGCA GCAGTAGGGA ATCTTCCGCA ATGGACGAAA

Bacillus_cereus_(CP030982.1) GCATGGTTCG AAATTGAAAG GCGGCTTC-G GCTGTCACTT ATGGATGGAC CCGCGTCGCA TTAGCTAGTT GGTGAGGTAA CGGCTCACCA AGGCAACGAT GCGTAGCCGA CCTGAGAGGG TGATCGGCCA CACTGGGACT GAGACACGGC CCAGACTCCT ACGGGAGGCA GCAGTAGGGA ATCTTCCGCA ATGGACGAAA

Bacillus_cereus_(MH068823.1) GCATGGTTCG AAATTGAAAG GCGGCTTC-G GCTGTCACTT ATGGATGGAC CCGCGTCGCA TTAGCTAGTT GGTGAGGTAA CGGCTCACCA AGGCAACGAT GCGTAGCCGA CCTGAGAGGG TGATCGGCCA CACTGGGACT GAGACACGGC CCAGACTCCT ACGGGAGGCA GCAGTAGGGA ATCTTCCGCA ATGGACGAAA

Bacillus_thuringiensis_(MG7453 GCATGGTTCG AAATTGAAAG GCGGCTTC-G GCTGTCACTT ATGGATGGAC CCGCGTCGCA TTAGCTAGTT GGTGAGGTAA CGGCTCACCA AGGCAACGAT GCGTAGCCGA CCTGAGAGGG TGATCGGCCA CACTGGGACT GAGACACGGC CCAGACTCCT ACGGGAGGCA GCAGTAGGGA ATCTTCCGCA ATGGACGAAA

Rhizobium_massiliae(Country-_T ---------- ---------- ---------- ---------- ---------- ---------- ---------- ---------- ---------- ---------- ---------- ---------- ---------- ---------- ---------- ---------- ---------- ---------- ---------- ----------

Stenotrophomonas_maltophilia_( ---------- ---------- ---------- ---------- ---------- ---------- ---------- ---------- ---------- ---------- ---------- ---------- ---------- ---------- ---------- ---------- ---------- ---------- ---------- ----------

Serratia_sp._(Country-_Tunisia ---------- ---------- ---------- ---------- ---------- ---------- ---------- ---------- ---------- ---------- ---------- ---------- ---------- ---------- ---------- ---------- -----AGGTA GCAGTGGGGA ACATTGTACA ATGGGCGCAA

Staphylococcus_saprophyticus_( ---------- ---------- ---------- ---------- ---------- ---------- ---------- ---------- ---------- ---------- ---------- ---------- ---------- ---------- ---------- ---------- ---------- ---------- ---------- ----------

Staphylococcus_saprophyticus_( ---------- ---------- ---------- ---------- ---------- ---------- ---------- ---------- ---------- ---------- ---------- ---------- ---------- ---------- ---------- ---------- ---------- ---------- ---------- ----------

Bacillus_subtilis_(Country-Ira ---------- ---------- ---------- ---------- ---------- ---------- ---------- ---------- ---------- ---------- ---------- ---------- ---------- ---------- ---------- ---------- ---------- ---------- ---------- ----------

Bacillus_cereus_(Country-UK)_( GCATGGTTCG AAATTGAAAG GCGGCTTC-G GCTGTCACTT ATGGATGGAC CCGCGTCGCA TTAGCTAGTT GGTGAGGTAA CGGCTCACCA AGGCAACGAT GCGTAGCCGA CCTGAGAGGG TGATCGGCCA CACTGGGACT GAGACACGGC CCAGACTCCT ACGGGAGGCA GCAGTAGGGA ATCTTCCGCA ATGGACGAAA

....|....| ....|....| ....|....| ....|....| ....|....| ....|....| ....|....| ....|....| ....|....| ....|....| ....|....| ....|....| ....|....| ....|....| ....|....| ....|....| ....|....| ....|....| ....|....| ....|....|

405 415 425 435 445 455 465 475 485 495 505 515 525 535 545 555 565 575 585 595

PaKu1_-_Serratia_marcescens GCCTGATGCA GCCATGCCGC GTGTGTGAAG AAGGCCTTCG GGTTGTAAAG CACTTTCAGC GAGGAGGAA- GGTGGTGAGC TTAATACGTT CATCAATTGA CGTTACTCGC AGAAGAAGCA CCGGCTAACT CCGTGCCAGC AGCCGCGGTA ATACGGA-GG GTGCAAGCGT TAATCGGAA- TTACTGGGCG TAAAGCGCAC

Serratia_marcescens_(KX911721. GCCTGATGCA GCCATGCCGC GTGTGTGAAG AAGGCCTTCG GGTTGTAAAG CACTTTCAGC GAGGAGGAA- GGTGGTGAAC TTAATACGTT CATCAATTGA CGTTACTCGC AGAAGAAGCA CCGGCTAACT CCGTGCCAGC AGCCGCGGTA ATACGGA-GG GTGCAAGCGT TAATCGGAA- TTACTGGGCG TAAAGCGCAC

Serratia_marcescens_(KT992361. GCCTGATGCA GCCATGCCGC GTGTGTGAAG AAGGCCTTCG GGTTGTAAAG CACTTTCAGC GAGGAGGAA- GGTGGTGAGC TTAATACGTT CATCAATTGA CGTTACTCGC AGAAGAAGCA CCGGCTAACT CCGTGCCAGC AGCCGCGGTA ATACGGA-GG GTGCAAGCGT TAATCGGAA- TTACTGGGCG TAAAGCGCAC

PaKu2_-_Enterobacter_sp. GCCTGATGCA GCCATGCCGC GTGTATGAAG AAGGCCTTCG GGTTGTAAAG TACTTTCAGC GGGGAGGAA- GGTGTTGAGG TTAATAACCT CAGCAATTGA CGTTACCCGC AGAAGAAGCA CCGGCTAACT CCGTGCCAGC AGCCGCGGTA ATACGGA-GG GTGCAAGCGT TAATCGGAA- TTACTGGGCG TAAAGCGCAC

Enterobacter_sp._(MG280962.1) GCCTGATGCA GCCATGCCGC GTGTATGAAG AAGGCCTTCG GGTTGTAAAG TACTTTCAGC GGGGAGGAA- GGTGTTGAGG TTAATAACCT CAGCAATTGA CGTTACCCGC AGAAGAAGCA CCGGCTAACT CCGTGCCAGC AGCCGCGGTA ATACGGA-GG GTGCAAGCGT TAATCGGAA- TTACTGGGCG TAAAGCGCAC

Enterobacter_hormaechei_(CP029 GCCTGATGCA GCCATGCCGC GTGTATGAAG AAGGCCTTCG GGTTGTAAAG TACTTTCAGC GGGGAGGAA- GGTGTTGAGG TTAATAACCT CAGCAATTGA CGTTACCCGC AGAAGAAGCA CCGGCTAACT CCGTGCCAGC AGCCGCGGTA ATACGGA-GG GTGCAAGCGT TAATCGGAA- TTACTGGGCG TAAAGCGCAC

PaKu3_-_Staphylococcus_saproph GCCTGACGGA GCAACGCCGC GTGAGTGATG AAGGGTTTCG GCTCGTAAAA CTCTGTTATT AGGGAAGAAC AAATGTGTAA GTAACT-GTG CACGTCTTGA CGGTACCTAA TCAGAAAGCC ACGGCTAACT ACGTGCCAGC AGCCGCGGTA ATACGTA-GG TGGCAAGCGT TATCCGGAA- TTATTGGGCG TAAAGCGCGC

Staphylococcus_saprophyticus_( GCCTGACGGA GCAACGCCGC GTGAGTGATG AAGGGTTTCG GCTCGTAAAA CTCTGTTATT AGGGAAGAAC AAATGTGTAA GTAACT-GTG CACGTCTTGA CGGTACCTAA TCAGAAAGCC ACGGCTAACT ACGTGCCAGC AGCCGCGGTA ATACGTA-GG TGGCAAGCGT TATCCGGAA- TTATTGGGCG TAAAGCGCGC

Staphylococcus_saprophyticus_( GCCTGACGGA GCAACGCCGC GTGAGTGATG AAGGGTTTCG GCTCGTAAAA CTCTGTTATT AGGGAAGAAC AAATGTGTAA GTAACT-GTG CACGTCTTGA CGGTACCTAA TCAGAAAGCC ACGGCTAACT ACGTGCCAGC AGCCGCGGTA ATACGTA-GG TGGCAAGCGT TATCCGGAA- TTATTGGGCG TAAAGCGCGC

PaKu4_-_Enterobacter_cloacae GCCTGATGCA GCCATGCCGC GTGTATGAAG AAGGCCTTCG GGTTGTAAAG TACTTTCAGC GGGGAGGAA- GGTGTTGTGG TTAATAACCA CAGCAATTGA CGTTACCCGC AGAAGAAGCA CCGGCTAACT CCGTGCCAGC AGCCGCGGTA ATACGGA-GG GTGCAAGCGT TAATCGGAA- TTACTGGGCG TAAAGCGCAC

Uncultured_Enterobacter_sp._(M GCCTGATGCA GCCATGCCGC GTGTATGAAG AAGGCCTTCG GGTTGTAAAG TACTTTCAGC GGGGAGGAA- GGTGTTGTGG TTAATAACCA CAGCAATTGA CGTTACCCGC AGAAGAAGCA CCGGCTAACT CCGTGCCAGC AGCCGCGGTA ATACGGA-GG GTGCAAGCGT TAATCGGAA- TTACTGGGCG TAAAGCGCAC

Enterobacter_cloacae_(KY930709 GCCTGATGCA GCCATGCCGC GTGTATGAAG AAGGCCTTCG GGTTGTAAAG TACTTTCAGC GGGGAGGAA- GGTGTTGTGG TTAATAACCA CAGCAATTGA CGTTACCCGC AGAAGAAGCA CCGGCTAACT CCGTGCCAGC AGCCGCGGTA ATACGGA-GG GTGCAAGCGT TAATCGGAA- TTACTGGGCG TAAAGCGCAC

PaKu5_-_Enterobacter_cloacae GCCTGATGCA GCCATGCCGC GTGTATGAAG AAGGCCTTCG GGTTGTAAAG TACTTTCAGC GGGGAGGAA- GGTGTTGTGG TTAATAACCA CAGCAATTGA CGTTACCCGC AGAAGAAGCA CCGGCTAACT CCGTGCCAGC AGCCGCGGTA ATACGGA-GG GTGCAAGCGT TAATCGGAA- TTACTGGGCG TAAAGCGCAC

Enterobacter_sp._(KU867838.1) GCCTGATGCA GCCATGCCGC GTGTATGAAG AAGGCCTTCG GGTTGTAAAG TACTTTCAGC GGGGAGGAA- GGTGTTGTGG TTAATAACCG CAGCAATTGA CGTTACCCGC AGAAGAAGCA CCGGCTAACT CCGTGCCAGC AGCCGCGGTA ATACGGA-GG GTGCAAGCGT TAATCGGAA- TTACTGGGCG TAAAGCGCAC

Enterobacter_cloacae_(KX242265 GCCTGATGCA GCCATGCCGC GTGTATGAAG AAGGCCTTCG GGTTGTAAAG TACTTTCAGC GGGGAGGAA- GGTGTTGTGG TTAATAACCA CAGCAATTGA CGTTACCCGC AGAAGAAGCA CCGGCTAACT CCGTGCCAGC AGCCGCGGTA ATACGGA-GG GTGCAAGCGT TAATCGGAA- TTACTGGGCG TAAAGCGCAC

Enterobacter_cloacae_(KP794925 GCCTGATGCA GCCATGCCGC GTGTATGAAG AAGGCCTTCG GGTTGTAAAG TACTTTCAGC GGGGAGGAA- GGTGTTGTGG TTAATAACCA CAGCAATTGA CGTTACCCGC AGAAGAAGCA CCGGCTAACT CCGTGCCAGC AGCCGCGGTA ATACGGA-GG GTGCAAGCGT TAATCGGAA- TTACTGGGCG TAAAGCGCAC

PaKu6_-_Staphylococcus_sciuri GCCTGACGGA GCAACGCCGC GTGAGTGATG AAGGTCTTCG GATCGTAAAA CTCTGTTGTT AGGGAAGAAC AAATTTGTTA GTAACT-GAA CAAGTCTTGA CGGTACCTAA CCAGAAAGCC ACGGCTAACT ACGTGCCAGC AGCCGCGGTA ATACGTA-GG TGGCAAGCGT TATCCGGAA- TTATTGGGCG TAAAGCGCGC

Staphylococcus_sciuri_(MG70600 GCCTGACGGA GCAACGCCGC GTGAGTGATG AAGGTCTTCG GATCGTAAAA CTCTGTTGTT AGGGAAGAAC AAATTTGTTA GTAACT-GAA CAAGTCTTGA CGGTACCTAA CCAGAAAGCC ACGGCTAACT ACGTGCCAGC AGCCGCGGTA ATACGTA-GG TGGCAAGCGT TATCCGGAA- TTATTGGGCG TAAAGCGCGC

Staphylococcus_lentus_(MG98829 GCCTGACGGA GCAACGCCGC GTGAGTGATG AAGGTCTTCG GATCGTAAAA CTCTGTTGTT AGGGAAGAAC AAATTTGTTA GTAACT-GAA CAAGTCTTGA CGGTACCTAA CCAGAAAGCC ACGGCTAACT ACGTGCCAGC AGCCGCGGTA ATACGTA-GG TGGCAAGCGT TATCCGGAA- TTATTGGGCG TAAAGCGCGC

Staphylococcus_sciuri_(MG51742 GCCTGACGGA GCAACGCCGC GTGAGTGATG AAGGTCTTCG GATCGTAAAA CTCTGTTGTT AGGGAAGAAC AAATTTGTTA GTAACT-GAA CAAGTCTTGA CGGTACCTAA CCAGAAAGCC ACGGCTAACT ACGTGCCAGC AGCCGCGGTA ATACGTA-GG TGGCAAGCGT TATCCGGAA- TTATTGGGCG TAAAGCGCGC

PaKu8_-_Pseudomonas_aeruginosa GCCTGATCCA GCCATGCCGC GTGTGTGAAG AAGGTCTTCG GATTGTAAAG CACTTTAAGT TGGGAGGAA- GGGCAGTAAG TTAATACCTT GCTGTTTTGA CGTTACCAAC AGAATAAGCA CCGGCTAACT TCGTGCCAGC AGCCGCGGTA ATACGAA-GG GTGCAAGCGT TAATCGGAA- TTACTGGGCG TAAAGCGCGC

Uncultured_bacterium_(FM996243 GCCTGATCCA GCCATGCCGC GTGTGTGAAG AAGGTCTTCG GATTGTAAAG CACTTTAAGT TGGGAGGAA- GGGCAGTAAG TTAATACCTT GCTGTTTTGA CGTTACCAAC AGAATAAGCA CCGGCTAACT TCGTGCCAGC AGCCGCGGTA ATACGAA-GG GTGCAAGCGT TAATCGGAA- TTACTGGGCG TAAAGCGCGC

Pseudomonas_aeruginosa_(MK4304 GCCTGATCCA GCCATGCCGC GTGTGTGAAG AAGGTCTTCG GATTGTAAAG CACTTTAAGT TGGGAGGAA- GGGCAGTAAG TTAATACCTT GCTGTTTTGA CGTTACCAAC AGAATAAGCA CCGGCTAACT TCGTGCCAGC AGCCGCGGTA ATACGAA-GG GTGCAAGCGT TAATCGGAA- TTACTGGGCG TAAAGCGCGC

PaKu9_-_Staphylococcus_arletta GCCTGACGGA GCAACGCCGC GTGAGTGATG AAGGGTTTCG GCTCGTAAAA CTCTGTTATT AGGGAAGAAC AAACGTGTAA GTAACT-GTG CACGTCTTGA CGGTACCTAA TCAGAAAGCC ACGGCTAACT ACGTGCCAGC AGCCGCGGTA ATACGTA-GG TGGCAAGCGT TATCCGGAA- TTATTGGGCG TAAAGCGCGC

Staphylococcus_arlettae_(KX344 GCCTGACGGA GCAACGCCGC GTGAGTGATG AAGGGTTTCG GCTCGTAAAA CTCTGTTATT AGGGAAGAAC AAACGTGTAA GTAACT-GTG CACGTCTTGA CGGTACCTAA TCAGAAAGCC ACGGCTAACT ACGTGCCAGC AGCCGCGGTA ATACGTA-GG TGGCAAGCGT TATCCGGAA- TTATTGGGCG TAAAGCGCGC

Staphylococcus_sp._(KU891836.1 GCCTGACGGA GCAACGCCGC GTGAGTGATG AAGGGTTTCG GCTCGTAAAA CTCTGTTATT AGGGAAGAAC AAACGTGTAA GTAACT-GTG CACGTCTTGA CGGTACCTAA TCAGAAAGCC ACGGCTAACT ACGTGCCAGC AGCCGCGGTA ATACGTA-GG TGGCAAGCGT TATCCGGAA- TTATTGGGCG TAAAGCGCGC

PaKu10_-_Aeromonas_caviae CCCTGATGCA GCCATGCCGC GTGTGTGAAG AAGGCCTTCG GGTTGTAAAG CACTTTCAGC GAGGAGGAA- AGGTCAGTAG CTAATATCTG CTGACTGTGA CGTTACTCGC AGAAGAAGCA CCGGCTAACT CCGTGCCAGC AGCCGCGGTA ATACGGA-GG GTGCAAGCGT TAATCGGAA- TTACTGGGCG TAAAGCGCAC

Uncultured_bacterium_(KX242397 CCCTGATGCA GCCATGCCGC GTGTGTGAAG AAGGCCTTCG GGTTGTAAAG CACTTTCAGC GAGGAGGAA- AGGTCAGTAG CTAATATCTG CTGACTGTGA CGTTACTCGC AGAAGAAGCA CCGGCTAACT CCGTGCCAGC AGCCGCGGTA ATACGGA-GG GTGCAAGCGT TAATCGGAA- TTACTGGGCG TAAAGCGCAC

Aeromonas_caviae_(HQ407268.1) CCCTGATGCA GCCATGCCGC GTGTGTGAAG AAGGCCTTCG GGTTGTAAAG CACTTTCAGC GAGGAGGAA- AGGTCAGTAG CTAATATCTG CTGACTGTGA CGTTACTCGC AGAAGAAGCA CCGGCTAACT CCGTGCCAGC AGCCGCGGTA ATACGGA-GG GTGCAAGCGT TAATCGGAA- TTACTGGGCG TAAAGCGCAC

Aeromonas_aquariorum_(EU085557 CCCTGATGCA GCCATGCCGC GTGTGTGAAG AAGGCCTTCG GGTTGTAAAG CACTTTCAGC GAGGAGGAA- AGGTCAGTAG CTAATATCTG CTGACTGTGA CGTTACTCGC AGAAGAAGCA CCGGCTAACT CCGTGCCAGC AGCCGCGGTA ATACGGA-GG GTGCAAGCGT TAATCGGAA- TTACTGGGCG TAAAGCGCAC

PaKu11_-_Aeromonas_caviae CCCTGATGCA GCCATGCCGC GTGTGTGAAG AAGGCCTTCG GGTTGTAAAG CACTTTCAGC GAGGAGGAA- AGGTCAGTAG CTAATATCTG CTGGCTGTGA CGTTACTCGC AGAAGAAGCA CCGGCTAACT CCGTGCCAGC AGCCGCGGTA ATACGGA-GG GTGCAAGCGT TAATCGGAA- TTACTGGGCG TAAAGCGCAC

Aeromonas_caviae_(KY087979.1) CCCTGATGCA GCCATGCCGC GTGTGTGAAG AAGGCCTTCG GGTTGTAAAG CACTTTCAGC GAGGAGGAA- AGGTCAGTAG CTAATATCTG CTGGCTGTGA CGTTACTCGC AGAAGAAGCA CCGGCTAACT CCGTGCCAGC AGCCGCGGTA ATACGGA-GG GTGCAAGCGT TAATCGGAA- TTACTGGGCG TAAAGCGCAC

Uncultured_Aeromonas_sp._(KU94 CCCTGATGCA GCCATGCCGC GTGTGTGAAG AAGGCCTTCG GGTTGTAAAG CACTTTCAGC GAGGAGGAA- AGGTCAGTAG CTAATATCTG CTGGCTGTGA CGTTACTCGC AGAAGAAGCA CCGGCTAACT CCGTGCCAGC AGCCGCGGTA ATACGGA-GG GTGCAAGCGT TAATCGGAA- TTACTGGGCG TAAAGCGCAC

PaKu12_-_Staphylococcus_warner GCCTGACGGA GCAACGCCGC GTGAGTGATG AAGGTCTTCG GATCGTAAAA CTCTGTTATC AGGGAAGAAC AAATGTGTAA GTAACT-GTG CACATCTTGA CGGTACCTGA TCAGAAAGCC ACGGCTAACT ACGTGCCAGC AGCCGCGGTA ATACGTA-GG TGGCAAGCGT TATCCGGAA- TTATTGGGCG TAAAGCGCGC

Staphylococcus_warneri_(MH0149 GCCTGACGGA GCAACGCCGC GTGAGTGATG AAGGTCTTCG GATCGTAAAA CTCTGTTATC AGGGAAGAAC AAATGTGTAA GTAACT-GTG CACATCTTGA CGGTACCTGA TCAGAAAGCC ACGGCTAACT ACGTGCCAGC AGCCGCGGTA ATACGTA-GG TGGCAAGCGT TATCCGGAA- TTATTGGGCG TAAAGCGCGC

Staphylococcus_warneri_(KF3063 GCCTGACGGA GCAACGCCGC GTGAGTGATG AAGGTCTTCG GATCGTAAAA CTCTGTTATC AGGGAAGAAC AAATGTGTAA GTAACT-GTG CACATCTTGA CGGTACCTGA TCAGAAAGCC ACGGCTAACT ACGTGCCAGC AGCCGCGGTA ATACGTA-GG TGGCAAGCGT TATCCGGAA- TTATTGGGCG TAAAGCGCGC

PaKu13_-_Bacillus_megaterium GTCTGACGGA GCAACGCCGC GTGAGTGATG AAGGCTTTCG GGTCGTAAAA CTCTGTTGTT AGGGAAGAAC AAGTACGAGA GTAACT-GCT CGTACCTTGA CGGTACCTAA CCAGAAAGCC ACGGCTAACT ACGTGCCAGC AGCCGCGGTA ATACGTA-GG TGGCAAGCGT TATCCGGAA- TTATTGGGCG TAAAGCGCGC

Bacillus_sp._(MK691444.1) GTCTGACGGA GCAACGCCGC GTGAGTGATG AAGGCTTTCG GGTCGTAAAA CTCTGTTGTT AGGGAAGAAC AAGTACGAGA GTAACT-GCT CGTACCTTGA CGGTACCTAA CCAGAAAGCC ACGGCTAACT ACGTGCCAGC AGCCGCGGTA ATACGTA-GG TGGCAAGCGT TATCCGGAA- TTATTGGGCG TAAAGCGCGC

Bacillus_sp._(LC373523.1) GTCTGACGGA GCAACGCCGC GTGAGTGATG AAGGCTTTCG GGTCGTAAAA CTCTGTTGTT AGGGAAGAAC AAGTACGAGA GTAACT-GCT CGTACCTTGA CGGTACCTAA CCAGAAAGCC ACGGCTAACT ACGTGCCAGC AGCCGCGGTA ATACGTA-GG TGGCAAGCGT TATCCGGAA- TTATTGGGCG TAAAGCGCGC

Bacillus_aryabhattai_(MK474942 GTCTGACGGA GCAACGCCGC GTGAGTGATG AAGGCTTTCG GGTCGTAAAA CTCTGTTGTT AGGGAAGAAC AAGTACGAGA GTAACT-GCT CGTACCTTGA CGGTACCTAA CCAGAAAGCC ACGGCTAACT ACGTGCCAGC AGCCGCGGTA ATACGTA-GG TGGCAAGCGT TATCCGGAA- TTATTGGGCG TAAAGCGCGC

Bacillus_aryabhattai_(MK474941 GTCTGACGGA GCAACGCCGC GTGAGTGATG AAGGCTTTCG GGTCGTAAAA CTCTGTTGTT AGGGAAGAAC AAGTACGAGA GTAACT-GCT CGTACCTTGA CGGTACCTAA CCAGAAAGCC ACGGCTAACT ACGTGCCAGC AGCCGCGGTA ATACGTA-GG TGGCAAGCGT TATCCGGAA- TTATTGGGCG TAAAGCGCGC

Bacillus_megaterium_(KX298860. GTCTGACGGA GCAACGCCGC GTGAGTGATG AAGGCTTTCG GGTCGTAAAA CTCTGTTGTT AGGGAAGAAC AAGTACGAGA GTAACT-GCT CGTACCTTGA CGGTACCTAA CCAGAAAGCC ACGGCTAACT ACGTGCCAGC AGCCGCGGTA ATACGTA-GG TGGCAAGCGT TATCCGGAA- TTATTGGGCG TAAAGCGCGC

PaKu14_-_Pseudomonas_stutzeri GCCTGATCCA GCCATGCCGC GTGTGTGAAG AAGGTCTTCG GATTGTAAAG CACTTTAAGT TGGGAGGAA- GGGCAGTAAG TTAATACCTT GCTGTTTTGA CGTTACCAAC AGAATAAGCA CCGGCTAACT TCGTGCCAGC AGCCGCGGTA ATACGAA-GG GTGCAAGCGT TAATCGGAA- TTACTGGGCG TAAAGCGCGC

Pseudomonas_stutzeri_(MG892782 GCCTGATCCA GCCATGCCGC GTGTGTGAAG AAGGTCTTCG GATTGTAAAG CACTTTAAGT TGGGAGGAA- GGGCAGTAAG TTAATACCTT GCTGTTTTGA CGTTACCAAC AGAATAAGCA CCGGCTAACT TCGTGCCAGC AGCCGCGGTA ATACGAA-GG GTGCAAGCGT TAATCGGAA- TTACTGGGCG TAAAGCGCGC

Pseudomonas_stutzeri_(MG595371 GCCTGATCCA GCCATGCCGC GTGTGTGAAG AAGGTCTTCG GATTGTAAAG CACTTTAAGT TGGGAGGAA- GGGCAGTAAG TTAATACCTT GCTGTTTTGA CGTTACCAAC AGAATAAGCA CCGGCTAACT TCGTGCCAGC AGCCGCGGTA ATACGAA-GG GTGCAAGCGT TAATCGGAA- TTACTGGGCG TAAAGCGCGC

PaKu15_-_Rhizobium_sp. GCCTGATCCA GCCATGCCGC GTGAGTGATG AAGGCCTTAG GGTTGTAAAG CTCTTTCACC GATGAAGATA A--------- ---------- -------TGA CGGTAGTCGG AGAAGAAGCC CCGGCTAACT TCGTGCCAGC AGCCGCGGTA ATACGAA-GG GGGCTAGCGT TGTTCGGAA- TTACTGGGCG TAAAGCGCAC

Rhizobium_pusense_(CP039895.1) GCCTGATCCA GCCATGCCGC GTGAGTGATG AAGGCCTTAG GGTTGTAAAG CTCTTTCACC GATGAAGATA A--------- ---------- -------TGA CGGTAGTCGG AGAAGAAGCC CCGGCTAACT TCGTGCCAGC AGCCGCGGTA ATACGAA-GG GGGCTAGCGT TGTTCGGAA- TTACTGGGCG TAAAGCGCAC

Rhizobium_sp._(KY971009.1) GCCTGATCCA GCCATGCCGC GTGAGTGATG AAGGCCTTAG GGTTGTAAAG CTCTTTCACC GATGAAGATA A--------- ---------- -------TGA CGGTAGTCGG AGAAGAAGCC CCGGCTAACT TCGTGCCAGC AGCCGCGGTA ATACGAA-GG GGGCTAGCGT TGTTCGGAA- TTACTGGGCG TAAAGCGCAC

PaKu7_-_Stenotrophomonas_malto GCCTGATCCA GCCATACCGC GTGGGTGAAG AAGGCCTTCG GGTTGTAAAG CCCTTTTGTT GGGAAAGAA- ATCCAGCTGG CTAATACCCG GTTGGGATGA CGGTACCCAA AGAATAAGCA CCGGCTAACT TCGTGCCAGC AGCCGCGGTA ATACGAA-GG GTGCAAGCGT TACTCGGAA- TTACTGGGCG TAAAGCGTGC

Stenotrophomonas_maltophilia_( GCCTGATCCA GCCATACCGC GTGGGTGAAG AAGGCCTTCG GGTTGTAAAG CCCTTTTGTT GGGAAAGAA- ATCCAGCTGG CTAATACCCG GTTGGGATGA CGGTACCCAA AGAATAAGCA CCGGCTAACT TCGTGCCAGC AGCCGCGGTA ATACGAA-GG GTGCAAGCGT TACTCGGAA- TTACTGGGCG TAAAGCGTGC

Stenotrophomonas_maltophilia_( GCCTGATCCA GCCATACCGC GTGGGTGAAG AAGGCCTTCG GGTTGTAAAG CCCTTTTGTT GGGAAAGAA- ATCCAGCTGG CTAATACCCG GTTGGGATGA CGGTACCCAA AGAATAAGCA CCGGCTAACT TCGTGCCAGC AGCCGCGGTA ATACGAA-GG GTGCAAGCGT TACTCGGAA- TTACTGGGCG TAAAGCGTGC

PaKu16_-_Stenotrophomonas_malt GCCTGATCCA GCCATACCGC GTGGGTGAAG AAGGCCTTCG GGTTGTAAAG CCCTTTTGTT GGGAAAGAA- ATCCAGCTGG CTAATACCCG GTTGGGATGA CGGTACCCAA AGAATAAGCA CCGGCTAACT TCGTGCCAGC AGCCGCGGTA ATACGAA-GG GTGCAAGCGT TACTCGGAA- TTACTGGGCG TAAAGCGTGC

Stenotrophomonas_maltophilia_( GCCTGATCCA GCCATACCGC GTGGGTGAAG AAGGCCTTCG GGTTGTAAAG CCCTTTTGTT GGGAAAGAA- ATCCAGCTGG CTAATACCCG GTTGGGATGA CGGTACCCAA AGAATAAGCA CCGGCTAACT TCGTGCCAGC AGCCGCGGTA ATACGAA-GG GTGCAAGCGT TACTCGGAA- TTACTGGGCG TAAAGCGTGC

Stenotrophomonas_maltophilia_( GCCTGATCCA GCCATACCGC GTGGGTGAAG AAGGCCTTCG GGTTGTAAAG CCCTTTTGTT GGGAAAGAA- ATCCAGCTGG CTAATACCCG GTTGGGATGA CGGTACCCAA AGAATAAGCA CCGGCTAACT TCGTGCCAGC AGCCGCGGTA ATACGAA-GG GTGCAAGCGT TACTCGGAA- TTACTGGGCG TAAAGCGTGC

PaKu17_-_Stenotrophomonas_malt GCCTGATCCA GCCATACCGC GTGGGTGAAG AAGGCCTTCG GGTTGTAAAG CCCTTTTGTT GGGAAAGAA- ATCCAGCTGG CTAATACCCG GTTGGGATGA CGGTACCCAA AGAATAAGCA CCGGCTAACT TCGTGCCAGC AGCCGCGGTA ATACGAA-GG GTGCAAGCGT TACTCGGAA- TTACTGGGCG TAAAGCGTGC

PaKu26_-_Stenotrophomonas_malt GCCTGATCCA GCCATACCGC GTGGGTGAAG AAGGCCTTCG GGTTGTAAAG CCCTTTTGTT GGGAAAGAA- ATCCAGCTGG CTAATACCCG GTTGGGATGA CGGTACCCAA AGAATAAGCA CCGGCTAACT TCGTGCCAGC AGCCGCGGTA ATACGAA-GG GTGCAAGCGT TACTCGGAA- TTACTGGGCG TAAAGCGTGC

Stenotrophomonas_maltophilia_( GCCTGATCCA GCCATACCGC GTGGGTGAAG AAGGCCTTCG GGTTGTAAAG CCCTTTTGTT GGGAAAGAA- ATCCAGCTGG CTAATACCCG GTTGGGATGA CGGTACCCAA AGAATAAGCA CCGGCTAACT TCGTGCCAGC AGCCGCGGTA ATACGAA-GG GTGCAAGCGT TACTCGGAA- TTACTGGGCG TAAAGCGTGC

PaKu18_-_Stenotrophomonas_pana GCCTGATCCA GCCATGCCGC GTGGGTGAAG AAGGCCTTCG GGTTGTAAAG CCCTTTTGTT GGGAAAGAA- AAGCAGTCGA TTAATACTCG ATTGTTCTGA CGGTACCCAA AGAATAAGCA CCGGCTAACT TCGTGCCAGC AGCCGCGGTA ATACGAA-GG GTGCAAGCGT TACTCGGAA- TTACTGGGCG TAAAGCGTGC

Stenotrophomonas_panacihumi_(M GCCTGATCCA GCCATGCCGC GTGGGTGAAG AAGGCCTTCG GGTTGTAAAG CCCTTTTGTT GGGAAAGAA- AAGCAGTCGA TTAATACTCG ATTGTTCTGA CGGTACCCAA AGAATAAGCA CCGGCTAACT TCGTGCCAGC AGCCGCGGTA ATACGAA-GG GTGCAAGCGT TACTCGGAA- TTACTGGGCG TAAAGCGTGC

Stenotrophomonas_panacihumi_(K GCCTGATCCA GCCATGCCGC GTGGGTGAAG AAGGCCTTCG GGTTGTAAAG CCCTTTTGTT GGGAAAGAA- AAGCAGTCGA TTAATACTCG ATTGTTCTGA CGGTACCCAA AGAATAAGCA CCGGCTAACT TCGTGCCAGC AGCCGCGGTA ATACGAA-GG GTGCAAGCGT TACTCGGAA- TTACTGGGCG TAAAGCGTGC

PaKu19_-_Bacillus_licheniformi GTCTGACGGA GCAACGCCGC GTGAGTGATG AAGGTTTTCG GATCGTAAAA CTCTGTTGTT AGGGAAGAAC AAGTACCGTT CGAATAGGGC GGTACCTTGA CGGTACCTAA CCAGAAAGCC ACGGCTAACT ACGTGCCAGC AGCCGCGGTA ATACGTA-GG TGGCAAGCGT TGTCCGGAA- TTATTGGGCG TAAAGCGCGC

Bacillus_licheniformis_(KJ5722 GTCTGACGGA GCAACGCCGC GTGAGTGATG AAGGTTTTCG GATCGTAAAA CTCTGTTGTT AGGGAAGAAC AAGTACCGTT CGAATAGGGC GGCACCTTGA CGGTACCTAA CCAGAAAGCC ACGGCTAACT ACGTGCCAGC AGCCGCGGTA ATACGTA-GG TGGCAAGCGT TGTCCGGAA- TTATTGGGCG TAAAGCGCGC

Bacillus_licheniformis_(KT1536 GTCTGACGGA GCAACGCCGC GTGAGTGATG AAGGTTTTCG GATCGTAAAA CTCTGTTGTT AGGGAAGAAC AAGTACCGTT CGAATAGGGC GGTACCTTGA CGGTACCTAA CCAGAAAGCC ACGGCTAACT ACGTGCCAGC AGCCGCGGTA ATACGTA-GG TGGCAAGCGT TGTCCGGAA- TTATTGGGCG TAAAGCGCGC

PaKu20_-__Pantoea_dispersa GCCTGATGCA GCCATGCCGC GTGTATGAAG AAGGCCTTCG GGTTGTAAAG TACTTTCAGC GGGGAGGAA- GGCGGTGAGG TTAATAACCT CGCCGATTGA CGTTACCCGC AGAAGAAGCA CCGGCTAACT CCGTGCCAGC AGCCGCGGTA ATACGGA-GG GTGCAAGCGT TAATCGGAA- TTACTGGGCG TAAAGCGCAC

Pantoea_dispersa_(KY292463.1) GCCTGATGCA GCCATGCCGC GTGTATGAAG AAGGCCTTCG GGTTGTAAAG TACTTTCAGC GGGGAGGAA- GGCGGTGAGG TTAATAACCT YGCCGATTGA CGTTACCCGC AGAAGAAGCA CCGGCTAACT CCGTGCCAGC AGCCGCGGTA ATACGGA-GG GTGCAAGCGT TAATCGGAA- TTACTGGGCG TAAAGCGCAC

Pantoea_dispersa_(KY882077.1) GCCTGATGCA GCCATGCCGC GTGTATGAAG AAGGCCTTCG GGTTGTAAAG TACTTTCAGC GGGGAGGAA- GGCGGTGAGG TTAATAACCT CGCCGATTGA CGTTACCCGC AGAAGAAGCA CCGGCTAACT CCGTGCCAGC AGCCGCGGTA ATACGGA-GG GTGCAAGCGT TAATCGGAA- TTACTGGGCG TAAAGCGCAC

PaKu21_-_Bacillus_sonorensis GTCTGACGGA GCAACGCCGC GTGAGTGATG AAGGTTTTCG GATCGTAAAA CTCTGTTGTT AGGGAAGAAC AAGTACCGTT CGAACAGGGC GGTACCTTGA CGGTACCTAA CCAGAAAGCC ACGGCTAACT ACGTGCCAGC AGCCGCGGTA ATACGTA-GG TGGCAAGCGT TGTCCGGAA- TTATTGGGCG TAAAGCGCGC

Bacillus_sonorensis_(KU551167. GTCTGACGGA GCAACGCCGC GTGAGTGATG AAGGTTTTCG GATCGTAAAA CTCTGTTGTT AGGGAAGAAC AAGTACCGTT CGAACAGGGC GGTACCTTGA CGGTACCTAA CCAGAAAGCC ACGGCTAACT ACGTGCCAGC AGCCGCGGTA ATACGTA-GG TGGCAAGCGT TGTCCGGAA- TTATTGGGCG TAAAGCGCGC

Bacillus_sonorensis_(FN397516. GTCTGACGGA GCAACGCCGC GTGAGTGATG AAGGTTTTCG GATCGTAAAA CTCTGTTGTT AGGGAAGAAC AAGTACCGTT CGAACAGGGC GGTRCCTTGA CGGTACCTAA CCAGAAAGCC ACGGCTAACT ACGTGCCAGC AGCCGCGGTA ATACGTA-GG TGGCAAGCGT TGTCCGGAA- TTATTGGGCG TAAAGCGCGC

Bacillus_sonorensis_(KU551137. GTCTGACGGA GCAACGCCGC GTGAGTGATG AAGGTTTTCG GATCGTAAAA CTCTGTTGTT AGGGAAGAAC AAGTACCGTT CGAACAGGGC GGTACCTTGA CGGTACCTAA CCAGAAAGCC ACGGCTAACT ACGTGCCAGC AGCCGCGGTA ATACGTA-GG TGGCAAGCGT TGTCCGGAA- TTATTGGGCG TAAAGCGCGC

PaKu24_-_Bacillus_sonorensis GTCTGACGGA GCAACGCCGC GTGAGTGATG AAGGTTTTCG GATCGTAAAA CTCTGTTGTT AGGGAAGAAC AAGTACCGTT CGAACAGGGC GGTACCTTGA CGGTACCTAA CCAGAAAGCC ACGGCTAACT ACGTGCCAGC AGCCGCGGTA ATACGTA-GG TGGCAAGCGT TGTCCGGAA- TTATTGGGCG TAAAGCGCGC

PaKu22_-_Bacillus_subtilis GTCTGACGGA GCAACGCCGC GTGAGTGATG AAGGTTTTCG GATCGTAAAG CTCTGTTGTT AGGGAAGAAC AAGTACCGTT CGAATAGGGC GGTACCTTGA CGGTACCTAA CCAGAAAGCC ACGGCTAACT ACGTGCCAGC AGCCGCGGTA ATACGTA-GG TGGCAAGCGT TGTCCGGAA- TTATTGGGCG TAAAGGGCTC

Bacillus_tequilensis_(MK880583 GTCTGACGGA GCAACGCCGC GTGAGTGATG AAGGTTTTCG GATCGTAAAG CTCTGTTGTT AGGGAAGAAC AAGTACCGTT CGAATAGGGC GGTACCTTGA CGGTACCTAA CCAGAAAGCC ACGGCTAACT ACGTGCCAGC AGCCGCGGTA ATACGTA-GG TGGCAAGCGT TGTCCGGAA- TTATTGGGCG TAAAGGGCTC

Bacillus_subtilis_(MK765023.1) GTCTGACGGA GCAACGCCGC GTGAGTGATG AAGGTTTTCG GATCGTAAAG CTCTGTTGTT AGGGAAGAAC AAGTACCGTT CGAATAGGGC GGTACCTTGA CGGTACCTAA CCAGAAAGCC ACGGCTAACT ACGTGCCAGC AGCCGCGGTA ATACGTA-GG TGGCAAGCGT TGTCCGGAA- TTATTGGGCG TAAAGGGCTC

PaKu23_-_Bacillus_subtilis GTCTGACGGA GCAACGCCGC GTGAGTGATG AAGGTTTTCG GATCGTAAAG CTCTGTTGTT AGGGAAGAAC AAGTACCGTT CGAATAGGGC GGTACCTTGA CGGTACCTAA CCAGAAAGCC ACGGCTAACT ACGTGCCAGC AGCCGCGGTA ATACGTA-GG TGGCAAGCGT TGTCCGGAA- TTATTGGGCG TAAAGGGCTC

Bacillus_subtilis_(MK511833.1) GTCTGACGGA GCAACGCCGC GTGAGTGATG AAGGTTTTCG GATCGTAAAG CTCTGTTGTT AGGGAAGAAC AAGTACCGTT CGAATAGGGC GGTACCTTGA CGGTACCTAA CCAGAAAGCC ACGGCTAACT ACGTGCCAGC AGCCGCGGTA ATACGTA-GG TGGCAAGCGT TGTCCGGAA- TTATTGGGCG TAAAGGGCTC

Bacillus_subtilis_(KX450400.1) GTCTGACGGA GCAACGCCGC GTGAGTGATG AAGGTTTTCG GATCGTAAAG CTCTGTTGTT AGGGAAGAAC AAGTACCGTT CGAATAGGGC GGTACCTTGA CGGTACCTAA CCAGAAAGCC ACGGCTAACT ACGTGCCAGC AGCCGCGGTA ATACGTA-GG TGGCAAGCGT TGTCCGGAA- TTATTGGGCG TAAAGGGCTC

PaKu25_-_Bacillus_cereus GTCTGACGGA GCAACGCCGC GTGAGTGATG AAGGCTTTCG GGTCGTAAAA CTCTGTTGTT AGGGAAGAAC AAGTGCTAGT TGAATAAGCT GGCACCTTGA CGGTACCTAA CCAGAAAGCC ACGGCTAACT ACGTGCCAGC AGCCGCGGTA ATACGTA-GG TGGCAAGCGT TATCCGGAA- TTATTGGGCG TAAAGCGCGC

Bacillus_thuringiensis_(CP0378 GTCTGACGGA GCAACGCCGC GTGAGTGATG AAGGCTTTCG GGTCGTAAAA CTCTGTTGTT AGGGAAGAAC AAGTGCTAGT TGAATAAGCT GGCACCTTGA CGGTACCTAA CCAGAAAGCC ACGGCTAACT ACGTGCCAGC AGCCGCGGTA ATACGTA-GG TGGCAAGCGT TATCCGGAA- TTATTGGGCG TAAAGCGCGC

Bacillus_mobilis_(CP031443.1) GTCTGACGGA GCAACGCCGC GTGAGTGATG AAGGCTTTCG GGTCGTAAAA CTCTGTTGTT AGGGAAGAAC AAGTGCTAGT TGAATAAGCT GGCACCTTGA CGGTACCTAA CCAGAAAGCC ACGGCTAACT ACGTGCCAGC AGCCGCGGTA ATACGTA-GG TGGCAAGCGT TATCCGGAA- TTATTGGGCG TAAAGCGCGC

Bacillus_thuringiensis_(CP0357 GTCTGACGGA GCAACGCCGC GTGAGTGATG AAGGCTTTCG GGTCGTAAAA CTCTGTTGTT AGGGAAGAAC AAGTGCTAGT TGAATAAGCT GGCACCTTGA CGGTACCTAA CCAGAAAGCC ACGGCTAACT ACGTGCCAGC AGCCGCGGTA ATACGTA-GG TGGCAAGCGT TATCCGGAA- TTATTGGGCG TAAAGCGCGC

Bacillus_cereus_(CP030982.1) GTCTGACGGA GCAACGCCGC GTGAGTGATG AAGGCTTTCG GGTCGTAAAA CTCTGTTGTT AGGGAAGAAC AAGTGCTAGT TGAATAAGCT GGCACCTTGA CGGTACCTAA CCAGAAAGCC ACGGCTAACT ACGTGCCAGC AGCCGCGGTA ATACGTA-GG TGGCAAGCGT TATCCGGAA- TTATTGGGCG TAAAGCGCGC

Bacillus_cereus_(MH068823.1) GTCTGACGGA GCAACGCCGC GTGAGTGATG AAGGCTTTCG GGTCGTAAAA CTCTGTTGTT AGGGAAGAAC AAGTGCTAGT TGAATAAGCT GGCACCTTGA CGGTACCTAA CCAGAAAGCC ACGGCTAACT ACGTGCCAGC AGCCGCGGTA ATACGTA-GG TGGCAAGCGT TATCCGGAA- TTATTGGGCG TAAAGCGCGC

Bacillus_thuringiensis_(MG7453 GTCTGACGGA GCAACGCCGC GTGAGTGATG AAGGCTTTCG GGTCGTAAAA CTCTGTTGTT AGGGAAGAAC AAGTGCTAGT TGAATAAGCT GGCACCTTGA CGGTACCTAA CCAGAAAGCC ACGGCTAACT ACGTGCCAGC AGCCGCGGTA ATACGTA-GG TGGCAAGCGT TATCCGGAA- TTATTGGGCG TAAAGCGCGC

Rhizobium_massiliae(Country-_T ---------- ---------- ---------- ---------- ---------- ---------- ---------- ---------- ---------- ---------- ---------- ---------- ---------- ---------- ---------- --------GG GCGCTAGCGT TGTTCGGAA- TTACTGGGCG TAAAGCGCAC

Stenotrophomonas_maltophilia_( ---------- ---------- ---------- ---------- ---------- ---------- ---------- ---------- ---------- ---------- ------CCAA AGAATAAGCA CCGGCTAACT TCGTGCCAGC AGCCGCGGTA ATACGAAAGG GTGCAAGCGT TACTCGGAAA TTACTGGGCG TAAAGCGTGC

Serratia_sp._(Country-_Tunisia GCCTGATGCA GCCATGCCGC GTGTGTGAAG AAGACCCTCG GGTTGTAAAG CACTTTTAGC GAGGAGGAA- GGTGGCGAGC TTAATACGCT CATCAATTGA CGTTACCCGC AGAAGAAGCA TCGGCTAACT CTGTGTCATC AGCCGCGGTA ATACGGA-GG GTGCAAGCGT TAATCGGAA- TTACTGGGCG TAAAGCGCAC

Staphylococcus_saprophyticus_( ---------- ---------- ---------- ---------- ---------- ---------- --------AC AAATGTGTAA GTAACT-GTG CACGTCTTGA CGGTACCTAA TCAGAAAGCC ACGGCTAACT ACGTGCCAGC AGCCGCGGTA ATACGTA-GG TGGCAAGCGT TATCCGGAA- TTATTGGGCG TAAAGCGCGC

Staphylococcus_saprophyticus_( ---------- ---------- ---------- ---------- ---------- ---------- ---------- ---------- ---------- ---------- ---------- ---------- ---------- ---------- ---CGCGGTA ATACGTA-GG TGGCAAGCGT TATCCGGAA- TTATTGGGCG TAAAGCGCGC

Bacillus_subtilis_(Country-Ira ---------- ---------- ---------- ---------- ---------- ---------- --GGAAGAAC AAGTCCCGTT CGAATAGGGC GGTACCTTGA CGGTACCTAA CCAGAAAGCC ACGGCTAACT ACGTGCCAGC AGCCGCGGTA ATACGTA-GG TGGCAAGCGT TGTCCGGAA- TTATTGGGCG TAAAGGGCTC

Bacillus_cereus_(Country-UK)_( GTCTGACGGA GCAACGCCGC GTGAGTGATG AAGGCTTTCG GGTCGTAAAA CTCTGTTGTT AGGGAAGAAC AAGTGCTAGT TGAATAAGCT GGCACCTTGA CGGTACCTAA CCAGAAAGCC ACGGCTAACT ACGTGCCAGC AGCCGCGGTA ATACGTA-GG TGGCAAGCGT TATCCGGAA- TTATTGGGCG TAAAGCGCGC

....|....| ....|....| ....|....| ....|....| ....|....| ....|....| ....|....| ....|....| ....|....| ....|....| ....|....| ....|....| ....|....| ....|....| ....|....| ....|....| ....|....| ....|....| ....|....| ....|....|

605 615 625 635 645 655 665 675 685 695 705 715 725 735 745 755 765 775 785 795

PaKu1_-_Serratia_marcescens GCAGGCGGTT TGTTAA-GTC AGATGTGAAA TCCCCGGGCT CAACCTGGGA ACTG-CATTT GAAACTGGCA AGCTAGAGTC TCGTAGAGGG GGGTAGAATT CCAGGTGTAG CGGTGAAATG CGTAGAGATC TGGAGGAATA CCGGTGGCGA AGGCGGCCCC CTGGACGAAG ACTGACGCTC AGGTGCGAAA GCGTGGGGAG

Serratia_marcescens_(KX911721. GCAGGCGGTT TGTTAA-GTC AGATGTGAAA TCCCCGGGCT CAACCTGGGA ACTG-CATTT GAAACTGGCA AGCTAGAGTC TCGTAGAGGG GGGTAGAATT CCAGGTGTAG CGGTGAAATG CGTAGAGATC TGGAGGAATA CCGGTGGCGA AGGCGGCCCC CTGGACGAAG ACTGACGCTC AGGTGCGAAA GCGTGGGGAG

Serratia_marcescens_(KT992361. GCAGGCGGTT TGTTAA-GTC AGATGTGAAA TCCCCGGGCT CAACCTGGGA ACTG-CATTT GAAACTGGCA AGCTAGAGTC TCGTAGAGGG GGGTAGAATT CCAGGTGTAG CGGTGAAATG CGTAGAGATC TGGAGGAATA CCGGTGGCGA AGGCGGCCCC CTGGACGAAG ACTGACGCTC AGGTGCGAAA GCGTGGGGAG

PaKu2_-_Enterobacter_sp. GCAGGCGGTC TGTCAA-GTC GGATGTGAAA TCCCCGGGCT CAACCTGGGA ACTG-CATTC GAAACTGGCA GGCTAGAGTC TTGTAGAGGG GGGTAGAATT CCAGGTGTAG CGGTGAAATG CGTAGAGATC TGGAGGAATA CCGGTGGCGA AGGCGGCCCC CTGGACAAAG ACTGACGCTC AGGTGCGAAA GCGTGGGGAG

Enterobacter_sp._(MG280962.1) GCAGGCGGTC TGTCAA-GTC GGATGTGAAA TCCCCGGGCT CAACCTGGGA ACTG-CATTC GAAACTGGCA GGCTAGAGTC TTGTAGAGGG GGGTAGAATT CCAGGTGTAG CGGTGAAATG CGTAGAGATC TGGAGGAATA CCGGTGGCGA AGGCGGCCCC CTGGACAAAG ACTGACGCTC AGGTGCGAAA GCGTGGGGAG

Enterobacter_hormaechei_(CP029 GCAGGCGGTC TGTCAA-GTC GGATGTGAAA TCCCCGGGCT CAACCTGGGA ACTG-CATTC GAAACTGGCA GGCTAGAGTC TTGTAGAGGG GGGTAGAATT CCAGGTGTAG CGGTGAAATG CGTAGAGATC TGGAGGAATA CCGGTGGCGA AGGCGGCCCC CTGGACAAAG ACTGACGCTC AGGTGCGAAA GCGTGGGGAG

PaKu3_-_Staphylococcus_saproph GTAGGCGGTT TCTTAA-GTC TGATGTGAAA GCCCACGGCT CAACCGTGGA GG-GTCATTG GAAACTGGGA AACTTGAGTG CAGAAGAGGA AAGTGGAATT CCATGTGTAG CGGTGAAATG CGCAGAGATA TGGAGGAACA CCAGTGGCGA AGGCGACTTT CTGGTCTGTA ACTGACGCTG ATGTGCGAAA GCGTGGGGAT

Staphylococcus_saprophyticus_( GTAGGCGGTT TCTTAA-GTC TGATGTGAAA GCCCACGGCT CAACCGTGGA GG-GTCATTG GAAACTGGGA AACTTGAGTG CAGAAGAGGA AAGTGGAATT CCATGTGTAG CGGTGAAATG CGCAGAGATA TGGAGGAACA CCAGTGGCGA AGGCGACTTT CTGGTCTGTA ACTGACGCTG ATGTGCGAAA GCGTGGGGAT

Staphylococcus_saprophyticus_( GTAGGCGGTT TCTTAA-GTC TGATGTGAAA GCCCACGGCT CAACCGTGGA GG-GTCATTG GAAACTGGGA AACTTGAGTG CAGAAGAGGA AAGTGGAATT CCATGTGTAG CGGTGAAATG CGCAGAGATA TGGAGGAACA CCAGTGGCGA AGGCGACTTT CTGGTCTGTA ACTGACGCTG ATGTGCGAAA GCGTGGGGAT

PaKu4_-_Enterobacter_cloacae GCAGGCGGTC TGTCAA-GTC GGATGTGAAA TCCCCGGGCT CAACCTGGGA ACTG-CATTC GAAACTGGCA GGCTAGAGTC TTGTAGAGGG GGGTAGAATT CCAGGTGTAG CGGTGAAATG CGTAGAGATC TGGAGGAATA CCGGTGGCGA AGGCGGCCCC CTGGACAAAG ACTGACGCTC AGGTGCGAAA GCGTGGGGAG

Uncultured_Enterobacter_sp._(M GCAGGCGGTC TGTCAA-GTC GGATGTGAAA TCCCCGGGCT CAACCTGGGA ACTG-CATTC GAAACTGGCA GGCTAGAGTC TTGTAGAGGG GGGTAGAATT CCAGGTGTAG CGGTGAAATG CGTAGAGATC TGGAGGAATA CCGGTGGCGA AGGCGGCCCC CTGGACAAAG ACTGACGCTC AGGTGCGAAA GCGTGGGGAG

Enterobacter_cloacae_(KY930709 GCAGGCGGTC TGTCAA-GTC GGATGTGAAA TCCCCGGGCT CAACCTGGGA ACTG-CATTC GAAACTGGCA GGCTAGAGTC TTGTAGAGGG GGGTAGAATT CCAGGTGTAG CGGTGAAATG CGTAGAGATC TGGAGGAATA CCGGTGGCGA AGGCGGCCCC CTGGACAAAG ACTGACGCTC AGGTGCGAAA GCGTGGGGAG

PaKu5_-_Enterobacter_cloacae GCAGGCGGTC TGTCAA-GTC GGATGTGAAA TCCCCGGGCT CAACCTGGGA ACTG-CATTC GAAACTGGCA GGCTGGAGTC TTGTAGAGGG GGGTAGAATT CCAGGTGTAG CGGTGAAATG CGTAGAGATC TGGAGGAATA CCGGTGGCGA AGGCGGCCCC CTGGACAAAG ACTGACGCTC AGGTGCGAAA GCGTGGGGAG

Enterobacter_sp._(KU867838.1) GCAGGCGGTC TGTCAA-GTC GGATGTGAAA TCCCCGGGCT CAACCTGGGA ACTG-CATTC GAAACTGGCA GGCTAGAGTC TTGTAGAGGG GGGTAGAATT CCAGGTGTAG CGGTGAAATG CGTAGAGATC TGGAGGAATA CCGGTGGCGA AGGCGGCCCC CTGGACAAAG ACTGACGCTC AGGTGCGAAA GCGTGGGGAG

Enterobacter_cloacae_(KX242265 GCAGGCGGTC TGTCAA-GTC GGATGTGAAA TCCCCGGGCT CAACCTGGGA ACTG-CATTC GAAACTGGCA GGCTGGAGTC TTGTAGAGGG GGGTAGAATT CCAGGTGTAG CGGTGAAATG CGTAGAGATC TGGAGGAATA CCGGTGGCGA AGGCGGCCCC CTGGACAAAG ACTGACGCTC AGGTGCGAAA GCGTGGGGAG

Enterobacter_cloacae_(KP794925 GCAGGCGGTC TGTCAA-GTC GGATGTGAAA TCCCCGGGCT CAACCTGGGA ACTG-CATTC GAAACTGGCA GGCTGGAGTC TTGTAGAGGG GGGTAGAATT CCAGGTGTAG CGGTGAAATG CGTAGAGATC TGGAGGAATA CCGGTGGCGA AGGCGGCCCC CTGGACAAAG ACTGACGCTC AGGTGCGAAA GCGTGGGGAG

PaKu6_-_Staphylococcus_sciuri GTAGGCGGTT TCTTAA-GTC TGATGTGAAA GCCCACGGCT CAACCGTGGA GG-GTCATTG GAAACTGGGA AACTTGAGTG CAGAAGAGGA GAGTGGAATT CCATGTGTAG CGGTGAAATG CGCAGAGATA TGGAGGAACA CCAGTGGCGA AGGCGGCTCT CTGGTCTGTA ACTGACGCTG ATGTGCGAAA GCGTGGGGAT

Staphylococcus_sciuri_(MG70600 GTAGGCGGTT TCTTAA-GTC TGATGTGAAA GCCCACGGCT CAACCGTGGA GG-GTCATTG GAAACTGGGA AACTTGAGTG CAGAAGAGGA GAGTGGAATT CCATGTGTAG CGGTGAAATG CGCAGAGATA TGGAGGAACA CCAGTGGCGA AGGCGGCTCT CTGGTCTGTA ACTGACGCTG ATGTGCGAAA GCGTGGGGAT

Staphylococcus_lentus_(MG98829 GTAGGCGGTT TCTTAA-GTC TGATGTGAAA GCCCACGGCT CAACCGTGGA GG-GTCATTG GAAACTGGGA AACTTGAGTG CAGAAGAGGA GAGTGGAATT CCATGTGTAG CGGTGAAATG CGCAGAGATA TGGAGGAACA CCAGTGGCGA AGGCGGCTCT CTGGTCTGTA ACTGACGCTG ATGTGCGAAA GCGTGGGGAT

Staphylococcus_sciuri_(MG51742 GTAGGCGGTT TCTTAA-GTC TGATGTGAAA GCCCACGGCT CAACCGTGGA GG-GTCATTG GAAACTGGGA AACTTGAGTG CAGAAGAGGA GAGTGGAATT CCATGTGTAG CGGTGAAATG CGCAGAGATA TGGAGGAACA CCAGTGGCGA AGGCGGCTCT CTGGTCTGTA ACTGACGCTG ATGTGCGAAA GCGTGGGGAT

PaKu8_-_Pseudomonas_aeruginosa GTAGGTGGTT CAGCAA-GTT GGATGTGAAA TCCCCGGGCT CAACCTGGGA ACTG-CATCC AAAACTACTG AGCTAGAGTA CGGTAGAGGG TGGTGGAATT TCCTGTGTAG CGGTGAAATG CGTAGATATA GGAAGGAACA CCAGTGGCGA AGGCGACCAC CTGGACTGAT ACTGACACTG AGGTGCGAAA GCGTGGGGAG

Uncultured_bacterium_(FM996243 GTAGGTGGTT CAGCAA-GTT GGATGTGAAA TCCCCGGGCT CAACCTGGGA ACTG-CATCC AAAACTACTG AGCTAGAGTA CGGTAGAGGG TGGTGGAATT TCCTGTGTAG CGGTGAAATG CGTAGATATA GGAAGGAACA CCAGTGGCGA AGGCGACCAC CTGGACTGAT ACTGACACTG AGGTGCGAAA GCGTGGGGAG

Pseudomonas_aeruginosa_(MK4304 GTAGGTGGTT CAGCAA-GTT GGATGTGAAA TCCCCGGGCT CAACCTGGGA ACTG-CATCC AAAACTACTG AGCTAGAGTA CGGTAGAGGG TGGTGGAATT TCCTGTGTAG CGGTGAAATG CGTAGATATA GGAAGGAACA CCAGTGGCGA AGGCGACCAC CTGGACTGAT ACTGACACTG AGGTGCGAAA GCGTGGGGAG

PaKu9_-_Staphylococcus_arletta GTAGGCGGTT TCTTAA-GTC TGATGTGAAA GCCCACGGCT CAACCGTGGA GG-GTCATTG GAAACTGGGA GACTTGAGTG CAGAAGAGGA AAGTGGAATT CCATGTGTAG CGGTGAAATG CGCAGAGATA TGGAGGAACA CCAGTGGCGA AGGCGACTTT CTGGTCTGTA ACTGACGCTG ATGTGCGAAA GCGTGGGGAT

Staphylococcus_arlettae_(KX344 GTAGGCGGTT TCTTAA-GTC TGATGTGAAA GCCCACGGCT CAACCGTGGA GG-GTCATTG GAAACTGGGA GACTTGAGTG CAGAAGAGGA AAGTGGAATT CCATGTGTAG CGGTGAAATG CGCAGAGATA TGGAGGAACA CCAGTGGCGA AGGCGACTTT CTGGTCTGTA ACTGACGCTG ATGTGCGAAA GCGTGGGGAT

Staphylococcus_sp._(KU891836.1 GTAGGCGGTT TCTTAA-GTC TGATGTGAAA GCCCACGGCT CAACCGTGGA GG-GTCATTG GAAACTGGGA GACTTGAGTG CAGAAGAGGA AAGTGGAATT CCATGTGTAG CGGTGAAATG CGCAGAGATA TGGAGGAACA CCAGTGGCGA AGGCGACTTT CTGGTCTGTA ACTGACGCTG ATGTGCGAAA GCGTGGGGAT

PaKu10_-_Aeromonas_caviae GCAGGCGGTT GGATAA-GTT AGATGTGAAA GCCCCGGGCT CAACCTGGGA ATTG-CATTT AAAACTGTCC AGCTAGAGTC TTGTAGAGGG GGGTAGAATT CCAGGTGTAG CGGTGAAATG CGTAGAGATC TGGAGGAATA CCGGTGGCGA AGGCGGCCCC CTGGACAAAG ACTGACGCTC AGGTGCGAAA GCGTGGGGAG

Uncultured_bacterium_(KX242397 GCAGGCGGTT GGATAA-GTT AGATGTGAAA GCCCCGGGCT CAACCTGGGA ATTG-CATTT AAAACTGTCC AGCTAGAGTC TTGTAGAGGG GGGTAGAATT CCAGGTGTAG CGGTGAAATG CGTAGAGATC TGGAGGAATA CCGGTGGCGA AGGCGGCCCC CTGGACAAAG ACTGACGCTC AGGTGCGAAA GCGTGGGGAG

Aeromonas_caviae_(HQ407268.1) GCAGGCGGTT GGATAA-GTT AGATGTGAAA GCCCCGGGCT CAACCTGGGA ATTG-CATTT AAAACTGTCC AGCTAGAGTC TTGTAGAGGG GGGTAGAATT CCAGGTGTAG CGGTGAAATG CGTAGAGATC TGGAGGAATA CCGGTGGCGA AGGCGGCCCC CTGGACAAAG ACTGACGCTC AGGTGCGAAA GCGTGGGGAG

Aeromonas_aquariorum_(EU085557 GCAGGCGGTT GGATAA-GTT AGATGTGAAA GCCCCGGGCT CAACCTGGGA ATTG-CATTT AAAACTGTCC AGCTAGAGTC TTGTAGAGGG GGGTAGAATT CCAGGTGTAG CGGTGAAATG CGTAGAGATC TGGAGGAATA CCGGTGGCGA AGGCGGCCCC CTGGACAAAG ACTGACGCTC AGGTGCGAAA GCGTGGGGAG

PaKu11_-_Aeromonas_caviae GCAGGCGGTT GGATAA-GTT AGATGTGAAA GCCCCGGGCT CAACCTGGGA ATTG-CATTT AAAACTGTCC AGCTAGAGTC TTGTAGAGGG GGGTAGAATT CCAGGTGTAG CGGTGAAATG CGTAGAGATC TGGAGGAATA CCGGTGGCGA AGGCGGCCCC CTGGACAAAG ACTGACGCTC AGGTGCGAAA GCGTGGGGAG

Aeromonas_caviae_(KY087979.1) GCAGGCGGTT GGATAA-GTT AGATGTGAAA GCCCCGGGCT CAACCTGGGA ATTG-CATTT AAAACTGTCC AGCTAGAGTC TTGTAGAGGG GGGTAGAATT CCAGGTGTAG CGGTGAAATG CGTAGAGATC TGGAGGAATA CCGGTGGCGA AGGCGGCCCC CTGGACAAAG ACTGACGCTC AGGTGCGAAA GCGTGGGGAG

Uncultured_Aeromonas_sp._(KU94 GCAGGCGGTT GGATAA-GTT AGATGTGAAA GCCCCGGGCT CAACCTGGGA ATTG-CATTT AAAACTGTCC AGCTAGAGTC TTGTAGAGGG GGGTAGAATT CCAGGTGTAG CGGTGAAATG CGTAGAGATC TGGAGGAATA CCGGTGGCGA AGGCGGCCCC CTGGACAAAG ACTGACGCTC AGGTGCGAAA GCGTGGGGAG

PaKu12_-_Staphylococcus_warner GTAGGCGGTT TTTTAA-GTC TGATGTGAAA GCCCACGGCT CAACCGTGGA GG-GTCATTG GAAACTGGAA AACTTGAGTG CAGAAGAGGA AAGTGGAATT CCATGTGTAG CGGTGAAATG CGCAGAGATA TGGAGGAACA CCAGTGGCGA AGGCGACTTT CTGGTCTGTA ACTGACGCTG ATGTGCGAAA GCGTGGGGAT

Staphylococcus_warneri_(MH0149 GTAGGCGGTT TTTTAA-GTC TGATGTGAAA GCCCACGGCT CAACCGTGGA GG-GTCATTG GAAACTGGAA AACTTGAGTG CAGAAGAGGA AAGTGGAATT CCATGTGTAG CGGTGAAATG CGCAGAGATA TGGAGGAACA CCAGTGGCGA AGGCGACTTT CTGGTCTGTA ACTGACGCTG ATGTGCGAAA GCGTGGGGAT

Staphylococcus_warneri_(KF3063 GTAGGCGGTT TTTTAA-GTC TGATGTGAAA GCCCACGGCT CAACCGTGGA GG-GTCATTG GAAACTGGAA AACTTGAGTG CAGAAGAGGA AAGTGGAATT CCATGTGTAG CGGTGAAATG CGCAGAGATA TGGAGGAACA CCAGTGGCGA AGGCGACTTT CTGGTCTGTA ACTGACGCTG ATGTGCGAAA GCGTGGGGAT

PaKu13_-_Bacillus_megaterium GCAGGCGGTT TCTTAA-GTC TGATGTGAAA GCCCACGGCT CAACCGTGGA GG-GTCATTG GAAACTGGGG AACTTGAGTG CAGAAGAGAA AAGCGGAATT CCACGTGTAG CGGTGAAATG CGTAGAGATG TGGAGGAACA CCAGTGGCGA AGGCGGCTTT TTGGTCTGTA ACTGACGCTG AGGCGCGAAA GCGTGGGGAG

Bacillus_sp._(MK691444.1) GCAGGCGGTT TCTTAA-GTC TGATGTGAAA GCCCACGGCT CAACCGTGGA GG-GTCATTG GAAACTGGGG AACTTGAGTG CAGAAGAGAA AAGCGGAATT CCACGTGTAG CGGTGAAATG CGTAGAGATG TGGAGGAACA CCAGTGGCGA AGGCGGCTTT TTGGTCTGTA ACTGACGCTG AGGCGCGAAA GCGTGGGGAG

Bacillus_sp._(LC373523.1) GCAGGCGGTT TCTTAA-GTC TGATGTGAAA GCCCACGGCT CAACCGTGGA GG-GTCATTG GAAACTGGGG AACTTGAGTG CAGAAGAGAA AAGCGGAATT CCACGTGTAG CGGTGAAATG CGTAGAGATG TGGAGGAACA CCAGTGGCGA AGGCGGCTTT TTGGTCTGTA ACTGACGCTG AGGCGCGAAA GCGTGGGGAG

Bacillus_aryabhattai_(MK474942 GCAGGCGGTT TCTTAA-GTC TGATGTGAAA GCCCACGGCT CAACCGTGGA GG-GTCATTG GAAACTGGGG AACTTGAGTG CAGAAGAGAA AAGCGGAATT CCACGTGTAG CGGTGAAATG CGTAGAGATG TGGAGGAACA CCAGTGGCGA AGGCGGCTTT TTGGTCTGTA ACTGACGCTG AGGCGCGAAA GCGTGGGGAG

Bacillus_aryabhattai_(MK474941 GCAGGCGGTT TCTTAA-GTC TGATGTGAAA GCCCACGGCT CAACCGTGGA GG-GTCATTG GAAACTGGGG AACTTGAGTG CAGAAGAGAA AAGCGGAATT CCACGTGTAG CGGTGAAATG CGTAGAGATG TGGAGGAACA CCAGTGGCGA AGGCGGCTTT TTGGTCTGTA ACTGACGCTG AGGCGCGAAA GCGTGGGGAG

Bacillus_megaterium_(KX298860. GCAGGCGGTT TCTTAA-GTC TGATGTGAAA GCCCACGGCT CAACCGTGGA GG-GTCATTG GAAACTGGGG AACTTGAGTG CAGAAGAGAA AAGCGGAATT CCACGTGTAG CGGTGAAATG CGTAGAGATG TGGAGGAACA CCAGTGGCGA AGGCGGCTTT TTGGTCTGTA ACTGACGCTG AGGCGCGAAA GCGTGGGGAG

PaKu14_-_Pseudomonas_stutzeri GTAGGTGGTT CGTTAA-GTT GGATGTGAAA GCCCCGGGCT CAACCTGGGA ACTG-CATCC AAAACTGGCG AGCTAGAGTA TGGCAGAGGG TGGTGGAATT TCCTGTGTAG CGGTGAAATG CGTAGATATA GGAAGGAACA CCAGTGGCGA AGGCGACCAC CTGGGCTAAT ACTGACACTG AGGTGCGAAA GCGTGGGGAG

Pseudomonas_stutzeri_(MG892782 GTAGGTGGTT CGTTAA-GTT GGATGTGAAA GCCCCGGGCT CAACCTGGGA ACTG-CATCC AAAACTGGCG AGCTAGAGTA TGGCAGAGGG TGGTGGAATT TCCTGTGTAG CGGTGAAATG CGTAGATATA GGAAGGAACA CCAGTGGCGA AGGCGACCAC CTGGGCTAAT ACTGACACTG AGGTGCGAAA GCGTGGGGAG

Pseudomonas_stutzeri_(MG595371 GTAGGTGGTT CGTTAA-GTT GGATGTGAAA GCCCCGGGCT CAACCTGGGA ACTG-CATCC AAAACTGGCG AGCTAGAGTA TGGCAGAGGG TGGTGGAATT TCCTGTGTAG CGGTGAAATG CGTAGATATA GGAAGGAACA CCAGTGGCGA AGGCGACCAC CTGGGCTAAT ACTGACACTG AGGTGCGAAA GCGTGGGGAG

PaKu15_-_Rhizobium_sp. GTAGGCGGAT ATTTAA-GTC AGGGGTGAAA TCCCGCAGCT CAACTGCGGA ACTGCCTTT- GATACTGGGT ATCTTGAGTA TGGAAGAGGT AAGTGGAATT CCGAGTGTAG AGGTGAAATT CGTAGATATT CGGAGGAACA CCAGTGGCGA AGGCGGCTTA CTGGTCCATT ACTGACGCTG AGGTGCGAAA GCGTGGGGAG

Rhizobium_pusense_(CP039895.1) GTAGGCGGAT ATTTAA-GTC AGGGGTGAAA TCCCGCAGCT CAACTGCGGA ACTGCCTTT- GATACTGGGT ATCTTGAGTA TGGAAGAGGT AAGTGGAATT CCGAGTGTAG AGGTGAAATT CGTAGATATT CGGAGGAACA CCAGTGGCGA AGGCGGCTTA CTGGTCCATT ACTGACGCTG AGGTGCGAAA GCGTGGGGAG

Rhizobium_sp._(KY971009.1) GTAGGCGGAT ATTTAA-GTC AGGGGTGAAA TCCCGCAGCT CAACTGCGGA ACTGCCTTT- GATACTGGGT ATCTTGAGTA TGGAAGAGGT AAGTGGAATT CCGAGTGTAG AGGTGAAATT CGTAGATATT CGGAGGAACA CCAGTGGCGA AGGCGGCTTA CTGGTCCATT ACTGACGCTG AGGTGCGAAA GCGTGGGGAG

PaKu7_-_Stenotrophomonas_malto GTAGGTGGTC GTTTAA-GTC CGTTGTGAAA GCCCTGGGCT CAACCTGGGA ACTG-CAGTG GATACTGGGC GACTAGAATG TGGTAGAGGG TAGCGGAATT CCTGGTGTAG CAGTGAAATG CGTAGAGATC AGGAGGAACA TCCATGGCGA AGGCAGCTAC CTGGACCAAC ATTGACACTG AGGCACGAAA GCGTGGGGAG

Stenotrophomonas_maltophilia_( GTAGGTGGTC GTTTAA-GTC CGTTGTGAAA GCCCTGGGCT CAACCTGGGA ACTG-CAGTG GATACTGGGC GACTAGAATG TGGTAGAGGG TAGCGGAATT CCTGGTGTAG CAGTGAAATG CGTAGAGATC AGGAGGAACA TCCATGGCGA AGGCAGCTAC CTGGACCAAC ATTGACACTG AGGCACGAAA GCGTGGGGAG

Stenotrophomonas_maltophilia_( GTAGGTGGTC GTTTAA-GTC CGTTGTGAAA GCCCTGGGCT CAACCTGGGA ACTG-CAGTG GATACTGGGC GACTAGAATG TGGTAGAGGG TAGCGGAATT CCTGGTGTAG CAGTGAAATG CGTAGAGATC AGGAGGAACA TCCATGGCGA AGGCAGCTAC CTGGACCAAC ATTGACACTG AGGCACGAAA GCGTGGGGAG

PaKu16_-_Stenotrophomonas_malt GTAGGTGGTC GTTTAA-GTC CGTTGTGAAA GCCCTGGGCT CAACCTGGGA ACTG-CAGTG GATACTGGGC GACTAGAATG TGGTAGAGGG TAGCGGAATT CCTGGTGTAG CAGTGAAATG CGTAGAGATC AGGAGGAACA TCCATGGCGA AGGCAGCTAC CTGGACCAAC ATTGACACTG AGGCACGAAA GCGTGGGGAG

Stenotrophomonas_maltophilia_( GTAGGTGGTC GTTTAA-GTC CGTTGTGAAA GCCCTGGGCT CAACCTGGGA ACTG-CAGTG GATACTGGGC GACTAGAATG TGGTAGAGGG TAGCGGAATT CCTGGTGTAG CAGTGAAATG CGTAGAGATC AGGAGGAACA TCCATGGCGA AGGCAGCTAC CTGGACCAAC ATTGACACTG AGGCACGAAA GCGTGGGGAG

Stenotrophomonas_maltophilia_( GTAGGTGGTC GTTTAA-GTC CGTTGTGAAA GCCCTGGGCT CAACCTGGGA ACTG-CAGTG GATACTGGGC GACTAGAATG TGGTAGAGGG TAGCGGAATT CCTGGTGTAG CAGTGAAATG CGTAGAGATC AGGAGGAACA TCCATGGCGA AGGCAGCTAC CTGGACCAAC ATTGACACTG AGGCACGAAA GCGTGGGGAG

PaKu17_-_Stenotrophomonas_malt GTAGGTGGTC GTTTAA-GTC CGTTGTGAAA GCCCTGGGCT CAACCTGGGA ACTG-CAGTG GATACTGGGC GACTAGAATG TGGTAGAGGG TAGCGGAATT CCTGGTGTAG CAGTGAAATG CGTAGAGATC AGGAGGAACA TCCATGGCGA AGGCAGCTAC CTGGACCAAC ATTGACACTG AGGCACGAAA GCGTGGGGAG

PaKu26_-_Stenotrophomonas_malt GTAGGTGGTC GTTTAA-GTC CGTTGTGAAA GCCCTGGGCT CAACCTGGGA ACTG-CAGTG GATACTGGGC GACTAGAATG TGGTAGAGGG TAGCGGAATT CCTGGTGTAG CAGTGAAATG CGTAGAGATC AGGAGGAACA TCCATGGCGA AGGCAGCTAC CTGGACCAAC ATTGACACTG AGGCACGAAA GCGTGGGGAG

Stenotrophomonas_maltophilia_( GTAGGTGGTC GTTTAA-GTC CGTTGTGAAA GCCCTGGGCT CAACCTGGGA ACTG-CAGTG GATACTGGGC GACTAGAATG TGGTAGAGGG TAGCGGAATT CCTGGTGTAG CAGTGAAATG CGTAGAGATC AGGAGGAACA TCCATGGCGA AGGCAGCTAC CTGGACCAAC ATTGACACTG AGGCACGAAA GCGTGGGGAG

PaKu18_-_Stenotrophomonas_pana GTAGGTGGTG ATTTAA-GTC CGTTGTGAAA GCCCTGGGCT CAACCTGGGA ATTG-CAGTG GATACTGGGT CACTAGAGTG TGGTAGAGGG TAGCGGAATT CCCGGTGTAG CAGTGAAATG CGTAGAGATC GGGAGGAACA TCTGTGGCGA AGGCGGCTAC CTGGACCAAC ACTGACACTG AGGCACGAAA GCGTGGGGAG

Stenotrophomonas_panacihumi_(M GTAGGTGGTG ATTTAA-GTC CGTTGTGAAA GCCCTGGGCT CAACCTGGGA ATTG-CAGTG GATACTGGGT CACTAGAGTG TGGTAGAGGG TAGCGGAATT CCCGGTGTAG CAGTGAAATG CGTAGAGATC GGGAGGAACA TCTGTGGCGA AGGCGGCTAC CTGGACCAAC ACTGACACTG AGGCACGAAA GCGTGGGGAG

Stenotrophomonas_panacihumi_(K GTAGGTGGTG ATTTAA-GTC CGTTGTGAAA GCCCTGGGCT CAACCTGGGA ATTG-CAGTG GATACTGGGT CACTAGAGTG TGGTAGAGGG TAGCGGAATT CCCGGTGTAG CAGTGAAATG CGTAGAGATC GGGAGGAACA TCTGTGGCGA AGGCGGCTAC CTGGACCAAC ACTGACACTG AGGCACGAAA GCGTGGGGAG

PaKu19_-_Bacillus_licheniformi GCAGGCGGTT TCTTAA-GTC TGATGTGAAA GCCCCCGGCT CAACCGGGGA GG-GTCATTG GAAACTGGGG AACTTGAGTG CAGAAGAGGA GAGTGGAATT CCACGTGTAG CGGTGAAATG CGTAGAGATG TGGAGGAACA CCAGTGGCGA AGGCGACTCT CTGGTCTGTA ACTGACGCTG AGGCGCGAAA GCGTGGGGAG

Bacillus_licheniformis_(KJ5722 GCAGGCGGTT TCTTAA-GTC TGATGTGAAA GCCCCCGGCT CAACCGGGGA GG-GTCATTG GAAACTGGGG AACTTGAGTG CAGAAGAGGA GAGTGGAATT CCACGTGTAG CGGTGAAATG CGTAGAGATG TGGAGGAACA CCAGTGGCGA AGGCGACTCT CTGGTCTGTA ACTGACGCTG AGGCGCGAAA GCGTGGGGAG

Bacillus_licheniformis_(KT1536 GCAGGCGGTT TCTTAA-GTC TGATGTGAAA GCCCCCGGCT CAACCGGGGA GG-GTCATTG GAAACTGGGG AACTTGAGTG CAGAAGAGGA GAGTGGAATT CCACGTGTAG CGGTGAAATG CGTAGAGATG TGGAGGAACA CCAGTGGCGA AGGCGACTCT CTGGTCTGTA ACTGACGCTG AGGCGCGAAA GCGTGGGGAG

PaKu20_-__Pantoea_dispersa GCAGGCGGTC TGTTAA-GTC AGATGTGAAA TCCCCGGGCT TAACCTGGGA ACTG-CATTT GAAACTGGCA GGCTTGAGTC TCGTAGAGGG GGGTAGAATT CCAGGTGTAG CGGTGAAATG CGTAGAGATC TGGAGGAATA CCGGTGGCGA AGGCGGCCCC CTGGACGAAG ACTGACGCTC AGGTGCGAAA GCGTGGGGAG

Pantoea_dispersa_(KY292463.1) GCAGGCGGTC TGTTAA-GTC AGATGTGAAA TCCCCGGGCT TAACCTGGGA ACTG-CATTT GAAACTGGCA GGCTTGAGTC TCGTAGAGGG GGGTAGAATT CCAGGTGTAG CGGTGAAATG CGTAGAGATC TGGAGGAATA CCGGTGGCGA AGGCGGCCCC CTGGACGAAG ACTGACGCTC AGGTGCGAAA GCGTGGGGAG

Pantoea_dispersa_(KY882077.1) GCAGGCGGTC TGTTAA-GTC AGATGTGAAA TCCCCGGGCT TAACCTGGGA ACTG-CATTT GAAACTGGCA GGCTTGAGTC TCGTAGAGGG GGGTAGAATT CCAGGTGTAG CGGTGAAATG CGTAGAGATC TGGAGGAATA CCGGTGGCGA AGGCGGCCCC CTGGACGAAG ACTGACGCTC AGGTGCGAAA GCGTGGGGAG

PaKu21_-_Bacillus_sonorensis GCAGGCGGTT TCTTAA-GTC TGATGTGAAA GCCCCCGGCT CAACCGGGGA GG-GTCATTG GAAACTGGGG AACTTGAGTG CAGAAGAGGA GAGTGGAATT CCACGTGTAG CGGTGAAATG CGTAGAGATG TGGAGGAACA CCAGTGGCGA AGGCGACTCT CTGGTCTGTA ACTGACGCTG AGGCGCGAAA GCGTGGGGAG

Bacillus_sonorensis_(KU551167. GCAGGCGGTT TCTTAA-GTC TGATGTGAAA GCCCCCGGCT CAACCGGGGA GG-GTCATTG GAAACTGGGG AACTTGAGTG CAGAAGAGGA GAGTGGAATT CCACGTGTAG CGGTGAAATG CGTAGAGATG TGGAGGAACA CCAGTGGCGA AGGCGACTCT CTGGTCTGTA ACTGACGCTG AGGCGCGAAA GCGTGGGGAG

Bacillus_sonorensis_(FN397516. GCAGGCGGTT TCTTAA-GTC TGATGTGAAA GCCCCCGGCT CAACCGGGGA GG-GTCATTG GAAACTGGGG AACTTGAGTG CAGAAGAGGA GAGTGGAATT CCACGTGTAG CGGTGAAATG CGTAGAGATG TGGAGGAACA CCAGTGGCGA AGGCGACTCT CTGGTCTGTA ACTGACGCTG AGGCGCGAAA GCGTGGGGAG

Bacillus_sonorensis_(KU551137. GCAGGCGGTT TCTTAA-GTC TGATGTGAAA GCCCCCGGCT CAACCGGGGA GG-GTCATTG GAAACTGGGG AACTTGAGTG CAGAAGAGGA GAGTGGAATT CCACGTGTAG CGGTGAAATG CGTAGAGATG TGGAGGAACA CCAGTGGCGA AGGCGACTCT CTGGTCTGTA ACTGACGCTG AGGCGCGAAA GCGTGGGGAG

PaKu24_-_Bacillus_sonorensis GCAGGCGGTT TCTTAA-GTC TGATGTGAAA GCCCCCGGCT CAACCGGGGA GG-GTCATTG GAAACTGGGG AACTTGAGTG CAGAAGAGGA GAGTGGAATT CCACGTGTAG CGGTGAAATG CGTAGAGATG TGGAGGAACA CCAGTGGCGA AGGCGACTCT CTGGTCTGTA ACTGACGCTG AGGCGCGAAA GCGTGGGGAG

PaKu22_-_Bacillus_subtilis GCAGGCGGTT TCTTAA-GTC TGATGTGAAA GCCCCCGGCT CAACCGGGGA GG-GTCATTG GAAACTGGGG AACTTGAGTG CAGAAGAGGA GAGTGGAATT CCACGTGTAG CGGTGAAATG CGTAGAGATG TGGAGGAACA CCAGTGGCGA AGGCGACTCT CTGGTCTGTA ACTGACGCTG AGGAGCGAAA GCGTGGGGAG

Bacillus_tequilensis_(MK880583 GCAGGCGGTT TCTTAA-GTC TGATGTGAAA GCCCCCGGCT CAACCGGGGA GG-GTCATTG GAAACTGGGG AACTTGAGTG CAGAAGAGGA GAGTGGAATT CCACGTGTAG CGGTGAAATG CGTAGAGATG TGGAGGAACA CCAGTGGCGA AGGCGACTCT CTGGTCTGTA ACTGACGCTG AGGAGCGAAA GCGTGGGGAG

Bacillus_subtilis_(MK765023.1) GCAGGCGGTT TCTTAA-GTC TGATGTGAAA GCCCCCGGCT CAACCGGGGA GG-GTCATTG GAAACTGGGG AACTTGAGTG CAGAAGAGGA GAGTGGAATT CCACGTGTAG CGGTGAAATG CGTAGAGATG TGGAGGAACA CCAGTGGCGA AGGCGACTCT CTGGTCTGTA ACTGACGCTG AGGAGCGAAA GCGTGGGGAG

PaKu23_-_Bacillus_subtilis GCAGGCGGTT TCTTAA-GTC TGATGTGAAA GCCCCCGGCT CAACCGGGGA GG-GTCATTG GAAACTGGGG AACTTGAGTG CAGAAGAGGA GAGTGGAATT CCACGTGTAG CGGTGAAATG CGTAGAGATG TGGAGGAACA CCAGTGGCGA AGGCGACTCT CTGGTCTGTA ACTGACGCTG AGGAGCGAAA GCGTGGGGAG

Bacillus_subtilis_(MK511833.1) GCAGGCGGTT TCTTAA-GTC TGATGTGAAA GCCCCCGGCT CAACCGGGGA GG-GTCATTG GAAACTGGGG AACTTGAGTG CAGAAGAGGA GAGTGGAATT CCACGTGTAG CGGTGAAATG CGTAGAGATG TGGAGGAACA CCAGTGGCGA AGGCGACTCT CTGGTCTGTA ACTGACGCTG AGGAGCGAAA GCGTGGGGAG

Bacillus_subtilis_(KX450400.1) GCAGGCGGTT TCTTAA-GTC TGATGTGAAA GCCCCCGGCT CAACCGGGGA GG-GTCATTG GAAACTGGGG AACTTGAGTG CAGAAGAGGA GAGTGGAATT CCACGTGTAG CGGTGAAATG CGTAGAGATG TGGAGGAACA CCAGTGGCGA AGGCGACTCT CTGGTCTGTA ACTGACGCTG AGGAGCGAAA GCGTGGGGAG

PaKu25_-_Bacillus_cereus GCAGGTGGTT TCTTAA-GTC TGATGTGAAA GCCCACGGCT CAACCGTGGA GG-GTCATTG GAAACTGGGA GACTTGAGTG CAGAAGAGGA AAGTGGAATT CCATGTGTAG CGGTGAAATG CGTAGAGATA TGGAGGAACA CCAGTGGCGA AGGCGACTTT CTGGTCTGTA ACTGACACTG AGGCGCGAAA GCGTGGGGAG

Bacillus_thuringiensis_(CP0378 GCAGGTGGTT TCTTAA-GTC TGATGTGAAA GCCCACGGCT CAACCGTGGA GG-GTCATTG GAAACTGGGA GACTTGAGTG CAGAAGAGGA AAGTGGAATT CCATGTGTAG CGGTGAAATG CGTAGAGATA TGGAGGAACA CCAGTGGCGA AGGCGACTTT CTGGTCTGTA ACTGACACTG AGGCGCGAAA GCGTGGGGAG

Bacillus_mobilis_(CP031443.1) GCAGGTGGTT TCTTAA-GTC TGATGTGAAA GCCCACGGCT CAACCGTGGA GG-GTCATTG GAAACTGGGA GACTTGAGTG CAGAAGAGGA AAGTGGAATT CCATGTGTAG CGGTGAAATG CGTAGAGATA TGGAGGAACA CCAGTGGCGA AGGCGACTTT CTGGTCTGTA ACTGACACTG AGGCGCGAAA GCGTGGGGAG

Bacillus_thuringiensis_(CP0357 GCAGGTGGTT TCTTAA-GTC TGATGTGAAA GCCCACGGCT CAACCGTGGA GG-GTCATTG GAAACTGGGA GACTTGAGTG CAGAAGAGGA AAGTGGAATT CCATGTGTAG CGGTGAAATG CGTAGAGATA TGGAGGAACA CCAGTGGCGA AGGCGACTTT CTGGTCTGTA ACTGACACTG AGGCGCGAAA GCGTGGGGAG

Bacillus_cereus_(CP030982.1) GCAGGTGGTT TCTTAA-GTC TGATGTGAAA GCCCACGGCT CAACCGTGGA GG-GTCATTG GAAACTGGGA GACTTGAGTG CAGAAGAGGA AAGTGGAATT CCATGTGTAG CGGTGAAATG CGTAGAGATA TGGAGGAACA CCAGTGGCGA AGGCGACTTT CTGGTCTGTA ACTGACACTG AGGCGCGAAA GCGTGGGGAG

Bacillus_cereus_(MH068823.1) GCAGGTGGTT TCTTAA-GTC TGATGTGAAA GCCCACGGCT CAACCGTGGA GG-GTCATTG GAAACTGGGA GACTTGAGTG CAGAAGAGGA AAGTGGAATT CCATGTGTAG CGGTGAAATG CGTAGAGATA TGGAGGAACA CCAGTGGCGA AGGCGACTTT CTGGTCTGTA ACTGACACTG AGGCGCGAAA GCGTGGGGAG

Bacillus_thuringiensis_(MG7453 GCAGGTGGTT TCTTAA-GTC TGATGTGAAA GCCCACGGCT CAACCGTGGA GG-GTCATTG GAAACTGGGA GACTTGAGTG CAGAAGAGGA AAGTGGAATT CCATGTGTAG CGGTGAAATG CGTAGAGATA TGGAGGAACA CCAGTGGCGA AGGCGACTTT CTGGTCTGTA ACTGACACTG AGGCGCGAAA GCGTGGGGAG

Rhizobium_massiliae(Country-_T GTAGGCGGAT ATTTAA-GTC AGGGGTGAAA TCTCGCAGCT CAACTGCGGA ACTGCCTTTT GATACTGGGT ATCTTGAGTA TGGAAGAGGT AAGTGGAATT CCGAGTCTAG AGGTGAAATT CGTAGATATT CGGACGAACA CCAGTGGCGA AGGCGGCTTA CTGGTCCATT ACTGACGATG AGGTGCGAAA GCGTGGGGAG

Stenotrophomonas_maltophilia_( GTAGGTGGTC GTTTAAAGTC CGTTGTGAAA GCCCTGGGCT CAACCTGGGA ACTG-CAGTG GATACTGGGC GACTAGAGTG TGGTAGAGGG TAGCGGAATT CCTGGTGTAG CAGTGAAATG CGTAGAGATC AGGAGGAACA TCCATGGCGA AGGCAGCTAC CTGGACCAAC ACTGACACTG AGGCACGAAA GCGTGGGGAG

Serratia_sp._(Country-_Tunisia GCAGGTGGCT TGCCAA-GTC AGATGTGAAA TTCCCGGGCT CAACCCGGGA ATTG-CACTC GAAACTGGCA AGCCAGAGCC TCGTAGAGGG GGGTAGAATT CCAGGTGTAG CGGTGAAATG CGCAGAGACC CGGAGGAATA CCGGTGGCGA AGGCGGCCCC CTGGACGAAG ATTGATGTTC AGGTGCGAAA GCGTGGGGAG

Staphylococcus_saprophyticus_( GTAGGCGGTT TCTTAA-GTC TGATGTGAAA GCCCACGGCT CAACCGTGGA GG-GTCATTG GAAACTGGGA AACTTGAGTG CAGAAGAGGA AAGTGGAATT CCATGTGTAG CGGTGAAATG CGCAGAGATA TGGAGGAACA CCAGTGGCGA AGGCGACTTT CTGGTCTGTA ACTGACGCTG ATGTGCGAAA GCGTGGGGAT

Staphylococcus_saprophyticus_( GTAGGCGGTT TCTTAA-GTC TGATGTGAAA GCCCACGGCT CAACCGTGGA GG-GTCATTG GAAACTGGGA AACTTGAGTG CAGAAGAGGA AAGTGGAATT CCATGTGTAG CGGTGAAATG CGCAGAGATA TGGAGGAACA CCAGTGGCGA AGGCGACTTT CTGGTCTGTA ACTGACGCTG ATGTGCGAAA GCGTGGGGAT

Bacillus_subtilis_(Country-Ira GCAGGCGGTT TCTTAA-GTC TGATGTGAAA GCCCCCGGCT CAACCGGGGA GG-GTCATTG GAAACTGGGG AACTTGAGTG CAGAAGAGGA GAGTGGAATT CCACGTGTAG CGGTGAAATG CGTAGAGATG TGGAGGAACA CCAGTGGCGA AGGCGACTCT CTGGTCTGTA ACTGACGCTG AGGAGCGAAA GCGTGGGGAG

Bacillus_cereus_(Country-UK)_( GCAGGTGGTT TCTTAA-GTC TGATGTGAAA GCCCACGGCT CAACCGTGGA GG-GTCATTG GAAACTGGGA GACTTGAGTG CAGAAGAGGA AAGTGGAATT CCATGTGTAG CGGTGAAATG CGTAGAGATA TGGAGGAACA CCAGTGGCGA AG-CGACTTT CTGGTCTGTA ACTGACACTG AG-CGCGAAA GCGTGGGGAG

....|....| ....|....| ....|....| ....|....| ....|....| ....|....| ....|....| ....|....| ....|....| ....|....| ....|....| ....|....| ....|....| ....|....| ....|....| ....|....| ....|....| ....|....| ....|....| ....|....|

805 815 825 835 845 855 865 875 885 895 905 915 925 935 945 955 965 975 985 995

PaKu1_-_Serratia_marcescens CAAACAGGAT TAGATACCCT GGTAGTCCAC GCTGTAAACG ATGTCGATTT GGAGGTTGTG CCCTTGAGGC GTG-GCTTCC GGAGCTAACG CGTTAAATCG ACCGCCTGGG GAGTACGGCC GCAAGGTTAA AACTCAAATG AATTGACGGG GGCCCGCACA AGCGGTGGAG CATGTGGTTT AATTCGATGC AACGCGAAGA

Serratia_marcescens_(KX911721. CAAACAGGAT TAGATACCCT GGTAGTCCAC GCTGTAAACG ATGTCGATTT GGAGGTTGTG CCCTTGAGGC GTG-GCTTCC GGAGCTAACG CGTTAAATCG ACCGCCTGGG GAGTACGGCC GCAAGGTTAA AACTCAAATG AATTGACGGG GGCCCGCACA AGCGGTGGAG CATGTGGTTT AATTCGATGC AACGCGAAGA

Serratia_marcescens_(KT992361. CAAACAGGAT TAGATACCCT GGTAGTCCAC GCTGTAAACG ATGTCGATTT GGAGGTTGTG CCCTTGAGGC GTG-GCTTCC GGAGCTAACG CGTTAAATCG ACCGCCTGGG GAGTACGGCC GCAAGGTTAA AACTCAAATG AATTGACGGG GGCCCGCACA AGCGGTGGAG CATGTGGTTT AATTCGATGC AACGCGAAGA

PaKu2_-_Enterobacter_sp. CAAACAGGAT TAGATACCCT GGTAGTCCAC GCCGTAAACG ATGTCGACTT GGAGGTTGTG CCCTTGAGGC GTG-GCTTCC GGAGCTAACG CGTTAAGTCG ACCGCCTGGG GAGTACGGCC GCAAGGTTAA AACTCAAATG AATTGACGGG GGCCCGCACA AGCGGTGGAG CATGTGGTTT AATTCGATGC AACGCGAAGA

Enterobacter_sp._(MG280962.1) CAAACAGGAT TAGATACCCT GGTAGTCCAC GCCGTAAACG ATGTCGACTT GGAGGTTGTG CCCTTGAGGC GTG-GCTTCC GGAGCTAACG CGTTAAGTCG ACCGCCTGGG GAGTACGGCC GCAAGGTTAA AACTCAAATG AATTGACGGG GGCCCGCACA AGCGGTGGAG CATGTGGTTT AATTCGATGC AACGCGAAGA

Enterobacter_hormaechei_(CP029 CAAACAGGAT TAGATACCCT GGTAGTCCAC GCCGTAAACG ATGTCGACTT GGAGGTTGTG CCCTTGAGGC GTG-GCTTCC GGAGCTAACG CGTTAAGTCG ACCGCCTGGG GAGTACGGCC GCAAGGTTAA AACTCAAATG AATTGACGGG GGCCCGCACA AGCGGTGGAG CATGTGGTTT AATTCGATGC AACGCGAAGA

PaKu3_-_Staphylococcus_saproph CAAACAGGAT TAGATACCCT GGTAGTCCAC GCCGTAAACG ATGAGTGCTA AGTGTTAGGG GGTTTCCGCC CCTTAGTGCT GCAGCTAACG CATTAAGCAC TCCGCCTGGG GAGTACGACC GCAAGGTTGA AACTCAAAGG AATTGACGGG GACCCGCACA AGCGGTGGAG CATGTGGTTT AATTCGAAGC AACGCGAAGA

Staphylococcus_saprophyticus_( CAAACAGGAT TAGATACCCT GGTAGTCCAC GCCGTAAACG ATGAGTGCTA AGTGTTAGGG GGTTTCCGCC CCTTAGTGCT GCAGCTAACG CATTAAGCAC TCCGCCTGGG GAGTACGACC GCAAGGTTGA AACTCAAAGG AATTGACGGG GACCCGCACA AGCGGTGGAG CATGTGGTTT AATTCGAAGC AACGCGAAGA

Staphylococcus_saprophyticus_( CAAACAGGAT TAGATACCCT GGTAGTCCAC GCCGTAAACG ATGAGTGCTA AGTGTTAGGG GGTTTCCGCC CCTTAGTGCT GCAGCTAACG CATTAAGCAC TCCGCCTGGG GAGTACGACC GCAAGGTTGA AACTCAAAGG AATTGACGGG GACCCGCACA AGCGGTGGAG CATGTGGTTT AATTCGAAGC AACGCGAAGA

PaKu4_-_Enterobacter_cloacae CAAACAGGAT TAGATACCCT GGTAGTCCAC GCCGTAAACG ATGTCGATTT GGAGGTTGTG CCCTTGAGGC GTG-GCTTCC GGAGCTAACG CGTTAAATCG ACCGCCTGGG GAGTACGGCC GCAAGGTTAA AACTCAAATG AATTGACGGG GGCCCGCACA AGCGGTGGAG CATGTGGTTT AATTCGATGC AACGCGAAGA

Uncultured_Enterobacter_sp._(M CAAACAGGAT TAGATACCCT GGTAGTCCAC GCCGTAAACG ATGTCGATTT GGAGGTTGTG CCCTTGAGGC GTG-GCTTCC GGAGCTAACG CGTTAAATCG ACCGCCTGGG GAGTACGGCC GCAAGGTTAA AACTCAAATG AATTGACGGG GGCCCGCACA AGCGGTGGAG CATGTGGTTT AATTCGATGC AACGCGAAGA

Enterobacter_cloacae_(KY930709 CAAACAGGAT TAGATACCCT GGTAGTCCAC GCCGTAAACG ATGTCGATTT GGAGGTTGTG CCCTTGAGGC GTG-GCTTCC GGAGCTAACG CGTTAAATCG ACCGCCTGGG GAGTACGGCC GCAAGGTTAA AACTCAAATG AATTGACGGG GGCCCGCACA AGCGGTGGAG CATGTGGTTT AATTCGATGC AACGCGAAGA

PaKu5_-_Enterobacter_cloacae CAAACAGGAT TAGATACCCT GGTAGTCCAC GCCGTAAACG ATGTCGATTT GGAGGTTGTG CCCTTGAGGC GTG-GCTTCC GGAGCTAACG CGTTAAATCG ACCGCCTGGG GAGTACGGCC GCAAGGTTAA AACTCAAATG AATTGACGGG GGCCCGCACA AGCGGTGGAG CATGTGGTTT AATTCGATGC AACGCGAAGA

Enterobacter_sp._(KU867838.1) CAAACAGGAT TAGATACCCT GGTAGTCCAC GCCGTAAACG ATGTCGATTT GGAGGTTGTG CCCTTGAGGC GTG-GCTTCC GGAGCTAACG CGTTAAATCG ACCGCCTGGG GAGTACGGCC GCAAGGTTAA AACTCAAATG AATTGACGGG GGCCCGCACA AGCGGTGGAG CATGTGGTTT AATTCGATGC AACGCGAAGA

Enterobacter_cloacae_(KX242265 CAAACAGGAT TAGATACCCT GGTAGTCCAC GCCGTAAACG ATGTCGATTT GGAGGTTGTG CCCTTGAGGC GTG-GCTTCC GGAGCTAACG CGTTAAATCG ACCGCCTGGG GAGTACGGCC GCAAGGTTAA AACTCAAATG AATTGACGGG GGCCCGCACA AGCGGTGGAG CATGTGGTTT AATTCGATGC AACGCGAAGA

Enterobacter_cloacae_(KP794925 CAAACAGGAT TAGATACCCT GGTAGTCCAC GCCGTAAACG ATGTCGATTT GGAGGTTGTG CCCTTGAGGC GTG-GCTTCC GGAGCTAACG CGTTAAATCG ACCGCCTGGG GAGTACGGCC GCAAGGTTAA AACTCAAATG AATTGACGGG GGCCCGCACA AGCGGTGGAG CATGTGGTTT AATTCGATGC AACGCGAAAA

PaKu6_-_Staphylococcus_sciuri CAAACAGGAT TAGATACCCT GGTAGTCCAC GCCGTAAACG ATGAGTGCTA AGTGTTAGGG GGTTTCCGCC CCTTAGTGCT GCAGCTAACG CATTAAGCAC TCCGCCTGGG GAGTACGACC GCAAGGTTGA AACTCAAAGG AATTGACGGG GACCCGCACA AGCGGTGGAG CATGTGGTTT AATTCGAAGC AACGCGAAGA

Staphylococcus_sciuri_(MG70600 CAAACAGGAT TAGATACCCT GGTAGTCCAC GCCGTAAACG ATGAGTGCTA AGTGTTAGGG GGTTTCCGCC CCTTAGTGCT GCAGCTAACG CATTAAGCAC TCCGCCTGGG GAGTACGACC GCAAGGTTGA AACTCAAAGG AATTGACGGG GACCCGCACA AGCGGTGGAG CATGTGGTTT AATTCGAAGC AACGCGAAGA

Staphylococcus_lentus_(MG98829 CAAACAGGAT TAGATACCCT GGTAGTCCAC GCCGTAAACG ATGAGTGCTA AGTGTTAGGG GGTTTCCGCC CCTTAGTGCT GCAGCTAACG CATTAAGCAC TCCGCCTGGG GAGTACGACC GCAAGGTTGA AACTCAAAGG AATTGACGGG GACCCGCACA AGCGGTGGAG CATGTGGTTT AATTCGAAGC AACGCGAAGA

Staphylococcus_sciuri_(MG51742 CAAACAGGAT TAGATACCCT GGTAGTCCAC GCCGTAAACG ATGAGTGCTA AGTGTTAGGG GGTTTCCGCC CCTTAGTGCT GCAGCTAACG CATTAAGCAC TCCGCCTGGG GAGTACGACC GCAAGGTTGA AACTCAAAGG AATTGACGGG GACCCGCACA AGCGGTGGAG CATGTGGTTT AATTCGAAGC AACGCGAAGA

PaKu8_-_Pseudomonas_aeruginosa CAAACAGGAT TAGATACCCT GGTAGTCCAC GCCGTAAACG ATGTCGACTA GCCGTTGGGA TCCTTGAGAT CTT-AGTGGC GCAGCTAACG CGATAAGTCG ACCGCCTGGG GAGTACGGCC GCAAGGTTAA AACTCAAATG AATTGACGGG GGCCCGCACA AGCGGTGGAG CATGTGGTTT AATTCGAAGC AACGCGAAGA

Uncultured_bacterium_(FM996243 CAAACAGGAT TAGATACCCT GGTAGTCCAC GCCGTAAACG ATGTCGACTA GCCGTTGGGG TCCTTGAGAT CTT-AGTGGC GCAGCTAACG CGATAAGTCG ACCGCCTGGG GAGTACGGCC GCAAGGTTAA AACTCAAATG AATTGACGGG GGCCCGCACA AGCGGTGGAG CATGTGGTTT AATTCGAAGC AACGCGAAGA

Pseudomonas_aeruginosa_(MK4304 CAAACAGGAT TAGATACCCT GGTAGTCCAC GCCGTAAACG ATGTCGACTA GCCGTTGGGA TCCTTGAGAT CTT-AGTGGC GCAGCTAACG CGATAAGTCG ACCGCCTGGG GAGTACGGCC GCAAGGTTAA AACTCAAATG AATTGACGGG GGCCCGCACA AGCGGTGGAG CATGTGGTTT AATTCGAAGC AACGCGAAGA

PaKu9_-_Staphylococcus_arletta CAAACAGGAT TAGATACCCT GGTAGTCCAC GCCGTAAACG ATGAGTGCTA AGTGTTAGGG GGTTTCCGCC CCTTAGTGCT GCAGCTAACG CATTAAGCAC TCCGCCTGGG GAGTACGACC GCAAGGTTGA AACTCAAAGG AATTGACGGG GACCCGCACA AGCGGTGGAG CATGTGGTTT AATTCGAAGC AACGCGAAGA

Staphylococcus_arlettae_(KX344 CAAACAGGAT TAGATACCCT GGTAGTCCAC GCCGTAAACG ATGAGTGCTA AGTGTTAGGG GGTTTCCGCC CCTTAGTGCT GCAGCTAACG CATTAAGCAC TCCGCCTGGG GAGTACGACC GCAAGGTTGA AACTCAAAGG AATTGACGGG GACCCGCACA AGCGGTGGAG CATGTGGTTT AATTCGAAGC AACGCGAAGA

Staphylococcus_sp._(KU891836.1 CAAACAGGAT TAGATACCCT GGTAGTCCAC GCCGTAAACG ATGAGTGCTA AGTGTTAGGG GGTTTCCGCC CCTTAGTGCT GCAGCTAACG CATTAAGCAC TCCGCCTGGG GAGTACGACC GCAAGGTTGA AACTCAAAGG AATTGACGGG GACCCGCACA AGCGGTGGAG CATGTGGTTT AATTCGAAGC AACGCGAAGA

PaKu10_-_Aeromonas_caviae CAAACAGGAT TAGATACCCT GGTAGTCCAC GCCGTAAACG ATGTCGATTT GGAGGCTGTG TCCTTGAGAC GTG-GCTTCC GGAGCTAACG CGTTAAATCG ACCGCCTGGG GAGTACGGCC GCAAGGTTAA AACTCAAATG AATTGACGGG GGCCCGCACA AGCGGTGGAG CATGTGGTTT AATTCGATGC AACGCGAAGA

Uncultured_bacterium_(KX242397 CAAACAGGAT TAGATACCCT GGTAGTCCAC GCCGTAAACG ATGTCGATTT GGAGGCTGTG TCCTTGAGAC GTG-GCTTCC GGAGCTAACG CGTTAAATCG ACCGCCTGGG GAGTACGGCC GCAAGGTTAA AACTCAAATG AATTGACGGG GGCCCGCACA AGCGGTGGAG CATGTGGTTT AATTCGATGC AACGCGAAGA

Aeromonas_caviae_(HQ407268.1) CAAACAGGAT TAGATACCCT GGTAGTCCAC GCCGTAAACG ATGTCGATTT GGAGGCTGTG TCCTTGAGAC GTG-GCTTCC GGAGCTAACG CGTTAAATCG ACCGCCTGGG GAGTACGGCC GCAAGGTTAA AACTCAAATG AATTGACGGG GGCCCGCACA AGCGGTGGAG CATGTGGTTT AATTCGATGC AACGCGAAGA

Aeromonas_aquariorum_(EU085557 CAAACAGGAT TAGATACCCT GGTAGTCCAC GCCGTAAACG ATGTCGATTT GGAGGCTGTG TCCTTGAGAC GTG-GCTTCC GGAGCTAACG CGTTAAATCG ACCGCCTGGG GAGTACGGCC GCAAGGTTAA AACTCAAATG AATTGACGGG GGCCCGCACA AGCGGTGGAG CATGTGGTTT AATTCGATGC AACGCGAAGA

PaKu11_-_Aeromonas_caviae CAAACAGGAT TAGATACCCT GGTAGTCCAC GCCGTAAACG ATGTCGATTT GGAGGCTGTG TCCTTGAGAC GTG-GCTTCC GGAGCTAACG CGTTAAATCG ACCGCCTGGG GAGTACGGCC GCAAGGTTAA AACTCAAATG AATTGACGGG GGCCCGCACA AGCGGTGGAG CATGTGGTTT AATTCGATGC AACGCGAAGA

Aeromonas_caviae_(KY087979.1) CAAACAGGAT TAGATACCCT GGTAGTCCAC GCCGTAAACG ATGTCGATTT GGAGGCTGTG TCCTTGAGAC GTG-GCTTCC GGAGCTAACG CGTTAAATCG ACCGCCTGGG GAGTACGGCC GCAAGGTTAA AACTCAAATG AATTGACGGG GGCCCGCACA AGCGGTGGAG CATGTGGTTT AATTCGATGC AACGCGAAGA

Uncultured_Aeromonas_sp._(KU94 CAAACAGGAT TAGATACCCT GGTAGTCCAC GCCGTAAACG ATGTCGATTT GGAGGCTGTG TCCTTGAGAC GTG-GCTTCC GGAGCTAACG CGTTAAATCG ACCGCCTGGG GAGTACGGCC GCAAGGTTAA AACTCAAATG AATTGACGGG GGCCCGCACA AGCGGTGGAG CATGTGGTTT AATTCGATGC AACGCGAAGA

PaKu12_-_Staphylococcus_warner CAAACAGGAT TAGATACCCT GGTAGTCCAC GCCGTAAACG ATGAGTGCTA AGTGTTAGGG GGTTTCCGCC CCTTAGTGCT GCAGCTAACG CATTAAGCAC TCCGCCTGGG GAGTACGACC GCAAGGTTGA AACTCAAAGG AATTGACGGG GACCCGCACA AGCGGTGGAG CATGTGGTTT AATTCGAAGC AACGCGAAGA

Staphylococcus_warneri_(MH0149 CAAACAGGAT TAGATACCCT GGTAGTCCAC GCCGTAAACG ATGAGTGCTA AGTGTTAGGG GGTTTCCGCC CCTTAGTGCT GCAGCTAACG CATTAAGCAC TCCGCCTGGG GAGTACGACC GCAAGGTTGA AACTCAAAGG AATTGACGGG GACCCGCACA AGCGGTGGAG CATGTGGTTT AATTCGAAGC AACGCGAAGA

Staphylococcus_warneri_(KF3063 CAAACAGGAT TAGATACCCT GGTAGTCCAC GCCGTAAACG ATGAGTGCTA AGTGTTAGGG GGTTTCCGCC CCTTAGTGCT GCAGCTAACG CATTAAGCAC TCCGCCTGGG GAGTACGACC GCAAGGTTGA AACTCAAAGG AATTGACGGG GACCCGCACA AGCGGTGGAG CATGTGGTTT AATTCGAAGC AACGCGAAGA

PaKu13_-_Bacillus_megaterium CAAACAGGAT TAGATACCCT GGTAGTCCAC GCCGTAAACG ATGAGTGCTA AGTGTTAGAG GGTTTCCGCC CTTTAGTGCT GCAGCTAACG CATTAAGCAC TCCGCCTGGG GAGTACGGTC GCAAGACTGA AACTCAAAGG AATTGACGGG GGCCCGCACA AGCGGTGGAG CATGTGGTTT AATTCGAAGC AACGCGAAGA

Bacillus_sp._(MK691444.1) CAAACAGGAT TAGATACCCT GGTAGTCCAC GCCGTAAACG ATGAGTGCTA AGTGTTAGAG GGTTTCCGCC CTTTAGTGCT GCAGCTAACG CATTAAGCAC TCCGCCTGGG GAGTACGGTC GCAAGACTGA AACTCAAAGG AATTGACGGG GGCCCGCACA AGCGGTGGAG CATGTGGTTT AATTCGAAGC AACGCGAAGA

Bacillus_sp._(LC373523.1) CAAACAGGAT TAGATACCCT GGTAGTCCAC GCCGTAAACG ATGAGTGCTA AGTGTTAGAG GGTTTCCGCC CTTTAGTGCT GCAGCTAACG CATTAAGCAC TCCGCCTGGG GAGTACGGTC GCAAGACTGA AACTCAAAGG AATTGACGGG GGCCCGCACA AGCGGTGGAG CATGTGGTTT AATTCGAAGC AACGCGAAGA

Bacillus_aryabhattai_(MK474942 CAAACAGGAT TAGATACCCT GGTAGTCCAC GCCGTAAACG ATGAGTGCTA AGTGTTAGAG GGTTTCCGCC CTTTAGTGCT GCAGCTAACG CATTAAGCAC TCCGCCTGGG GAGTACGGTC GCAAGACTGA AACTCAAAGG AATTGACGGG GGCCCGCACA AGCGGTGGAG CATGTGGTTT AATTCGAAGC AACGCGAAGA

Bacillus_aryabhattai_(MK474941 CAAACAGGAT TAGATACCCT GGTAGTCCAC GCCGTAAACG ATGAGTGCTA AGTGTTAGAG GGTTTCCGCC CTTTAGTGCT GCAGCTAACG CATTAAGCAC TCCGCCTGGG GAGTACGGTC GCAAGACTGA AACTCAAAGG AATTGACGGG GGCCCGCACA AGCGGTGGAG CATGTGGTTT AATTCGAAGC AACGCGAAGA

Bacillus_megaterium_(KX298860. CAAACAGGAT TAGATACCCT GGTAGTCCAC GCCGTAAACG ATGAGTGCTA AGTGTTAGAG GGTTTCCGCC CTTTAGTGCT GCAGCTAACG CATTAAGCAC TCCGCCTGGG GAGTACGGTC GCAAGACTGA AACTCAAAGG AATTGACGGG GGCCCGCACA AGCGGTGGAG CATGTGGTTT AATTCGAAGC AACGCGAAGA

PaKu14_-_Pseudomonas_stutzeri CAAACAGGAT TAGATACCCT GGTAGTCCAC GCCGTAAACG ATGTCGACTA GCCGTTGGGA TCCTTGAGAT CTT-AGTGGC GCAGCTAACG CATTAAGTCG ACCGCCTGGG GAGTACGGCC GCAAGGTTAA AACTCAAATG AATTGACGGG GGCCCGCACA AGCGGTGGAG CATGTGGTTT AATTCGAAGC AACGCGAAGA

Pseudomonas_stutzeri_(MG892782 CAAACAGGAT TAGATACCCT GGTAGTCCAC GCCGTAAACG ATGTCGACTA GCCGTTGGGA TCCTTGAGAT CTT-AGTGGC GCAGCTAACG CATTAAGTCG ACCGCCTGGG GAGTACGGCC GCAAGGTTAA AACTCAAATG AATTGACGGG GGCCCGCACA AGCGGTGGAG CATGTGGTTT AATTCGAAGC AACGCGAAGA

Pseudomonas_stutzeri_(MG595371 CAAACAGGAT TAGATACCCT GGTAGTCCAC GCCGTAAACG ATGTCGACTA GCCGTTGGGA TCCTTGAGAT CTT-AGTGGC GCAGCTAACG CATTAAGTCG ACCGCCTGGG GAGTACGGCC GCAAGGTTAA AACTCAAATG AATTGACGGG GGCCCGCACA AGCGGTGGAG CATGTGGTTT AATTCGAAGC AACGCGAAGA

PaKu15_-_Rhizobium_sp. CAAACAGGAT TAGATACCCT GGTAGTCCAC GCCGTAAACG ATGAATGTTA GCCGTCGGGC AGTAT--ACT GTTCGGTGGC GCAGCTAACG CATTAAACAT TCCGCCTGGG GAGTACGGTC GCAAGATTAA AACTCAAAGG AATTGACGGG GGCCCGCACA AGCGGTGGAG CATGTGGTTT AATTCGAAGC AACGCGCAGA

Rhizobium_pusense_(CP039895.1) CAAACAGGAT TAGATACCCT GGTAGTCCAC GCCGTAAACG ATGAATGTTA GCCGTCGGGC AGTAT--ACT GTTCGGTGGC GCAGCTAACG CATTAAACAT TCCGCCTGGG GAGTACGGTC GCAAGATTAA AACTCAAAGG AATTGACGGG GGCCCGCACA AGCGGTGGAG CATGTGGTTT AATTCGAAGC AACGCGCAGA

Rhizobium_sp._(KY971009.1) CAAACAGGAT TAGATACCCT GGTAGTCCAC GCCGTAAACG ATGAATGTTA GCCGTCGGGC AGTAT--ACT GTTCGGTGGC GCAGCTAACG CATTAAACAT TCCGCCTGGG GAGTACGGTC GCAAGATTAA AACTCAAAGG AATTGACGGG GGCCCGCACA AGCGGTGGAG CATGTGGTTT AATTCGAAGC AACGCGCAGA

PaKu7_-_Stenotrophomonas_malto CAAACAGGAT TAGATACCCT GGTAGTCCAC GCCCTAAACG ATGCGAACTG GATGTTGGGT GCAATTTGGC ACGCAGTATC GAAGCTAACG CGTTAAGTTC GCCGCCTGGG GAGTACGGTC GCAAGACTGA AACTCAAAGG AATTGACGGG GGCCCGCACA AGCGGTGGAG TATGTGGTTT AATTCGATGC AACGCGAAGA

Stenotrophomonas_maltophilia_( CAAACAGGAT TAGATACCCT GGTAGTCCAC GCCCTAAACG ATGCGAACTG GATGTTGGGT GCAATTTGGC ACGCAGTATC GAAGCTAACG CGTTAAGTTC GCCGCCTGGG GAGTACGGTC GCAAGACTGA AACTCAAAGG AATTGACGGG GGCCCGCACA AGCGGTGGAG TATGTGGTTT AATTCGATGC AACGCGAAGA

Stenotrophomonas_maltophilia_( CAAACAGGAT TAGATACCCT GGTAGTCCAC GCCCTAAACG ATGCGAACTG GATGTTGGGT GCAATTTGGC ACGCAGTATC GAAGCTAACG CGTTAAGTTC GCCGCCTGGG GAGTACGGTC GCAAGACTGA AACTCAAAGG AATTGACGGG GGCCCGCACA AGCGGTGGAG TATGTGGTTT AATTCGATGC AACGCGAAGA

PaKu16_-_Stenotrophomonas_malt CAAACAGGAT TAGATACCCT GGTAGTCCAC GCCCTAAACG ATGCGAACTG GATGTTGGGT GCAATTTGGC ACGCAGTATC GAAGCTAACG CGTTAAGTTC GCCGCCTGGG GAGTACGGTC GCAAGACTGA AACTCAAAGG AATTGACGGG GGCCCGCACA AGCGGTGGAG TATGTGGTTT AATTCGATGC AACGCGAAGA

Stenotrophomonas_maltophilia_( CAAACAGGAT TAGATACCCT GGTAGTCCAC GCCCTAAACG ATGCGAACTG GATGTTGGGT GCAATTTGGC ACGCAGTATC GAAGCTAACG CGTTAAGTTC GCCGCCTGGG GAGTACGGTC GCAAGACTGA AACTCAAAGG AATTGACGGG GGCCCGCACA AGCGGTGGAG TATGTGGTTT AATTCGATGC AACGCGAAGA

Stenotrophomonas_maltophilia_( CAAACAGGAT TAGATACCCT GGTAGTCCAC GCCCTAAACG ATGCGAACTG GATGTTGGGT GCAATTTGGC ACGCAGTATC GAAGCTAACG CGTTAAGTTC GCCGCCTGGG GAGTACGGTC GCAAGACTGA AACTCAAAGG AATTGACGGG GGCCCGCACA AGCGGTGGAG TATGTGGTTT AATTCGATGC AACGCGAAGA

PaKu17_-_Stenotrophomonas_malt CAAACAGGAT TAGATACCCT GGTAGTCCAC GCCCTAAACG ATGCGAACTG GATGTTGGGT GCAATTTGGC ACGCAGTATC GAAGCTAACG CGTTAAGTTC GCCGCCTGGG GAGTACGGTC GCAAGACTGA AACTCAAAGG AATTGACGGG GGCCCGCACA AGCGGTGGAG TATGTGGTTT AATTCGATGC AACGCGAAGA

PaKu26_-_Stenotrophomonas_malt CAAACAGGAT TAGATACCCT GGTAGTCCAC GCCCTAAACG ATGCGAACTG GATGTTGGGT GCAATTTGGC ACGCAGTATC GAAGCTAACG CGTTAAGTTC GCCGCCTGGG GAGTACGGTC GCAAGACTGA AACTCAAAGG AATTGACGGG GGCCCGCACA AGCGGTGGAG TATGTGGTTT AATTCGATGC AACGCGAAGA

Stenotrophomonas_maltophilia_( CAAACAGGAT TAGATACCCT GGTAGTCCAC GCCCTAAACG ATGCGAACTG GATGTTGGGT GCAATTTGGC ACGCAGTATC GAAGCTAACG CGTTAAGTTC GCCGCCTGGG GAGTACGGTC GCAAGACTGA AACTCAAAGG AATTGACGGG GGCCCGCACA AGCGGTGGAG TATGTGGTTT AATTCGATGC AACGCGAAGA

PaKu18_-_Stenotrophomonas_pana CAAACAGGAT TAGATACCCT GGTAGTCCAC GCCCTAAACG ATGCGAACTG GATGTTGGGT GCAATTTGGC ACGCAGTATC GAAGCTAACG CGTTAAGTTC GCCGCCTGGG GAGTACGGTC GCAAGACTGA AACTCAAAGG AATTGACGGG GGCCCGCACA AGCGGTGGAG TATGTGGTTT AATTCGATGC AACGCGAAGA

Stenotrophomonas_panacihumi_(M CAAACAGGAT TAGATACCCT GGTAGTCCAC GCCCTAAACG ATGCGAACTG GATGTTGGGT GCAATTTGGC ACGCAGTATC GAAGCTAACG CGTTAAGTTC GCCGCCTGGG GAGTACGGTC GCAAGACTGA AACTCAAAGG AATTGACGGG GGCCCGCACA AGCGGTGGAG TATGTGGTTT AATTCGATGC AACGCGAAGA

Stenotrophomonas_panacihumi_(K CAAACAGGAT TAGATACCCT GGTAGTCCAC GCCCTAAACG ATGCGAACTG GATGTTGGGT GCAATTTGGC ACGCAGTATC GAAGCTAACG CGTTAAGTTC GCCGCCTGGG GAGTACGGTC GCAAGACTGA AACTCAAAGG AATTGACGGG GGCCCGCACA AGCGGTGGAG TATGTGGTTT AATTCGATGC AACGCGAAGA

PaKu19_-_Bacillus_licheniformi CGAACAGGAT TAGATACCCT GGTAGTCCAC GCCGTAAACG ATGAGTGCTA AGTGTTAGAG GGTTTCCGCC CTTTAGTGCT GCAGCAAACG CATTAAGCAC TCCGCCTGGG GAGTACGGTC GCAAGACTGA AACTCAAAGG AATTGACGGG GGCCCGCACA AGCGGTGGAG CATGTGGTTT AATTCGAAGC AACGCGAAGA

Bacillus_licheniformis_(KJ5722 CGAACAGGAT TAGATACCCT GGTAGTCCAC GCCGTAAACG ATGAGTGCTA AGTGTTAGAG GGTTTCCGCC CTTTAGTGCT GCAGCAAACG CATTAAGCAC TCCGCCTGGG GAGTACGGTC GCAAGACTGA AACTCAAAGG AATTGACGGG GGCCCGCACA AGCGGTGGAG CATGTGGTTT AATTCGAAGC AACGCGAAGA

Bacillus_licheniformis_(KT1536 CGAACAGGAT TAGATACCCT GGTAGTCCAC GCCGTAAACG ATGAGTGCTA AGTGTTAGAG GGTTTCCGCC CTTTAGTGCT GCAGCAAACG CATTAAGCAC TCCGCCTGGG GAGTACGGTC GCAAGACTGA AACTCAAAGG AATTGACGGG GGCCCGCACA AGCGGTGGAG CATGTGGTTT AATTCGAAGC AACGCGAAGA

PaKu20_-__Pantoea_dispersa CAAACAGGAT TAGATACCCT GGTAGTCCAC GCCGTAAACG ATGTCGACTT GGAGGTTGTG CCCTTGAGGC GTG-GCTTCC GGAGCTAACG CGTTAAGTCG ACCGCCTGGG GAGTACGGCC GCAAGGTTAA AACTCAAATG AATTGACGGG GGCCCGCACA AGCGGTGGAG CATGTGGTTT AATTCGATGC AACGCGAAGA

Pantoea_dispersa_(KY292463.1) CAAACAGGAT TAGATACCCT GGTAGTCCAC GCCGTAAACG ATGTCGACTT GGAGGTTGTG CCCTTGAGGC GTG-GCTTCC GGAGCTAACG CGTTAAGTCG ACCGCCTGGG GAGTACGGCC GCAAGGTTAA AACTCAAATG AATTGACGGG GGCCCGCACA AGCGGTGGAG CATGTGGTTT AATTCGATGC AACGCGAAGA

Pantoea_dispersa_(KY882077.1) CAAACAGGAT TAGATACCCT GGTAGTCCAC GCCGTAAACG ATGTCGACTT GGAGGTTGTG CCCTTGAGGC GTG-GCTTCC GGAGCTAACG CGTTAAGTCG ACCGCCTGGG GAGTACGGCC GCAAGGTTAA AACTCAAATG AATTGACGGG GGCCCGCACA AGCGGTGGAG CATGTGGTTT AATTCGATGC AACGCGAAGA

PaKu21_-_Bacillus_sonorensis CGAACAGGAT TAGATACCCT GGTAGTCCAC GCCGTAAACG ATGAGTGCTA AGTGTTAGAG GGTTTCCGCC CTTTAGTGCT GCAGCAAACG CATTAAGCAC TCCGCCTGGG GAGTACGGTC GCAAGACTGA AACTCAAAGG AATTGACGGG GGCCCGCACA AGCGGTGGAG CATGTGGTTT AATTCGAAGC AACGCGAAGA

Bacillus_sonorensis_(KU551167. CGAACAGGAT TAGATACCCT GGTAGTCCAC GCCGTAAACG ATGAGTGCTA AGTGTTAGAG GGTTTCCGCC CTTTAGTGCT GCAGCAAACG CATTAAGCAC TCCGCCTGGG GAGTACGGTC GCAAGACTGA AACTCAAAGG AATTGACGGG GGCCCGCACA AGCGGTGGAG CATGTGGTTT AATTCGAAGC AACGCGAAGA

Bacillus_sonorensis_(FN397516. CGAACAGGAT TAGATACCCT GGTAGTCCAC GCCGTAAACG ATGAGTGCTA AGTGTTAGAG GGTTTCCGCC CTTTAGTGCT GCAGCAAACG CATTAAGCAC TCCGCCTGGG GAGTACGGTC GCAAGACTGA AACTCAAAGG AATTGACGGG GGCCCGCACA AGCGGTGGAG CATGTGGTTT AATTCGAAGC AACGCGAAGA

Bacillus_sonorensis_(KU551137. CGAACAGGAT TAGATACCCT GGTAGTCCAC GCCGTAAACG ATGAGTGCTA AGTGTTAGAG GGTTTCCGCC CTTTAGTGCT GCAGCAAACG CATTAAGCAC TCCGCCTGGG GAGTACGGTC GCAAGACTGA AACTCAAAGG AATTGACGGG GGCCCGCACA AGCGGTGGAG CATGTGGTTT AATTCGAAGC AACGCGAAGA

PaKu24_-_Bacillus_sonorensis CGAACAGGAT TAGATACCCT GGTAGTCCAC GCCGTAAACG ATGAGTGCTA AGTGTTAGAG GGTTTCCGCC CTTTAGTGCT GCAGCAAACG CATTAAGCAC TCCGCCTGGG GAGTACGGTC GCAAGACTGA AACTCAAAGG AATTGACGGG GGCCCGCACA AGCGGTGGAG CATGTGGTTT AATTCGAAGC AACGCGAAGA

PaKu22_-_Bacillus_subtilis CGAACAGGAT TAGATACCCT GGTAGTCCAC GCCGTAAACG ATGAGTGCTA AGTGTTAGGG GGTTTCCGCC CCTTAGTGCT GCAGCTAACG CATTAAGCAC TCCGCCTGGG GAGTACGGTC GCAAGACTGA AACTCAAAGG AATTGACGGG GGCCCGCACA AGCGGTGGAG CATGTGGTTT AATTCGAAGC AACGCGAAGA

Bacillus_tequilensis_(MK880583 CGAACAGGAT TAGATACCCT GGTAGTCCAC GCCGTAAACG ATGAGTGCTA AGTGTTAGGG GGTTTCCGCC CCTTAGTGCT GCAGCTAACG CATTAAGCAC TCCGCCTGGG GAGTACGGTC GCAAGACTGA AACTCAAAGG AATTGACGGG GGCCCGCACA AGCGGTGGAG CATGTGGTTT AATTCGAAGC AACGCGAAGA

Bacillus_subtilis_(MK765023.1) CGAACAGGAT TAGATACCCT GGTAGTCCAC GCCGTAAACG ATGAGTGCTA AGTGTTAGGG GGTTTCCGCC CCTTAGTGCT GCAGCTAACG CATTAAGCAC TCCGCCTGGG GAGTACGGTC GCAAGACTGA AACTCAAAGG AATTGACGGG GGCCCGCACA AGCGGTGGAG CATGTGGTTT AATTCGAAGC AACGCGAAGA

PaKu23_-_Bacillus_subtilis CGAACAGGAT TAGATACCCT GGTAGTCCAC GCCGTAAACG ATGAGTGCTA AGTGTTAGGG GGTTTCCGCC CCTTAGTGCT GCAGCTAACG CATTAAGCAC TCCGCCTGGG GAGTACGGTC GCAAGACTGA AACTCAAAGG AATTGACGGG GGCCCGCACA AGCGGTGGAG CATGTGGTTT AATTCGAAGC AACGCGAAGA

Bacillus_subtilis_(MK511833.1) CGAACAGGAT TAGATACCCT GGTAGTCCAC GCCGTAAACG ATGAGTGCTA AGTGTTAGGG GGTTTCCGCC CCTTAGTGCT GCAGCTAACG CATTAAGCAC TCCGCCTGGG GAGTACGGTC GCAAGACTGA AACTCAAAGG AATTGACGGG GGCCCGCACA AGCGGTGGAG CATGTGGTTT AATTCGAAGC AACGCGAAGA

Bacillus_subtilis_(KX450400.1) CGAACAGGAT TAGATACCCT GGTAGTCCAC GCCGTAAACG ATGAGTGCTA AGTGTTAGGG GGTTTCCGCC CCTTAGTGCT GCAGCTAACG CATTAAGCAC TCCGCCTGGG GAGTACGGTC GCAAGACTGA AACTCAAAGG AATTGACGGG GGCCCGCACA AGCGGTGGAG CATGTGGTTT AATTCGAAGC AACGCGAAGA

PaKu25_-_Bacillus_cereus CAAACAGGAT TAGATACCCT GGTAGTCCAC GCCGTAAACG ATGAGTGCTA AGTGTTAGAG GGTTTCCGCC CTTTAGTGCT GAAGTTAACG CATTAAGCAC TCCGCCTGGG GAGTACGGCC GCAAGGCTGA AACTCAAAGG AATTGACGGG GGCCCGCACA AGCGGTGGAG CATGTGGTTT AATTCGAAGC AACGCGAAGA

Bacillus_thuringiensis_(CP0378 CAAACAGGAT TAGATACCCT GGTAGTCCAC GCCGTAAACG ATGAGTGCTA AGTGTTAGAG GGTTTCCGCC CTTTAGTGCT GAAGTTAACG CATTAAGCAC TCCGCCTGGG GAGTACGGCC GCAAGGCTGA AACTCAAAGG AATTGACGGG GGCCCGCACA AGCGGTGGAG CATGTGGTTT AATTCGAAGC AACGCGAAGA

Bacillus_mobilis_(CP031443.1) CAAACAGGAT TAGATACCCT GGTAGTCCAC GCCGTAAACG ATGAGTGCTA AGTGTTAGAG GGTTTCCGCC CTTTAGTGCT GAAGTTAACG CATTAAGCAC TCCGCCTGGG GAGTACGGCC GCAAGGCTGA AACTCAAAGG AATTGACGGG GGCCCGCACA AGCGGTGGAG CATGTGGTTT AATTCGAAGC AACGCGAAGA

Bacillus_thuringiensis_(CP0357 CAAACAGGAT TAGATACCCT GGTAGTCCAC GCCGTAAACG ATGAGTGCTA AGTGTTAGAG GGTTTCCGCC CTTTAGTGCT GAAGTTAACG CATTAAGCAC TCCGCCTGGG GAGTACGGCC GCAAGGCTGA AACTCAAAGG AATTGACGGG GGCCCGCACA AGCGGTGGAG CATGTGGTTT AATTCGAAGC AACGCGAAGA

Bacillus_cereus_(CP030982.1) CAAACAGGAT TAGATACCCT GGTAGTCCAC GCCGTAAACG ATGAGTGCTA AGTGTTAGAG GGTTTCCGCC CTTTAGTGCT GAAGTTAACG CATTAAGCAC TCCGCCTGGG GAGTACGGCC GCAAGGCTGA AACTCAAAGG AATTGACGGG GGCCCGCACA AGCGGTGGAG CATGTGGTTT AATTCGAAGC AACGCGAAGA

Bacillus_cereus_(MH068823.1) CAAACAGGAT TAGATACCCT GGTAGTCCAC GCCGTAAACG ATGAGTGCTA AGTGTTAGAG GGTTTCCGCC CTTTAGTGCT GAAGTTAACG CATTAAGCAC TCCGCCTGGG GAGTACGGCC GCAAGGCTGA AACTCAAAGG AATTGACGGG GGCCCGCACA AGCGGTGGAG CATGTGGTTT AATTCGAAGC AACGCGAAGA

Bacillus_thuringiensis_(MG7453 CAAACAGGAT TAGATACCCT GGTAGTCCAC GCCGTAAACG ATGAGTGCTA AGTGTTAGAG GGTTTCCGCC CTTTAGTGCT GAAGTTAACG CATTAAGCAC TCCGCCTGGG GAGTACGGCC GCAAGGCTGA AACTCAAAGG AATTGACGGG GGCCCGCACA AGCGGTGGAG CATGTGGTTT AATTCGAAGC AACGCGAAGA

Rhizobium_massiliae(Country-_T CAAACAGGAT TAGATACCCT GGTAGTCCAC GCCGTAAACG ATGAATGTTA GCCGTCGGGC AGTAT--ACT GTTCGGTGGC GCAGCTAACG CATTAAACAT TCTGCTTGGG GAGTACGGTC GCAAGATTAA AACTCAAAGG AATTGACGGG GGCCCGCACA AGCGGTGGAG CATGTGGTTT AATTCGAAGC AACGCGCAGA

Stenotrophomonas_maltophilia_( CAAACAGGAT TAGATACCCT GGTAGTCCAC GCCCTAAACG ATGCGAACTG GATGTTGGGT GCAATTTGGC ACGCAGTATC GAAGCTAACG CGTTAAGTTC GCCGCCTGGG GAGTACGGTC GCAAGACTGA AACTCAAAGG AATTGACGGG GGCCCGCACA AGCGGTGGAG TATGTGGTTT AATTCGATGC AACGCGAAGA

Serratia_sp._(Country-_Tunisia CAAATAGGAT TAGATACCTT GGTAGTCCAC GCTGTAAATG ATGTCGACCC GGAGGTTGTG CCCTTGAGGC GCG-GCTTCC GGAGCCAACG CGCTAAATCG ACCGCTTGGG GAGTACGGCC GCAAGGTTAA AACTCAAATG AATTGACGGG GGCCCGCACA AGCGGTGGAG CATGTGGTCC ACTCCGACGC AACGCGAAGA

Staphylococcus_saprophyticus_( CAAACAGGAT TAGATACCCT GGTAGTCCAC GCCGTAAACG ATGAGTGCTA AGTGTTAGGG GGTTTCCGCC CCTTAGTGCT GCAGCTAACG CATTAAGCAC TCCGCCTGGG GAGTACGACC GCAAGGTTGA AACTCAAAGG AATTGACGGG GACCCGCACA AGCGGTGGAG CATGTGGTTT AATTCGAAGC AACGCGAAGA

Staphylococcus_saprophyticus_( CAAACAGGAT TAGATACCCT GGTAGTCCAC GCCGTAAACG ATGAGTGCTA AGTGTTAGGG GGTTTCCGCC CCTTAGTGCT GCAGCTAACG CATTAAGCAC TCCGCCTGGG GAGTACGACC GCAAGGTTGA AACTCAAAGG AATTGACGGG GACCCGCACA AGCGGTGGAG CATGTGGTTT AATTCGAAGC AACGCGAAGA

Bacillus_subtilis_(Country-Ira CGAACAGGAT TAGATACCCT GGTAGTCCAC GCCGTAAACG ATGAGTGCTA AGTGTTAGGG GGTTTCCGCC CCTTAGTGCT GCAGCTAACG CATTAAGCAC TCCGCCTGGG GAGTACGGTC GCAAGACTGA AACTCAAAGG AATTGACGGG GGCCCGCACA AGCGGTGGAG CATGTGGTTT AATTCGAAGC AACGCGAAGA

Bacillus_cereus_(Country-UK)_( CAAACAGGAT TAGATACCCT GGTAGTCCAC GCCGTAAACG ATGAGTGCTA AGTGTTAGAG GGTTTCCGCC CTTTAGTGCT GAAGTTAACG CATTAAGCAC TCCGCCTGGG GAGTACGGCC GCAAGGCTGA AACTCAAAGG AATTGACGGG GGCCCGCACA AGCGGTGGAG CATGTGGTTT AATTCGAAGC AACGCGAAGA

....|....| ....|....| ....|....| ....|....| ....|....| ....|....| ....|....| ....|....| ....|....| ....|....| ....|....| ....|....| ....|....| ....|....| ....|....| ....|....| ....|....| ....|....| ....|....| ....|....|

1005 1015 1025 1035 1045 1055 1065 1075 1085 1095 1105 1115 1125 1135 1145 1155 1165 1175 1185 1195

PaKu1_-_Serratia_marcescens ACCTTACCTA CTCTTGACAT CCAG--AGAA CTTTCCAGAG ATGGATTGGT GCC-TTCGGG AA-CTCTGAG ACAGGTGCTG CATGGCTGTC GTCAGCTCGT GTTGTGAAAT GTTGGGTTAA GTCCCGCAAC GAGCGCAACC CTTATCCTTT GTTGCCAGCG -GTTCGGCCG GGAACTCAAA GGAGACTGCC AGTGATAAAC

Serratia_marcescens_(KX911721. ACCTTACCTA CTCTTGACAT CCAG--AGAA CTTTCCAGAG ATGGATTGGT GCC-TTCGGG AA-CTCTGAG ACAGGTGCTG CATGGCTGTC GTCAGCTCGT GTTGTGAAAT GTTGGGTTAA GTCCCGCAAC GAGCGCAACC CTTATCCTTT GTTGCCAGCG -GTTCGGCCG GGAACTCAAA GGAGACTGCC AGTGATAAAC

Serratia_marcescens_(KT992361. ACCTTACCTA CTCTTGACAT CCAG--AGAA CTTTCCAGAG ATGGATTGGT GCC-TTCGGG AA-CTCTGAG ACAGGTGCTG CATGGCTGTC GTCAGCTCGT GTTGTGAAAT GTTGGGTTAA GTCCCGCAAC GAGCGCAACC CTTATCCTTT GTTGCCAGCG -GTTCGGCCG GGAACTCAAA GGAGACTGCC AGTGATAAAC

PaKu2_-_Enterobacter_sp. ACCTTACCTA CTCTTGACAT CCAG--AGAA CTTACCAGAG ATGCTTTGGT GCC-TTCGGG AA-CTCTGAG ACAGGTGCTG CATGGCTGTC GTCAGCTCGT GTTGTGAAAT GTTGGGTTAA GTCCCGCAAC GAGCGCAACC CTTATCCTTT GTTGCCAGCG -GTTAGGCCG GGAACTCAAA GGAGACTGCC AGTGATAAAC

Enterobacter_sp._(MG280962.1) ACCTTACCTA CTCTTGACAT CCAG--AGAA CTTACCAGAG ATGCTTTGGT GCC-TTCGGG AA-CTCTGAG ACAGGTGCTG CATGGCTGTC GTCAGCTCGT GTTGTGAAAT GTTGGGTTAA GTCCCGCAAC GAGCGCAACC CTTATCCTTT GTTGCCAGCG -GTTAGGCCG GGAACTCAAA GGAGACTGCC AGTGATAAAC

Enterobacter_hormaechei_(CP029 ACCTTACCTA CTCTTGACAT CCAG--AGAA CTTAGCAGAG ATGCTTTGGT GCC-TTCGGG AA-CTCTGAG ACAGGTGCTG CATGGCTGTC GTCAGCTCGT GTTGTGAAAT GTTGGGTTAA GTCCCGCAAC GAGCGCAACC CTTATCCTTT GTTGCCAGCG -GTTAGGCCG GGAACTCAAA GGAGACTGCC AGTGATAAAC

PaKu3_-_Staphylococcus_saproph ACCTTACCAA ATCTTGACAT CCTT--TGAA AACTCTAGAG ATAGAGCTTT CCCCTTCGGG GGACAAAGTG ACAGGTGGTG CATGGTTGTC GTCAGCTCGT GTCGTGAGAT GTTGGGTTAA GTCCCGCAAC GAGCGCAACC CTTAAGCTTA GTTGCCATCA --TTAAGTTG GGCACTCTAG GTTGACTGCC GGTGACAAAC

Staphylococcus_saprophyticus_( ACCTTACCAA ATCTTGACAT CCTT--TGAA AACTCTAGAG ATAGAGCTTT CCCCTTCGGG GGACAAAGTG ACAGGTGGTG CATGGTTGTC GTCAGCTCGT GTCGTGAGAT GTTGGGTTAA GTCCCGCAAC GAGCGCAACC CTTAAGCTTA GTTGCCATCA --TTAAGTTG GGCACTCTAG GTTGACTGCC GGTGACAAAC

Staphylococcus_saprophyticus_( ACCTTACCAA ATCTTGACAT CCTT--TGAA AACTCTAGAG ATAGAGCTTT CCCCTTCGGG GGACAAAGTG ACAGGTGGTG CATGGTTGTC GTCAGCTCGT GTCGTGAGAT GTTGGGTTAA GTCCCGCAAC GAGCGCAACC CTTAAGCTTA GTTGCCATCA --TTAAGTTG GGCACTCTAG GTTGACTGCC GGTGACAAAC

PaKu4_-_Enterobacter_cloacae ACCTTACCTG GTCTTGACAT CCAC--AGAA CTTTCCAGAG ATGGATTGGT GCC-TTCGGG AA-CTGTGAG ACAGGTGCTG CATGGCTGTC GTCAGCTCGT GTTGTGAAAT GTTGGGTTAA GTCCCGCAAC GAGCGCAACC CTTATCCTTT GTTGCCAGCG -GTTAGGCCG GGAACTCAAA GGAGACTGCC AGTGATAAAC

Uncultured_Enterobacter_sp._(M ACCTTACCTG GTCTTGACAT CCAC--AGAA CTTTCCAGAG ATGGATTGGT GCC-TTCGGG AA-CTGTGAG ACAGGTGCTG CATGGCTGTC GTCAGCTCGT GTTGTGAAAT GTTGGGTTAA GTCCCGCAAC GAGCGCAACC CTTATCCTTT GTTGCCAGCG -GTTAGGCCG GGAACTCAAA GGAGACTGCC AGTGATAAAC

Enterobacter_cloacae_(KY930709 ACCTTACCTG GTCTTGACAT CCAC--AGAA CTTTCCAGAG ATGGATTGGT GCC-TTCGGG AA-CTGTGAG ACAGGTGCTG CATGGCTGTC GTCAGCTCGT GTTGTGAAAT GTTGGGTTAA GTCCCGCAAC GAGCGCAACC CTTATCCTTT GTTGCCAGCG -GTTAGGCCG GGAACTCAAA GGAGACTGCC AGTGATAAAC

PaKu5_-_Enterobacter_cloacae ACCTTACCTG GTCTTGACAT CCAC--AGAA CTTTCCAGAG ATGGATTGGT GCC-TTCGGG AA-CTGTGAG ACAGGTGCTG CATGGCTGTC GTCAGCTCGT GTTGTGAAAT GTTGGGTTAA GTCCCGCAAC GAGCGCAACC CTTATCCTTT GTTGCCAGCG -GTCCGGCCG GGAACTCAAA GGAGACTGCC AGTGATAAAC

Enterobacter_sp._(KU867838.1) ACCTTACCTG GTCTTGACAT CCAC--AGAA CTTTCCAGAG ATGGATTGGT GCC-TTCGGG AA-CTGTGAG ACAGGTGCTG CATGGCTGTC GTCAGCTCGT GTTGTGAAAT GTTGGGTTAA GTCCCGCAAC GAGCGCAACC CTTATCCTTT GTTGCCAGCG -GTCCGGCCG GGAACTCAAA GGAGACTGCC AGTGATAAAC

Enterobacter_cloacae_(KX242265 ACCTTACCTG GTCTTGACAT CCAC--AGAA CTTTCCAGAG ATGGATTGGT GCC-TTCGGG AA-CTGTGAG ACAGGTGCTG CATGGCTGTC GTCAGCTCGT GTTGTGAAAT GTTGGGTTAA GTCCCGCAAC GAGCGCAACC CTTATCCTTT GTTGCCAGCG -GTCCGGCCG GGAACTCAAA GGAGACTGCC AGTGATAAAC

Enterobacter_cloacae_(KP794925 ACCTTACCTG GTCTTGACAT CCAC--AGAA CTTTCCAGAG ATGGATTGGT GCC-TTCGGG AA-CTGTGAG ACAGGTGCTG CATGGCTGTC GTCAGCTCGT GTTGTGAAAT GTTGGGTTAA GTCCCGCAAC GAGCGCAACC CTTATCCTTT GTTGCCAGCG -GTCCGGCCG GGAACTCAAA GGAGACTGCC AGTGATAAAC

PaKu6_-_Staphylococcus_sciuri ACCTTACCAA ATCTTGACAT CCTT--TGAC CGCTCTAGAG ATAGAGTCTT CCCCTTCGGG GGACAAAGTG ACAGGTGGTG CATGGTTGTC GTCAGCTCGT GTCGTGAGAT GTTGGGTTAA GTCCCGCAAC GAGCGCAACC CTTAAGCTTA GTTGCCATCA --TTAAGTTG GGCACTCTAG GTTGACTGCC GGTGACAAAC

Staphylococcus_sciuri_(MG70600 ACCTTACCAA ATCTTGACAT CCTT--TGAC CGCTCTAGAG ATAGAGTCTT CCCCTTCGGG GGACAAAGTG ACAGGTGGTG CATGGTTGTC GTCAGCTCGT GTCGTGAGAT GTTGGGTTAA GTCCCGCAAC GAGCGCAACC CTTAAGCTTA GTTGCCATCA --TTAAGTTG GGCACTCTAG GTTGACTGCC GGTGACAAAC

Staphylococcus_lentus_(MG98829 ACCTTACCAA ATCTTGACAT CCTT--TGAC CGCTCTAGAG ATAGAGTCTT CCCCTTCGGG GGACAAAGTG ACAGGTGGTG CATGGTTGTC GTCAGCTCGT GTCGTGAGAT GTTGGGTTAA GTCCCGCAAC GAGCGCAACC CTTAAGCTTA GTTGCCATCA --TTAAGTTG GGCACTCTAG GTTGACTGCC GGTGACAAAC

Staphylococcus_sciuri_(MG51742 ACCTTACCAA ATCTTGACAT CCTT--TGAC CGCTCTAGAG ATAGAGTCTT CCCCTTCGGG GGACAAAGTG ACAGGTGGTG CATGGTTGTC GTCAGCTCGT GTCGTGAGAT GTTGGGTTAA GTCCCGCAAC GAGCGCAACC CTTAAGCTTA GTTGCCATCA --TTAAGTTG GGCACTCTAG GTTGACTGCC GGTGACAAAC

PaKu8_-_Pseudomonas_aeruginosa ACCTTACCTG GCCTTGACAT GCTG--AGAA CTTTCCAGAG ATGGATTGGT GCC-TTCGGG AA-CTCAGAC ACAGGTGCTG CATGGCTGTC GTCAGCTCGT GTCGTGAGAT GTTGGGTTAA GTCCCGTAAC GAGCGCAACC CTTGTCCTTA GTTACCAGCA CCTCG-GGTG GGCACTCTAA GGAGACTGCC GGTGACAAAC

Uncultured_bacterium_(FM996243 ACCTTACCTG GCCTTGACAT GCTG--AGAA CTTTCCAGAG ATGGATTGGT GCC-TTCGGG AA-CTCAGAC ACAGGTGCTG CATGGCTGTC GTCAGCTCGT GTCGTGAGAT GTTGGGTTAA GTCCCGTAAC GAGCGCAACC CTTGTCCTTA GTTACCAGCA CCTCG-GGTG GGCACTCTAA GGAGACTGCC GGTGACAAAC

Pseudomonas_aeruginosa_(MK4304 ACCTTACCTG GCCTTGACAT GCTG--AGAA CTTTCCAGAG ATGGATTGGT GCC-TTCGGG AA-CTCAGAC ACAGGTGCTG CATGGCTGTC GTCAGCTCGT GTCGTGAGAT GTTGGGTTAA GTCCCGTAAC GAGCGCAACC CTTGTCCTTA GTTACCAGCA CCTCG-GGTG GGCACTCTAA GGAGACTGCC GGTGACAAAC

PaKu9_-_Staphylococcus_arletta ACCTTACCAA ATCTTGACAT CCTT--TGAC CACTCTAGAG ATAGAGCTTT CCCCTTCGGG GGACAAAGTG ACAGGTGGTG CATGGTTGTC GTCAGCTCGT GTCGTGAGAT GTTGGGTTAA GTCCCGCAAC GAGCGCAACC CTTAAACTTA GTTGCCAGCA --TTTAGTTG GGCACTCTAG GTTGACTGCC GGTGACAAAC

Staphylococcus_arlettae_(KX344 ACCTTACCAA ATCTTGACAT CCTT--TGAC CACTCTAGAG ATAGAGCTTT CCCCTTCGGG GGACAAAGTG ACAGGTGGTG CATGGTTGTC GTCAGCTCGT GTCGTGAGAT GTTGGGTTAA GTCCCGCAAC GAGCGCAACC CTTAAACTTA GTTGCCAGCA --TTTAGTTG GGCACTCTAG GTTGACTGCC GGTGACAAAC

Staphylococcus_sp._(KU891836.1 ACCTTACCAA ATCTTGACAT CCTT--TGAC CACTCTAGAG ATAGAGCTTT CCCCTTCGGG GGACAAAGTG ACAGGTGGTG CATGGTTGTC GTCAGCTCGT GTCGTGAGAT GTTGGGTTAA GTCCCGCAAC GAGCGCAACC CTTAAACTTA GTTGCCAGCA --TTTAGTTG GGCACTCTAG GTTGACTGCC GGTGACAAAC

PaKu10_-_Aeromonas_caviae ACCTTACCTG GCCTTGACAT GTCT--GGAA TCCTGCAGAG ATGCGGGAGT GCC-TTCGGG AA-TCAGAAC ACAGGTGCTG CATGGCTGTC GTCAGCTCGT GTCGTGAGAT GTTGGGTTAA GTCCCGCAAC GAGCGCAACC CCTGTCCTTT GTTGCCAGCA CGTAATGGTG GGAACTCAAG GGAGACTGCC GGTGATAAAC

Uncultured_bacterium_(KX242397 ACCTTACCTG GCCTTGACAT GTCT--GGAA TCCTGCAGAG ATGCGGGAGT GCC-TTCGGG AA-TCAGAAC ACAGGTGCTG CATGGCTGTC GTCAGCTCGT GTCGTGAGAT GTTGGGTTAA GTCCCGCAAC GAGCGCAACC CCTGTCCTTT GTTGCCAGCA CGTAATGGTG GGAACTCAAG GGAGACTGCC GGTGATAAAC

Aeromonas_caviae_(HQ407268.1) ACCTTACCTG GCCTTGACAT GTCT--GGAA TCCTGCAGAG ATGCGGGAGT GCC-TTCGGG AA-TCAGAAC ACAGGTGCTG CATGGCTGTC GTCAGCTCGT GTCGTGAGAT GTTGGGTTAA GTCCCGCAAC GAGCGCAACC CCTGTCCTTT GTTGCCAGCA CGTAATGGTG GGAACTCAAG GGAGACTGCC GGTGATAAAC

Aeromonas_aquariorum_(EU085557 ACCTTACCTG GCCTTGACAT GTCT--GGAA TCCTGCAGAG ATGCGGGAGT GCC-TTCGGG AA-TCAGAAC ACAGGTGCTG CATGGCTGTC GTCAGCTCGT GTCGTGAGAT GTTGGGTTAA GTCCCGCAAC GAGCGCAACC CCTGTCCTTT GTTGCCAGCA CGTAATGGTG GGAACTCAAG GGAGACTGCC GGTGATAAAC

PaKu11_-_Aeromonas_caviae ACCTTACCTG GCCTTGACAT GTCT--GGAA TCCTGTAGAG ATACGGGAGT GCC-TTCGGG AA-TCAGAAC ACAGGTGCTG CATGGCTGTC GTCAGCTCGT GTCGTGAGAT GTTGGGTTAA GTCCCGCAAC GAGCGCAACC CCTGTCCTTT GTTGCCAGCA CGTAATGGTG GGAACTCAAG GGAGACTGCC GGTGATAAAC

Aeromonas_caviae_(KY087979.1) ACCTTACCTG GCCTTGACAT GTCT--GGAA TCCTGTAGAG ATACGGGAGT GCC-TTCGGG AA-TCAGAAC ACAGGTGCTG CATGGCTGTC GTCAGCTCGT GTCGTGAGAT GTTGGGTTAA GTCCCGCAAC GAGCGCAACC CCTGTCCTTT GTTGCCAGCA CGTAATGGTG GGAACTCAAG GGAGACTGCC GGTGATAAAC

Uncultured_Aeromonas_sp._(KU94 ACCTTACCTG GCCTTGACAT GTCT--GGAA TCCTGTAGAG ATACGGGAGT GCC-TTCGGG AA-TCAGAAC ACAGGTGCTG CATGGCTGTC GTCAGCTCGT GTCGTGAGAT GTTGGGTTAA GTCCCGCAAC GAGCGCAACC CCTGTCCTTT GTTGCCAGCA CGTAATGGTG GGAACTCAAG GGAGACTGCC GGTGATAAAC

PaKu12_-_Staphylococcus_warner ACCTTACCAA ATCTTGACAT CCTT--TGAC CACTCTAGAG ATAGAGTTTT CCCCTTCGGG GGACAAAGTG ACAGGTGGTG CATGGTTGTC GTCAGCTCGT GTCGTGAGAT GTTGGGTTAA GTCCCGCAAC GAGCGCAACC CTTAAGCTTA GTTGCCATCA --TTAAGTTG GGCACTCTAA GTTGACTGCC GGTGACAAAC

Staphylococcus_warneri_(MH0149 ACCTTACCAA ATCTTGACAT CCTT--TGAC CGCTCTAGAG ATAGAGTTTT CCCCTTCGGG GGACAAAGTG ACAGGTGGTG CATGGTTGTC GTCAGCTCGT GTCGTGAGAT GTTGGGTTAA GTCCCGCAAC GAGCGCAACC CTTAAGCTTA GTTGCCATCA --TTAAGTTG GGCACTCTAA GTTGACTGCC GGTGACAAAC

Staphylococcus_warneri_(KF3063 ACCTTACCAA ATCTTGACAT CCTT--TGAC CGCTCTAGAG ATAGAGTTTT CCCCTTCGGG GGACAAAGTG ACAGGTGGTG CATGGTTGTC GTCAGCTCGT GTCGTGAGAT GTTGGGTTAA GTCCCGCAAC GAGCGCAACC CTTAAGCTTA GTTGCCATCA --TTAAGTTG GGCACTCTAA GTTGACTGCC GGTGACAAAC

PaKu13_-_Bacillus_megaterium ACCTTACCAG GTCTTGACAT CCTC--TGAC AACTCTAGAG ATAGAGCGTT CCCCTTCGGG GGACAGAGTG ACAGGTGGTG CATGGTTGTC GTCAGCTCGT GTCGTGAGAT GTTGGGTTAA GTCCCGCAAC GAGCGCAACC CTTGATCTTA GTTGCCAGCA --TTTAGTTG GGCACTCTAA GGTGACTGCC GGTGACAAAC

Bacillus_sp._(MK691444.1) ACCTTACCAG GTCTTGACAT CCTC--TGAC AACTCTAGAG ATAGAGCGTT CCCCTTCGGG GGACAGAGTG ACAGGTGGTG CATGGTTGTC GTCAGCTCGT GTCGTGAGAT GTTGGGTTAA GTCCCGCAAC GAGCGCAACC CTTGATCTTA GTTGCCAGCA --TTTAGTTG GGCACTCTAA GGTGACTGCC GGTGACAAAC

Bacillus_sp._(LC373523.1) ACCTTACCAG GTCTTGACAT CCTC--TGAC AACTCTAGAG ATAGAGCGTT CCCCTTCGGG GGACAGAGTG ACAGGTGGTG CATGGTTGTC GTCAGCTCGT GTCGTGAGAT GTTGGGTTAA GTCCCGCAAC GAGCGCAACC CTTGATCTTA GTTGCCAGCA --TTTAGTTG GGCACTCTAA GGTGACTGCC GGTGACAAAC

Bacillus_aryabhattai_(MK474942 ACCTTACCAG GTCTTGACAT CCTC--TGAC AACTCTAGAG ATAGAGCGTT CCCCTTCGGG GGACAGAGTG ACAGGTGGTG CATGGTTGTC GTCAGCTCGT GTCGTGAGAT GTTGGGTTAA GTCCCGCAAC GAGCGCAACC CTTGATCTTA GTTGCCAGCA --TTTAGTTG GGCACTCTAA GGTGACTGCC GGTGACAAAC

Bacillus_aryabhattai_(MK474941 ACCTTACCAG GTCTTGACAT CCTC--TGAC AACTCTAGAG ATAGAGCGTT CCCCTTCGGG GGACAGAGTG ACAGGTGGTG CATGGTTGTC GTCAGCTCGT GTCGTGAGAT GTTGGGTTAA GTCCCGCAAC GAGCGCAACC CTTGATCTTA GTTGCCAGCA --TTTAGTTG GGCACTCTAA GGTGACTGCC GGTGACAAAC

Bacillus_megaterium_(KX298860. ACCTTACCAG GTCTTGACAT CCTC--TGAC AACTCTAGAG ATAGAGCGTT CCCCTTCGGG GGACAGAGTG ACAGGTGGTG CATGGTTGTC GTCAGCTCGT GTCGTGAGAT GTTGGGTTAA GTCCCGCAAC GAGCGCAACC CTTGATCTTA GTTGCCAGCA --TTTAGTTG GGCACTCTAA GGTGACTGCC GGTGACAAAC

PaKu14_-_Pseudomonas_stutzeri ACCTTACCAG GCCTTGACAT GCAG--AGAA CTTTCCAGAG ATGGATTGGT GCC-TTCGGG AA-CTCTGAC ACAGGTGCTG CATGGCTGTC GTCAGCTCGT GTCGTGAGAT GTTGGGTTAA GTCCCGTAAC GAGCGCAACC CTTGTCCTTA GTTACCAGCA CGTTAAGGTG GGCACTCTAA GGAGACTGCC GGTGACAAAC

Pseudomonas_stutzeri_(MG892782 ACCTTACCAG GCCTTGACAT GCAG--AGAA CTTTCCAGAG ATGGATTGGT GCC-TTCGGG AA-CTCTGAC ACAGGTGCTG CATGGCTGTC GTCAGCTCGT GTCGTGAGAT GTTGGGTTAA GTCCCGTAAC GAGCGCAACC CTTGTCCTTA GTTACCAGCA CGTTAAGGTG GGCACTCTAA GGAGACTGCC GGTGACAAAC

Pseudomonas_stutzeri_(MG595371 ACCTTACCAG GCCTTGACAT GCAG--AGAA CTTTCCAGAG ATGGATTGGT GCC-TTCGGG AA-CTCTGAC ACAGGTGCTG CATGGCTGTC GTCAGCTCGT GTCGTGAGAT GTTGGGTTAA GTCCCGTAAC GAGCGCAACC CTTGTCCTTA GTTACCAGCA CGTTAAGGTG GGCACTCTAA GGAGACTGCC GGTGACAAAC

PaKu15_-_Rhizobium_sp. ACCTTACCAG CTCTTGACAT TCGGGGTATG GGCATTGGAG ACGATGTCCT TCAGTTAGGC TGGCCCCAGA ACAGGTGCTG CATGGCTGTC GTCAGCTCGT GTCGTGAGAT GTTGGGTTAA GTCCCGCAAC GAGCGCAACC CTCGCCCTTA GTTGCCAGCA --TTTAGTTG GGCACTCTAA GGGGACTGCC GGTGATAAGC

Rhizobium_pusense_(CP039895.1) ACCTTACCAG CTCTTGACAT TCGGGGTATG GGCATTGGAG ACGATGTCCT TCAGTTAGGC TGGCCCCAGA ACAGGTGCTG CATGGCTGTC GTCAGCTCGT GTCGTGAGAT GTTGGGTTAA GTCCCGCAAC GAGCGCAACC CTCGCCCTTA GTTGCCAGCA --TTTAGTTG GGCACTCTAA GGGGACTGCC GGTGATAAGC

Rhizobium_sp._(KY971009.1) ACCTTACCAG CTCTTGACAT TCGGGGTATG GGCATTGGAG ACGATGTCCT TCAGTTAGGC TGGCCCCAGA ACAGGTGCTG CATGGCTGTC GTCAGCTCGT GTCGTGAGAT GTTGGGTTAA GTCCCGCAAC GAGCGCAACC CTCGCCCTTA GTTGCCAGCA --TTTAGTTG GGCACTCTAA GGGGACTGCC GGTGATAAGC

PaKu7_-_Stenotrophomonas_malto ACCTTACCTG GCCTTGACAT GTCG--AGAA CTTTCCAGAG ATGGATTGGT GCC-TTCGGG AA-CTCGAAC ACAGGTGCTG CATGGCTGTC GTCAGCTCGT GTCGTGAGAT GTTGGGTTAA GTCCCGCAAC GAGCGCAACC CTTGTCCTTA GTTGCCAGCA CGTAATGGTG GGAACTCTAA GGAGACCGCC GGTGACAAAC

Stenotrophomonas_maltophilia_( ACCTTACCTG GCCTTGACAT GTCG--AGAA CTTTCCAGAG ATGGATTGGT GCC-TTCGGG AA-CTCGAAC ACAGGTGCTG CATGGCTGTC GTCAGCTCGT GTCGTGAGAT GTTGGGTTAA GTCCCGCAAC GAGCGCAACC CTTGTCCTTA GTTGCCAGCA CGTAATGGTG GGAACTCTAA GGAGACCGCC GGTGACAAAC

Stenotrophomonas_maltophilia_( ACCTTACCTG GCCTTGACAT GTCG--AGAA CTTTCCAGAG ATGGATTGGT GCC-TTCGGG AA-CTCGAAC ACAGGTGCTG CATGGCTGTC GTCAGCTCGT GTCGTGAGAT GTTGGGTTAA GTCCCGCAAC GAGCGCAACC CTTGTCCTTA GTTGCCAGCA CGTAATGGTG GGAACTCTAA GGAGACCGCC GGTGACAAAC

PaKu16_-_Stenotrophomonas_malt ACCTTACCTG GCCTTGACAT GTCG--AGAA CTTTCCAGAG ATGGATGGGT GCC-TTCGGG AA-CTCGAAC ACAGGTGCTG CATGGCTGTC GTCAGCTCGT GTCGTGAGAT GTTGGGTTAA GTCCCGCAAC GAGCGCAACC CTTGTCCTTA GTTGCCAGCA CGTAATGGTG GGAACTCTAA GGAGACCGCC GGTGACAAAC

Stenotrophomonas_maltophilia_( ACCTTACCTG GCCTTGACAT GTCG--AGAA CTTTCCAGAG ATGGATGGGT GCC-TTCGGG AA-CTCGAAC ACAGGTGCTG CATGGCTGTC GTCAGCTCGT GTCGTGAGAT GTTGGGTTAA GTCCCGCAAC GAGCGCAACC CTTGTCCTTA GTTGCCAGCA CGTAATGGTG GGAACTCTAA GGAGACCGCC GGTGACAAAC

Stenotrophomonas_maltophilia_( ACCTTACCTG GCCTTGACAT GTCG--AGAA CTTTCCAGAG ATGGATGGGT GCC-TTCGGG AA-CTCGAAC ACAGGTGCTG CATGGCTGTC GTCAGCTCGT GTCGTGAGAT GTTGGGTTAA GTCCCGCAAC GAGCGCAACC CTTGTCCTTA GTTGCCAGCA CGTAATGGTG GGAACTCTAA GGAGACCGCC GGTGACAAAC

PaKu17_-_Stenotrophomonas_malt ACCTTACCTG GCCTTGACAT GTCG--AGAA CTTTCCAGAG ATGGATGGGT GCC-TTCGGG AA-CTCGAAC ACAGGTGCTG CATGGCTGTC GTCAGCTCGT GTCGTGAGAT GTTGGGTTAA GTCCCGCAAC GAGCGCAACC CTTGTCCTTA GTTGCCAGCA CGTAATGGTG GGAACTCTAA GGAGACCGCC GGTGACAAAC

PaKu26_-_Stenotrophomonas_malt ACCTTACCTG GCCTTGACAT GTCG--AGAA CTTTCCAGAG ATGGATGGGT GCC-TTCGGG AA-CTCGAAC ACAGGTGCTG CATGGCTGTC GTCAGCTCGT GTCGTGAGAT GTTGGGTTAA GTCCCGCAAC GAGCGCAACC CTTGTCCTTA GTTGCCAGCA CGTAATGGTG GGAACTCTAA GGAGACCGCC GGTGACAAAC

Stenotrophomonas_maltophilia_( ACCTTACCTG GCCTTGACAT GTCG--AGAA CTTTCCAGAG ATGGATGGGT GCC-TTCGGG AA-CTCGAAC ACAGGTGCTG CATGGCTGTC GTCAGCTCGT GTCGTGAGAT GTTGGGTTAA GTCCCGCAAC GAGCGCAACC CTTGTCCTTA GTTGCCAGCA CGTAATGGTG GGAACTCTAA GGAGACCGCC GGTGACAAAC

PaKu18_-_Stenotrophomonas_pana ACCTTACCTG GTCTTGACAT GTCG--AGAA CTTTCCAGAG ATGGATTGGT GCC-TTCGGG AA-CTCGAAC ACAGGTGCTG CATGGCTGTC GTCAGCTCGT GTCGTGAGAT GTTGGGTTAA GTCCCGCAAC GAGCGCAACC CTTGTCCTTA GTTGCCAGCA CGTAATGGTG GGAACTCTAA GGAGACCGCC GGTGACAAAC

Stenotrophomonas_panacihumi_(M ACCTTACCTG GTCTTGACAT GTCG--AGAA CTTTCCAGAG ATGGATTGGT GCC-TTCGGG AA-CTCGAAC ACAGGTGCTG CATGGCTGTC GTCAGCTCGT GTCGTGAGAT GTTGGGTTAA GTCCCGCAAC GAGCGCAACC CTTGTCCTTA GTTGCCAGCA CGTAATGGTG GGAACTCTAA GGAGACCGCC GGTGACAAAC

Stenotrophomonas_panacihumi_(K ACCTTACCTG GTCTTGACAT GTCG--AGAA CTTTCCAGAG ATGGATTGGT GCC-TTCGGG AA-CTCGAAC ACAGGTGCTG CATGGCTGTC GTCAGCTCGT GTCGTGAGAT GTTGGGTTAA GTCCCGCAAC GAGCGCAACC CTTGTCCTTA GTTGCCAGCA CGTAATGGTG GGAACTCTAA GGAGACCGCC GGTGACAAAC

PaKu19_-_Bacillus_licheniformi ACCTTACCAG GTCTTGACAT CCTC--TGAC AACCCTAGAG ATAGGGC-TT CCCCTTCGGG GG-CAGAGTG ACAGGTGGTG CATGGTTGTC GTCAGCTCGT GTCGTGAGAT GTTGGGTTAA GTCCCGCAAC GAGCGCAACC CTTGATCTTA GTTGCCAGCA --TTCAGTTG GGCACTCTAA GGTGACTGCC GGTGACAAAC

Bacillus_licheniformis_(KJ5722 ACCTTACCAG GTCTTGACAT CCTC--TGAC AACCCTAGAG ATAGGGC-TT CCCCTTCGGG GG-CAGAGTG ACAGGTGGTG CATGGTTGTC GTCAGCTCGT GTCGTGAGAT GTTGGGTTAA GTCCCGCAAC GAGCGCAACC CTTGATCTTA GTTGCCAGCA --TTCAGTTG GGCACTCTAA GGTGACTGCC GGTGACAAAC

Bacillus_licheniformis_(KT1536 ACCTTACCAG GTCTTGACAT CCTC--TGAC AACCCTAGAG ATAGGGC-TT CCCCTTCGGG GG-CAGAGTG ACAGGTGGTG CATGGTTGTC GTCAGCTCGT GTCGTGAGAT GTTGGGTTAA GTCCCGCAAC GAGCGCAACC CTTGATCTTA GTTGCCAGCA --TTCAGTTG GGCACTCTAA GGTGACTGCC GGTGACAAAC

PaKu20_-__Pantoea_dispersa ACCTTACCTG GCCTTGACAT CCAG--AGAA CTTAGCAGAG ATGCTTTGGT GCC-TTCGGG AA-CTCTGAG ACAGGTGCTG CATGGCTGTC GTCAGCTCGT GTTGTGAAAT GTTGGGTTAA GTCCCGCAAC GAGCGCAACC CTTATCCTTT GTTGCCAGCG -GCTCGGCCG GGAACTCAAA GGAGACTGCC GGTGATAAAC

Pantoea_dispersa_(KY292463.1) ACCTTACCTG GCCTTGACAT CCAG--AGAA CTTAGCAGAG ATGCTTTGGT GCC-TTCGGG AA-CTCTGAG ACAGGTGCTG CATGGCTGTC GTCAGCTCGT GTTGTGAAAT GTTGGGTTAA GTCCCGCAAC GAGCGCAACC CTTATCCTTT GTTGCCAGCG -GCTCGGCCG GGAACTCAAA GGAGACTGCC GGTGATAAAC

Pantoea_dispersa_(KY882077.1) ACCTTACCTG GCCTTGACAT CCAG--AGAA CTTAGCAGAG ATGCTTTGGT GCC-TTCGGG AA-CTCTGAG ACAGGTGCTG CATGGCTGTC GTCAGCTCGT GTTGTGAAAT GTTGGGTTAA GTCCCGCAAC GAGCGCAACC CTTATCCTTT GTTGCCAGCG -GNTCGGCCG GGAACTCAAA GGAGACTGCC GGTGATAAAC

PaKu21_-_Bacillus_sonorensis ACCTTACCAG GTCTTGACAT CCTC--TGAC ACCCCTAGAG ATAGGGC-TT CCCCTTCGGG GG-CAGAGTG ACAGGTGGTG CATGGTTGTC GTCAGCTCGT GTCGTGAGAT GTTGGGTTAA GTCCCGCAAC GAGCGCAACC CTTGATCTTA GTTGCCAGCA --TTCAGTTG GGCACTCTAA GGTGACTGCC GGTGACAAAC

Bacillus_sonorensis_(KU551167. ACCTTACCAG GTCTTGACAT CCTC--TGAC ACCCCTAGAG ATAGGGC-TT CCCCTTCGGG GG-CAGAGTG ACAGGTGGTG CATGGTTGTC GTCAGCTCGT GTCGTGAGAT GTTGGGTTAA GTCCCGCAAC GAGCGCAACC CTTGATCTTA GTTGCCAGCA --TTCAGTTG GGCACTCTAA GGTGACTGCC GGTGACAAAC

Bacillus_sonorensis_(FN397516. ACCTTACCAG GTCTTGACAT CCTC--TGAC ACCCCTAGAG ATAGGGC-TT CCCCTTCGGG GG-CAGAGTG ACAGGTGGTG CATGGTTGTC GTCAGCTCGT GTCGTGAGAT GTTGGGTTAA GTCCCGCAAC GAGCGCAACC CTTGATCTTA GTTGCCAGCA --TTCAGTTG GGCACTCTAA GGTGACTGCC GGTGACAAAC

Bacillus_sonorensis_(KU551137. ACCTTACCAG GTCTTGACAT CCTC--TGAC ACCCCTAGAG ATAGGGC-TT CCCCTTCGGG GG-CAGAGTG ACAGGTGGTG CATGGTTGTC GTCAGCTCGT GTCGTGAGAT GTTGGGTTAA GTCCCGCAAC GAGCGCAACC CTTGATCTTA GTTGCCAGCA --TTCAGTTG GGCACTCTAA GGTGACTGCC GGTGACAAAC

PaKu24_-_Bacillus_sonorensis ACCTTACCAG GTCTTGACAT CCTC--TGAC ACCCCTAGAG ATAGGGC-TT CCCCTTCGGG GG-CAGAGTG ACAGGTGGTG CATGGTTGTC GTCAGCTCGT GTCGTGAGAT GTTGGGTTAA GTCCCGCAAC GAGCGCAACC CTTGATCTTA GTTGCCAGCA --TTCAGTTG GGCACTCTAA GGTGACTGCC GGTGACAAAC

PaKu22_-_Bacillus_subtilis ACCTTACCAG GTCTTGACAT CCTC--TGAC AATCCTAGAG ATAGGAC-GT CCCCTTCGGG GG-CAGAGTG ACAGGTGGTG CATGGTTGTC GTCAGCTCGT GTCGTGAGAT GTTGGGTTAA GTCCCGCAAC GAGCGCAACC CTTGATCTTA GTTGCCAGCA --TTCAGTTG GGCACTCTAA GGTGACTGCC GGTGACAAAC

Bacillus_tequilensis_(MK880583 ACCTTACCAG GTCTTGACAT CCTC--TGAC AATCCTAGAG ATAGGAC-GT CCCCTTCGGG GG-CAGAGTG ACAGGTGGTG CATGGTTGTC GTCAGCTCGT GTCGTGAGAT GTTGGGTTAA GTCCCGCAAC GAGCGCAACC CTTGATCTTA GTTGCCAGCA --TTCAGTTG GGCACTCTAA GGTGACTGCC GGTGACAAAC

Bacillus_subtilis_(MK765023.1) ACCTTACCAG GTCTTGACAT CCTC--TGAC AATCCTAGAG ATAGGAC-GT CCCCTTCGGG GG-CAGAGTG ACAGGTGGTG CATGGTTGTC GTCAGCTCGT GTCGTGAGAT GTTGGGTTAA GTCCCGCAAC GAGCGCAACC CTTGATCTTA GTTGCCAGCA --TTCAGTTG GGCACTCTAA GGTGACTGCC GGTGACAAAC

PaKu23_-_Bacillus_subtilis ACCTTACCAG GTCTTGACAT CCTC--TGAC AATCCTAGAG ATAGGAC-GT CCCCTTCGGG GG-CAGAGTG ACAGGTGGTG CATGGTTGTC GTCAGCTCGT GTCGTGAGAT GTTGGGTTAA GTCCCGCAAC GAGCGCAACC CTTGATCTTA GTTGCCAGCA --TTCAGTTG GGCACTCTAA GGTGACTGCC GGTGACAAAC

Bacillus_subtilis_(MK511833.1) ACCTTACCAG GTCTTGACAT CCTC--TGAC AATCCTAGAG ATAGGAC-GT CCCCTTCGGG GG-CAGAGTG ACAGGTGGTG CATGGTTGTC GTCAGCTCGT GTCGTGAGAT GTTGGGTTAA GTCCCGCAAC GAGCGCAACC CTTGATCTTA GTTGCCAGCA --TTCAGTTG GGCACTCTAA GGTGACTGCC GGTGACAAAC

Bacillus_subtilis_(KX450400.1) ACCTTACCAG GTCTTGACAT CCTC--TGAC AATCCTAGAG ATAGGAC-GT CCCCTTCGGG GG-CAGAGTG ACAGGTGGTG CATGGTTGTC GTCAGCTCGT GTCGTGAGAT GTTGGGTTAA GTCCCGCAAC GAGCGCAACC CTTGATCTTA GTTGCCAGCA --TTCAGTTG GGCACTCTAA GGTGACTGCC GGTGACAAAC

PaKu25_-_Bacillus_cereus ACCTTACCAG GTCTTGACAT CCTC--TGAA AACCCTAGAG ATAGGGC-TT CTCCTTCGGG AG-CAGAGTG ACAGGTGGTG CATGGTTGTC GTCAGCTCGT GTCGTGAGAT GTTGGGTTAA GTCCCGCAAC GAGCGCAACC CTTGATCTTA GTTGCCATCA --TTAAGTTG GGCACTCTAA GGTGACTGCC GGTGACAAAC

Bacillus_thuringiensis_(CP0378 ACCTTACCAG GTCTTGACAT CCTC--TGAA AACCCTAGAG ATAGGGC-TT CTCCTTCGGG AG-CAGAGTG ACAGGTGGTG CATGGTTGTC GTCAGCTCGT GTCGTGAGAT GTTGGGTTAA GTCCCGCAAC GAGCGCAACC CTTGATCTTA GTTGCCATCA --TTAAGTTG GGCACTCTAA GGTGACTGCC GGTGACAAAC

Bacillus_mobilis_(CP031443.1) ACCTTACCAG GTCTTGACAT CCTC--TGAA AACCCTAGAG ATAGGGC-TT CTCCTTCGGG AG-CAGAGTG ACAGGTGGTG CATGGTTGTC GTCAGCTCGT GTCGTGAGAT GTTGGGTTAA GTCCCGCAAC GAGCGCAACC CTTGATCTTA GTTGCCATCA --TTAAGTTG GGCACTCTAA GGTGACTGCC GGTGACAAAC

Bacillus_thuringiensis_(CP0357 ACCTTACCAG GTCTTGACAT CCTC--TGAA AACCCTAGAG ATAGGGC-TT CTCCTTCGGG AG-CAGAGTG ACAGGTGGTG CATGGTTGTC GTCAGCTCGT GTCGTGAGAT GTTGGGTTAA GTCCCGCAAC GAGCGCAACC CTTGATCTTA GTTGCCATCA --TTAAGTTG GGCACTCTAA GGTGACTGCC GGTGACAAAC

Bacillus_cereus_(CP030982.1) ACCTTACCAG GTCTTGACAT CCTC--TGAA AACCCTAGAG ATAGGGC-TT CTCCTTCGGG AG-CAGAGTG ACAGGTGGTG CATGGTTGTC GTCAGCTCGT GTCGTGAGAT GTTGGGTTAA GTCCCGCAAC GAGCGCAACC CTTGATCTTA GTTGCCATCA --TTAAGTTG GGCACTCTAA GGTGACTGCC GGTGACAAAC

Bacillus_cereus_(MH068823.1) ACCTTACCAG GTCTTGACAT CCTC--TGAA AACCCTAGAG ATAGGGC-TT CTCCTTCGGG AG-CAGAGTG ACAGGTGGTG CATGGTTGTC GTCAGCTCGT GTCGTGAGAT GTTGGGTTAA GTCCCGCAAC GAGCGCAACC CTTGATCTTA GTTGCCATCA --TTAAGTTG GGCACTCTAA GGTGACTGCC GGTGACAAAC

Bacillus_thuringiensis_(MG7453 ACCTTACCAG GTCTTGACAT CCTC--TGAA AACCCTAGAG ATAGGGC-TT CTCCTTCGGG AG-CAGAGTG ACAGGTGGTG CATGGTTGTC GTCAGCTCGT GTCGTGAGAT GTTGGGTTAA GTCCCGCAAC GAGCGCAACC CTTGATCTTA GTTGCCATCA --TTAAGTTG GGCACTCTAA GGTGACTGCC GGTGACAAAC

Rhizobium_massiliae(Country-_T ACCTTACCAG CTCTTGACAT TCGGGGTATG GGCATTGGAG ACGATGTCCT TCAGTTAGGC TGGCCCCAGA ACAGGTGCTG CATGGCTGTC GTCAGCTCGT GTCGTGAGAT GTTGGGTTAA GTCCCGCAAC GAGCGCAACC CTCGCCCTTA GTTGCCAGCA --TTTAGTTG GGCACTCTAA GGGGACTGCC GGTGATAAGC

Stenotrophomonas_maltophilia_( ACCTTACCTG GCCTTGACAT GTCG--AGAA CTTTCCAGAG ATGGATTGGT GCC-TTCGGG AA-CTCGAAC ACAGGTGCTG CATGGCTGTC GTCAGCTCGT GTCGTGAGAT GTTGGGTTAA GTCCCGCAAC GAGCGCAACC CTTGTCCTTA GTTGCCAGCA CGTAATGGTG GGAACTCTAA GGAGACCGCC GGTGACAAAC

Serratia_sp._(Country-_Tunisia ACCTCACCTA CTTTTGACAT CCAG--AGAA CTCCCCAGAG ACGGACTGGT GCC-TTCGGG AA-CTCTGAG ACAGGTGCTG CATGGATGTC GTCAGCTTTT GTTGTGAAAT GTTGGGTTAA GTTCCGCAAC GAGTGCAACC CTTATCCCCC GCCGCCAGCG -GTTCGGCCG GGAATT---- ---------- ----------

Staphylococcus_saprophyticus_( ACCTTACCAA ATCTTGACAT CCTT--TGAA AACTCTAGAG ATAGAGCCTT CCCCTTCGGG GGACAAAGTG ACAGGTGGTG CATGGTTGTC GTCAGCTCGT GTCGTGAGAT GTTGGGTTAA GTCCCGCAAC GAGCGCAACC CTTAAGCTTA GTTGCCATCA --TTAAGTTG GGCACTCTAG GTTGACTGCC GGTGACAAAC

Staphylococcus_saprophyticus_( ACCTTACCAA ATCTTGACAT CCTT--TGAA AACTCTAGAG ATAGAGCCTT CCCCTTCGGG GGACAAAGTG ACAGGTGGTG CATGGTTGTC GTCAGCTCGT GTCGTGAGAT GTTGGGTTAA GTCCCGCAAC GAGCGCAACC CTTAAGCTTA GTTGCCATCA --TTAAGTTG GGCACTCTAG GTTGACTGCC GGTGACAAAC

Bacillus_subtilis_(Country-Ira ACCTTACCAG GTCTTGACAT CCTC--TGAC AATCCTAGAG ATAGGAC-GT CCCCTTCGGG GG-CAGAGTG ACAGGTGGTG CATGGTTGTC GTCAGCTCGT GTCGTGAGAT GTTGGGTTAA GTCCCGCAAC GAGCGCAACC CTTGATCTTA GTTGCCAGCA --TTCAGTTG GGCACTCTAA GGTGACTGCC GGTGACAAAC

Bacillus_cereus_(Country-UK)_( ACCTTACCAG GTCTTGACAT CCTC--TGAA AACCCTAGAG ATAGGGC-TT CTCCTTCGGG AG-CAGAGTG ACAGGTGGTG CATGGTTGTC GTCAGCTCGT GTCGTGAGAT GTTGGGTTAA GTCCCGCAAC GAGCGCAACC CTTGATCTTA GTTGCCATCA --TTAAGTTG GGCACTCTAA GGTGACTGCC GGTGACAAAC

....|....| ....|....| ....|....| ....|....| ....|....| ....|....| ....|....| ....|....| ....|....| ....|....| ....|....| ....|....| ....|....| ....|....| ....|....| ....|....| ....|....| ....|....| ....|....| ....|....|

1205 1215 1225 1235 1245 1255 1265 1275 1285 1295 1305 1315 1325 1335 1345 1355 1365 1375 1385 1395

PaKu1_-_Serratia_marcescens TG-GAGGAAG GTGGGGATGA CGTCAAGTCA TCATGGCCCT TACGAGTAGG GCTACACACG TGCTACAATG GCGTATACAA AGAGAAGCGA CCTCGCGAGA GCAAGCGGAC CTCATAAAGT ACGTCGTAGT CCGGATTGGA GTCTGCAACT CGACTCCATG AAGTCGGAAT CGCTAGTAAT CGTAGATCAG AAT-GCTACG

Serratia_marcescens_(KX911721. TG-GAGGAAG GTGGGGATGA CGTCAAGTCA TCATGGCCCT TACGAGTAGG GCTACACACG TGCTACAATG GCGTATACAA AGAGAAGCGA CCTCGCGAGA GCAAGCGGAC CTCATAAAGT ACGTCGTAGT CCGGATTGGA GTCTGCAACT CGACTCCATG AAGTCGGAAT CGCTAGTAAT CGTAGATCAG AAT-GCTACG

Serratia_marcescens_(KT992361. TG-GAGGAAG GTGGGGATGA CGTCAAGTCA TCATGGCCCT TACGAGTAGG GCTACACACG TGCTACAATG GCGTATACAA AGAGAAGCGA CCTCGCGAGA GCAAGCGGAC CTCATAAAGT ACGTCGTAGT CCGGATTGGA GTCTGCAACT CGACTCCATG AAGTCGGAAT CGCTAGTAAT CGTAGATCAG AAT-GCTACG

PaKu2_-_Enterobacter_sp. TG-GAGGAAG GTGGGGATGA CGTCAAGTCA TCATGGCCCT TACGAGTAGG GCTACACACG TGCTACAATG GCGCATACAA AGAGAAGCGA CCTCGCGAGA GCAAGCGGAC CTCATAAAGT GCGTCGTAGT CCGGATTGGA GTCTGCAACT CGACTCCATG AAGTCGGAAT CGCTAGTAAT CGTGGATCAG AAT-GCCACG

Enterobacter_sp._(MG280962.1) TG-GAGGAAG GTGGGGATGA CGTCAAGTCA TCATGGCCCT TACGAGTAGG GCTACACACG TGCTACAATG GCGCATACAA AGAGAAGCGA CCTCGCGAGA GCAAGCGGAC CTCATAAAGT GCGTCGTAGT CCGGATTGGA GTCTGCAACT CGACTCCATG AAGTCGGAAT CGCTAGTAAT CGTGGATCAG AAT-GCCACG

Enterobacter_hormaechei_(CP029 TG-GAGGAAG GTGGGGATGA CGTCAAGTCA TCATGGCCCT TACGAGTAGG GCTACACACG TGCTACAATG GCGCATACAA AGAGAAGCGA CCTCGCGAGA GCAAGCGGAC CTCATAAAGT GCGTCGTAGT CCGGATTGGA GTCTGCAACT CGACTCCATG AAGTCGGAAT CGCTAGTAAT CGTGGATCAG AAT-GCCACG

PaKu3_-_Staphylococcus_saproph CG-GAGGAAG GTGGGGATGA CGTCAAATCA TCATGCCCCT TATGATTTGG GCTACACACG TGCTACAATG GACAATACAA AGGGCAGCTA AACCGCGAGG TCATGCAAAT CCCATAAAGT TGTTCTCAGT TCGGATTGTA GTCTGCAACT CGACTACATG AAGCTGGAAT CGCTAGTAAT CGTAGATCAG CAT-GCTACG

Staphylococcus_saprophyticus_( CG-GAGGAAG GTGGGGATGA CGTCAAATCA TCATGCCCCT TATGATTTGG GCTACACACG TGCTACAATG GACAATACAA AGGGCAGCTA AACCGCGAGG TCATGCAAAT CCCATAAAGT TGTTCTCAGT TCGGATTGTA GTCTGCAACT CGACTACATG AAGCTGGAAT CGCTAGTAAT CGTAGATCAG CAT-GCTACG

Staphylococcus_saprophyticus_( CG-GAGGAAG GTGGGGATGA CGTCAAATCA TCATGCCCCT TATGATTTGG GCTACACACG TGCTACAATG GACAATACAA AGGGCAGCTA AACCGCGAGG TCATGCAAAT CCCATAAAGT TGTTCTCAGT TCGGATTGTA GTCTGCAACT CGACTACATG AAGCTGGAAT CGCTAGTAAT CGTAGATCAG CAT-GCTACG

PaKu4_-_Enterobacter_cloacae TG-GAGGAAG GTGGGGATGA CGTCAAGTCA TCATGGCCCT TACGACCAGG GCTACACACG TGCTACAATG GCGCATACAA AGAGAAGCGA CCTCGCGAGA GCAAGCGGAC CTCATAAAGT GCGTCGTAGT CCGGATTGGA GTCTGCAACT CGACTCCATG AAGTCGGAAT CGCTAGTAAT CGTAGATCAG AAT-GCTACG

Uncultured_Enterobacter_sp._(M TG-GAGGAAG GTGGGGATGA CGTCAAGTCA TCATGGCCCT TACGACCAGG GCTACACACG TGCTACAATG GCGCATACAA AGAGAAGCGA CCTCGCGAGA GCAAGCGGAC CTCATAAAGT GCGTCGTAGT CCGGATTGGA GTCTGCAACT CGACTCCATG AAGTCGGAAT CGCTAGTAAT CGTAGATCAG AAT-GCTACG

Enterobacter_cloacae_(KY930709 TG-GAGGAAG GTGGGGATGA CGTCAAGTCA TCATGGCCCT TACGACCAGG GCTACACACG TGCTACAATG GCGCATACAA AGAGAAGCGA CCTCGCGAGA GCAAGCGGAC CTCATAAAGT GCGTCGTAGT CCGGATTGGA GTCTGCAACT CGACTCCATG AAGTCGGAAT CGCTAGTAAT CGTAGATCAG AAT-GCTACG

PaKu5_-_Enterobacter_cloacae TG-GAGGAAG GTGGGGATGA CGTCAAGTCA TCATGGCCCT TACGACCAGG GCTACACACG TGCTACAATG GCGCATACAA AGAGAAGCGA CCTCGCGAGA GCAAGCGGAC CTCATAAAGT GCGTCGTAGT CCGGATTGGA GTCTGCAACT CGACTCCATG AAGTCGGAAT CGCTAGTAAT CGTAGATCAG AAT-GCTACG

Enterobacter_sp._(KU867838.1) TG-GAGGAAG GTGGGGATGA CGTCAAGTCA TCATGGCCCT TACGACCAGG GCTACACACG TGCTACAATG GCGCATACAA AGAGAAGCGA CCTCGCGAGA GCAAGCGGAC CTCATAAAGT GCGTCGTAGT CCGGATTGGA GTCTGCAACT CGACTCCATG AAGTCGGAAT CGCTAGTAAT CGTAGATCAG AAT-GCTACG

Enterobacter_cloacae_(KX242265 TG-GAGGAAG GTGGGGATGA CGTCAAGTCA TCATGGCCCT TACGACCAGG GCTACACACG TGCTACAATG GCGCATACAA AGAGAAGCGA CCTCGCGAGA GCAAGCGGAC CTCATAAAGT GCGTCGTAGT CCGGATTGGA GTCTGCAACT CGACTCCATG AAGTCGGAAT CGCTAGTAAT CGTAGATCAG AAT-GCTACG

Enterobacter_cloacae_(KP794925 TG-GAGGAAG GTGGGGATGA CGTCAAGTCA TCATGGCCCT TACGACCAGG GCTACACACG TGCTACAATG GCGCATACAA AGAGAAGCGA CCTCGCGAGA GCAAGCGGAC CTCATAAAGT GCGTCGTAGT CCGGATTGGA GTCTGCAACT CGACTCCATG AAGTCGGAAT CGCTAGTAAT CGTAGATCAG AAT-GCTACG

PaKu6_-_Staphylococcus_sciuri CG-GAGGAAG GTGGGGATGA CGTCAAATCA TCATGCCCCT TATGATTTGG GCTACACACG TGCTACAATG GATAATACAA AGGGCAGCGA ATCCGCGAGG CCAAGCAAAT CCCATAAAAT TATTCTCAGT TCGGATTGTA GTCTGCAACT CGACTACATG AAGCTGGAAT CGCTAGTAAT CGTAGATCAG CAT-GCTACG

Staphylococcus_sciuri_(MG70600 CG-GAGGAAG GTGGGGATGA CGTCAAATCA TCATGCCCCT TATGATTTGG GCTACACACG TGCTACAATG GATAATACAA AGGGCAGCGA ATCCGCGAGG CCAAGCAAAT CCCATAAAAT TATTCTCAGT TCGGATTGTA GTCTGCAACT CGACTACATG AAGCTGGAAT CGCTAGTAAT CGTAGATCAG CAT-GCTACG

Staphylococcus_lentus_(MG98829 CG-GAGGAAG GTGGGGATGA CGTCAAATCA TCATGCCCCT TATGATTTGG GCTACACACG TGCTACAATG GATAATACAA AGGGCAGCGA ATCCGCGAGG CCAAGCAAAT CCCATAAAAT TATTCTCAGT TCGGATTGTA GTCTGCAACT CGACTACATG AAGCTGGAAT CGCTAGTAAT CGTAGATCAG CAT-GCTACG

Staphylococcus_sciuri_(MG51742 CG-GAGGAAG GTGGGGATGA CGTCAAATCA TCATGCCCCT TATGATTTGG GCTACACACG TGCTACAATG GATAATACAA AGGGCAGCGA ATCCGCGAGG CCAAGCAAAT CCCATAAAAT TATTCTCAGT TCGGATTGTA GTCTGCAACT CGACTACATG AAGCTGGAAT CGCTAGTAAT CGTAGATCAG CAT-GCTACG

PaKu8_-_Pseudomonas_aeruginosa CG-GAGGAAG GTGGGGATGA CGTCAAGTCA TCATGGCCCT TACGGCCAGG GCTACACACG TGCTACAATG GTCGGTACAA AGGGTTGCCA AGCCGCGAGG TGGAGCTAAT CCCATAAAAC CGATCGTAGT CCGGATCGCA GTCTGCAACT CGACTGCGTG AAGTCGGAAT CGCTAGTAAT CGTGAATCAG AAT-GTCACG

Uncultured_bacterium_(FM996243 CG-GAGGAAG GTGGGGATGA CGTCAAGTCA TCATGGCCCT TACGGCCAGG GCTACACACG TGCTACAATG GTCGGTACAA AGGGTTGCCA AGCCGCGAGG TGGAGCTAAT CCCATAAAAC CGATCGTAGT CCGGATCGCA GTCTGCAACT CGACTGCGTG AAGTCGGAAT CGCTAGTAAT CGTGAATCAG AAT-GTCACG

Pseudomonas_aeruginosa_(MK4304 CG-GAGGAAG GTGGGGATGA CGTCAAGTCA TCATGGCCCT TACGGCCAGG GCTACACACG TGCTACAATG GTCGGTACAA AGGGTTGCCA AGCCGCGAGG TGGAGCTAAT CCCATAAAAC CGATCGTAGT CCGGATCGCA GTCTGCAACT CGACTGCGTG AAGTCGGAAT CGCTAGTAAT CGTGAATCAG AAT-GTCACG

PaKu9_-_Staphylococcus_arletta CG-GAGGAAG GTGGGGATGA CGTCAAATCA TCATGCCCCT TATGATTTGG GCTACACACG TGCTACAATG GACAATACAA AGGGCAGCTA AACCGCGAGG TCATGCAAAT CCCATAAAGT TGTTCTCAGT TCGGATTGTA GTCTGCAACT CGACTACATG AAGCTGGAAT CGCTAGTAAT CGTAGATCAG CAT-GCTACG

Staphylococcus_arlettae_(KX344 CG-GAGGAAG GTGGGGATGA CGTCAAATCA TCATGCCCCT TATGATTTGG GCTACACACG TGCTACAATG GACAATACAA AGGGCAGCTA AACCGCGAGG TCATGCAAAT CCCATAAAGT TGTTCTCAGT TCGGATTGTA GTCTGCAACT CGACTACATG AAGCTGGAAT CGCTAGTAAT CGTAGATCAG CAT-GCTACG

Staphylococcus_sp._(KU891836.1 CG-GAGGAAG GTGGGGATGA CGTCAAATCA TCATGCCCCT TATGATTTGG GCTACACACG TGCTACAATG GACAATACAA AGGGCAGCTA AACCGCGAGG TCATGCAAAT CCCATAAAGT TGTTCTCAGT TCGGATTGTA GTCTGCAACT CGACTACATG AAGCTGGAAT CGCTAGTAAT CGTAGATCAG CAT-GCTACG

PaKu10_-_Aeromonas_caviae CG-GAGGAAG GTGGGGATGA CGTCAAGTCA TCATGGCCCT TACGGCCAGG GCTACACACG TGCTACAATG GCGCGTACAG AGGGCTGCAA GCTAGCGATA GTGAGCGAAT CCCAAAAAGC GCGTCGTAGT CCGGATTGGA GTCTGCAACT CGACTCCATG AAGTCGGAAT CGCTAGTAAT CGCAAATCAG AAT-GTTGCG

Uncultured_bacterium_(KX242397 CG-GAGGAAG GTGGGGATGA CGTCAAGTCA TCATGGCCCT TACGGCCAGG GCTACACACG TGCTACAATG GCGCGTACAG AGGGCTGCAA GCTAGCGATA GTGAGCGAAT CCCAAAAAGC GCGTCGTAGT CCGGATTGGA GTCTGCAACT CGACTCCATG AAGTCGGAAT CGCTAGTAAT CGCAAATCAG AAT-GTTGCG

Aeromonas_caviae_(HQ407268.1) CG-GAGGAAG GTGGGGATGA CGTCAAGTCA TCATGGCCCT TACGGCCAGG GCTACACACG TGCTACAATG GCGCGTACAG AGGGCTGCAA GCTAGCGATA GTGAGCGAAT CCCAAAAAGC GCGTCGTAGT CCGGATTGGA GTCTGCAACT CGACTCCATG AAGTCGGAAT CGCTAGTAAT CGCAAATCAG AAT-GTTGCG

Aeromonas_aquariorum_(EU085557 CG-GAGGAAG GTGGGGATGA CGTCAAGTCA TCATGGCCCT TACGGCCAGG GCTACACACG TGCTACAATG GCGCGTACAG AGGGCTGCAA GCTAGCGATA GTGAGCGAAT CCCAAAAAGC GCGTCGTAGT CCGGATTGGA GTCTGCAACT CGACTCCATG AAGTCGGAAT CGCTAGTAAT CGCAAATCAG AAT-GTTGCG

PaKu11_-_Aeromonas_caviae CG-GAGGAAG GTGGGGATGA CGTCAAGTCA TCATGGCCCT TACGGCCAGG GCTACACACG TGCTACAATG GCGCGTACAG AGGGCTGCAA GCTAGCGATA GTGAGCGAAT CCCAAAAAGC GCGTCGTAGT CCGGATTGGA GTCTGCAACT CGACTCCATG AAGTCGGAAT CGCTAGTAAT CGCAAATCAG AAT-GTTGCG

Aeromonas_caviae_(KY087979.1) CG-GAGGAAG GTGGGGATGA CGTCAAGTCA TCATGGCCCT TACGGCCAGG GCTACACACG TGCTACAATG GCGCGTACAG AGGGCTGCAA GCTAGCGATA GTGAGCGAAT CCCAAAAAGC GCGTCGTAGT CCGGATTGGA GTCTGCAACT CGACTCCATG AAGTCGGAAT CGCTAGTAAT CGCAAATCAG AAT-GTTGCG

Uncultured_Aeromonas_sp._(KU94 CG-GAGGAAG GTGGGGATGA CGTCAAGTCA TCATGGCCCT TACGGCCAGG GCTACACACG TGCTACAATG GCGCGTACAG AGGGCTGCAA GCTAGCGATA GTGAGCGAAT CCCAAAAAGC GCGTCGTAGT CCGGATTGGA GTCTGCAACT CGACTCCATG AAGTCGGAAT CGCTAGTAAT CGCAAATCAG AAT-GTTGCG

PaKu12_-_Staphylococcus_warner CG-GAGGAAG GTGGGGATGA CGTCAAATCA TCATGCCCCT TATGATTTGG GCTACACACG TGCTACAATG GACAATACAA AGGGCAGCTA AACCGCGAGG TCAAGCAAAT CCCATAAAGT TGTTCTCAGT TCGGATTGTA GTCTGCAACT CGACTACATG AAGCTGGAAT CGCTAGTAAT CGTAGATCAG CAT-GCTACG

Staphylococcus_warneri_(MH0149 CG-GAGGAAG GTGGGGATGA CGTCAAATCA TCATGCCCCT TATGATTTGG GCTACACACG TGCTACAATG GACAATACAA AGGGCAGCTA AACCGCGAGG TCAAGCAAAT CCCATAAAGT TGTTCTCAGT TCGGATTGTA GTCTGCAACT CGACTACATG AAGCTGGAAT CGCTAGTAAT CGTAGATCAG CAT-GCTACG

Staphylococcus_warneri_(KF3063 CG-GAGGAAG GTGGGGATGA CGTCAAATCA TCATGCCCCT TATGATTTGG GCTACACACG TGCTACAATG GACAATACAA AGGGCAGCTA AACCGCGAGG TCAAGCAAAT CCCATAAAGT TGTTCTCAGT TCGGATTGTA GTCTGCAACT CGACTACATG AAGCTGGAAT CGCTAGTAAT CGTAGATCAG CAT-GCTACG

PaKu13_-_Bacillus_megaterium CG-GAGGAAG GTGGGGATGA CGTCAAATCA TCATGCCCCT TATGACCTGG GCTACACACG TGCTACAATG GATGGTACAA AGGGCTGCAA GACCGCGAGG TCAAGCCAAT CCCATAAAAC CATTCTCAGT TCGGATTGTA GGCTGCAACT CGCCTACATG AAGCTGGAAT CGCTAGTAAT CGCGGATCAG CAT-GCCGCG

Bacillus_sp._(MK691444.1) CG-GAGGAAG GTGGGGATGA CGTCAAATCA TCATGCCCCT TATGACCTGG GCTACACACG TGCTACAATG GATGGTACAA AGGGCTGCAA GACCGCGAGG TCAAGCCAAT CCCATAAAAC CATTCTCAGT TCGGATTGTA GGCTGCAACT CGCCTACATG AAGCTGGAAT CGCTAGTAAT CGCGGATCAG CAT-GCCGCG

Bacillus_sp._(LC373523.1) CG-GAGGAAG GTGGGGATGA CGTCAAATCA TCATGCCCCT TATGACCTGG GCTACACACG TGCTACAATG GATGGTACAA AGGGCTGCAA GACCGCGAGG TCAAGCCAAT CCCATAAAAC CATTCTCAGT TCGGATTGTA GGCTGCAACT CGCCTACATG AAGCTGGAAT CGCTAGTAAT CGCGGATCAG CAT-GCCGCG

Bacillus_aryabhattai_(MK474942 CG-GAGGAAG GTGGGGATGA CGTCAAATCA TCATGCCCCT TATGACCTGG GCTACACACG TGCTACAATG GATGGTACAA AGGGCTGCAA GACCGCGAGG TCAAGCCAAT CCCATAAAAC CATTCTCAGT TCGGATTGTA GGCTGCAACT CGCCTACATG AAGCTGGAAT CGCTAGTAAT CGCGGATCAG CAT-GCCGCG

Bacillus_aryabhattai_(MK474941 CG-GAGGAAG GTGGGGATGA CGTCAAATCA TCATGCCCCT TATGACCTGG GCTACACACG TGCTACAATG GATGGTACAA AGGGCTGCAA GACCGCGAGG TCAAGCCAAT CCCATAAAAC CATTCTCAGT TCGGATTGTA GGCTGCAACT CGCCTACATG AAGCTGGAAT CGCTAGTAAT CGCGGATCAG CAT-GCCGCG

Bacillus_megaterium_(KX298860. CG-GAGGAAG GTGGGGATGA CGTCAAATCA TCATGCCCCT TATGACCTGG GCTACACACG TGCTACAATG GATGGTACAA AGGGCTGCAA GACCGCGAGG TCAAGCCAAT CCCATAAAAC CATTCTCAGT TCGGATTGTA GGCTGCAACT CGCCTACATG AAGCTGGAAT CGCTAGTAAT CGCGGATCAG CAT-GCCGCG

PaKu14_-_Pseudomonas_stutzeri CG-GAGGAAG GTGGGGATGA CGTCAAGTCA TCATGGCCCT TACGGCCTGG GCTACACACG TGCTACAATG GTCGGTACAA AGGGTTGCCA AGCCGCGAGG TGGAGCTAAT CCCATAAAAC CGATCGTAGT CCGGATCGCA GTCTGCAACT CGACTGCGTG AAGTCGGAAT CGCTAGTAAT CGTGAATCAG AAT-GTCACG

Pseudomonas_stutzeri_(MG892782 CG-GAGGAAG GTGGGGATGA CGTCAAGTCA TCATGGCCCT TACGGCCTGG GCTACACACG TGCTACAATG GTCGGTACAA AGGGTTGCCA AGCCGCGAGG TGGAGCTAAT CCCATAAAAC CGATCGTAGT CCGGATCGCA GTCTGCAACT CGACTGCGTG AAGTCGGAAT CGCTAGTAAT CGTGAATCAG AAT-GTCACG

Pseudomonas_stutzeri_(MG595371 CG-GAGGAAG GTGGGGATGA CGTCAAGTCA TCATGGCCCT TACGGCCTGG GCTACACACG TGCTACAATG GTCGGTACAA AGGGTTGCCA AGCCGCGAGG TGGAGCTAAT CCCATAAAAC CGATCGTAGT CCGGATCGCA GTCTGCAACT CGACTGCGTG AAGTCGGAAT CGCTAGTAAT CGTGAATCAG AAT-GTCACG

PaKu15_-_Rhizobium_sp. CGAGAGGAAG GTGGGGATGA CGTCAAGTCC TCATGGCCCT TACGGGCTGG GCTACACACG TGCTACAATG GTGGTGACAG TGGGCAGCGA GACAGCGATG TCGAGCTAAT CTCCAAAAGC CA-TCTCAGT TCGGATTGCA CTCTGCAACT CGAGTGCATG AAGTTGGAAT CGCTAGTAAT CGCAGATCAG CAT-GCTGCG

Rhizobium_pusense_(CP039895.1) CGAGAGGAAG GTGGGGATGA CGTCAAGTCC TCATGGCCCT TACGGGCTGG GCTACACACG TGCTACAATG GTGGTGACAG TGGGCAGCGA GACAGCGATG TCGAGCTAAT CTCCAAAAGC CA-TCTCAGT TCGGATTGCA CTCTGCAACT CGAGTGCATG AAGTTGGAAT CGCTAGTAAT CGCAGATCAG CAT-GCTGCG

Rhizobium_sp._(KY971009.1) CGAGAGGAAG GTGGGGATGA CGTCAAGTCC TCATGGCCCT TACGGGCTGG GCTACACACG TGCTACAATG GTGGTGACAG TGGGCAGCGA GACAGCGATG TCGAGCTAAT CTCCAAAAGC CA-TCTCAGT TCGGATTGCA CTCTGCAACT CGAGTGCATG AAGTTGGAAT CGCTAGTAAT CGCAGATCAG CAT-GCTGCG

PaKu7_-_Stenotrophomonas_malto CG-GAGGAAG GTGGGGATGA CGTCAAGTCA TCATGGCCCT TACGGCCAGG GCTACACACG TACTACAATG GTAGGGACAG AGGGCTGCAA GCCGGCGACG GTAAGCCAAT CCCAGAAACC CTATCTCAGT CCGGATTGGA GTCTGCAACT CGACTCCATG AAGTCGGAAT CGCTAGTAAT CGCAGATCAG CATTGCTGCG

Stenotrophomonas_maltophilia_( CG-GAGGAAG GTGGGGATGA CGTCAAGTCA TCATGGCCCT TACGGCCAGG GCTACACACG TACTACAATG GTAGGGACAG AGGGCTGCAA GCCGGCGACG GTAAGCCAAT CCCAGAAACC CTATCTCAGT CCGGATTGGA GTCTGCAACT CGACTCCATG AAGTCGGAAT CGCTAGTAAT CGCAGATCAG CATTGCTGCG

Stenotrophomonas_maltophilia_( CG-GAGGAAG GTGGGGATGA CGTCAAGTCA TCATGGCCCT TACGGCCAGG GCTACACACG TACTACAATG GTAGGGACAG AGGGCTGCAA GCCGGCGACG GTAAGCCAAT CCCAGAAACC CTATCTCAGT CCGGATTGGA GTCTGCAACT CGACTCCATG AAGTCGGAAT CGCTAGTAAT CGCAGATCAG CATTGCTGCG

PaKu16_-_Stenotrophomonas_malt CG-GAGGAAG GTGGGGATGA CGTCAAGTCA TCATGGCCCT TACGGCCAGG GCTACACACG TACTACAATG GTAGGGACAG AGGGCTGCAA GCCGGCGACG GTAAGCCAAT CCCAGAAACC CTATCTCAGT CCGGATTGGA GTCTGCAACT CGACTCCATG AAGTCGGAAT CGCTAGTAAT CGCAGATCAG CATTGCTGCG

Stenotrophomonas_maltophilia_( CG-GAGGAAG GTGGGGATGA CGTCAAGTCA TCATGGCCCT TACGGCCAGG GCTACACACG TACTACAATG GTAGGGACAG AGGGCTGCAA GCCGGCGACG GTAAGCCAAT CCCAGAAACC CTATCTCAGT CCGGATTGGA GTCTGCAACT CGACTCCATG AAGTCGGAAT CGCTAGTAAT CGCAGATCAG CATTGCTGCG

Stenotrophomonas_maltophilia_( CG-GAGGAAG GTGGGGATGA CGTCAAGTCA TCATGGCCCT TACGGCCAGG GCTACACACG TACTACAATG GTAGGGACAG AGGGCTGCAA GCCGGCGACG GTAAGCCAAT CCCAGAAACC CTATCTCAGT CCGGATTGGA GTCTGCAACT CGACTCCATG AAGTCGGAAT CGCTAGTAAT CGCAGATCAG CATTGCTGCG

PaKu17_-_Stenotrophomonas_malt CG-GAGGAAG GTGGGGATGA CGTCAAGTCA TCATGGCCCT TACGGCCAGG GCTACACACG TACTACAATG GTAGGGACAG AGGGCTGCAA GCCGGCGACG GTAAGCCAAT CCCAGAAACC CTATCTCAGT CCGGATTGGA GTCTGCAACT CGACTCCATG AAGTCGGAAT CGCTAGTAAT CGCAGATCAG CATTGCTGCG

PaKu26_-_Stenotrophomonas_malt CG-GAGGAAG GTGGGGATGA CGTCAAGTCA TCATGGCCCT TACGGCCAGG GCTACACACG TACTACAATG GTAGGGACAG AGGGCTGCAA GCCGGCGACG GTAAGCCAAT CCCAGAAACC CTATCTCAGT CCGGATTGGA GTCTGCAACT CGACTCCATG AAGTCGGAAT CGCTAGTAAT CGCAGATCAG CATTGCTGCG

Stenotrophomonas_maltophilia_( CG-GAGGAAG GTGGGGATGA CGTCAAGTCA TCATGGCCCT TACGGCCAGG GCTACACACG TACTACAATG GTAGGGACAG AGGGCTGCAA GCCGGCGACG GTAAGCCAAT CCCAGAAACC CTATCTCAGT CCGGATTGGA GTCTGCAACT CGACTCCATG AAGTCGGAAT CGCTAGTAAT CGCAGATCAG CATTGCTGCG

PaKu18_-_Stenotrophomonas_pana CG-GAGGAAG GTGGGGATGA CGTCAAGTCA TCATGGCCCT TACGACCAGG GCTACACACG TACTACAATG GTAGGGACAG AGGGCTGCAA TCCCGCGAGG GTGAGCCAAT CCCAGAAACC CTATCTCAGT CCGGATTGGA GTCTGCAACT CGACTCCATG AAGTCGGAAT CGCTAGTAAT CGCAGATCAG CATTGCTGCG

Stenotrophomonas_panacihumi_(M CG-GAGGAAG GTGGGGATGA CGTCAAGTCA TCATGGCCCT TACGACCAGG GCTACACACG TACTACAATG GTAGGGACAG AGGGCTGCAA TCCCGCGAGG GTGAGCCAAT CCCAGAAACC CTATCTCAGT CCGGATTGGA GTCTGCAACT CGACTCCATG AAGTCGGAAT CGCTAGTAAT CGCAGATCAG CATTGCTGCG

Stenotrophomonas_panacihumi_(K CG-GAGGAAG GTGGGGATGA CGTCAAGTCA TCATGGCCCT TACGACCAGG GCTACACACG TACTACAATG GTAGGGACAG AGGGCTGCAA TCCCGCGAGG GTGAGCCAAT CCCAGAAACC CTATCTCAGT CCGGATTGGA GTCTGCAACT CGACTCCATG AAGTCGGAAT CGCTAGTAAT CGCAGATCAG CATTGCTGCG

PaKu19_-_Bacillus_licheniformi CG-GAGGAAG GTGGGGATGA CGTCAAATCA TCATGCCCCT TATGACCTGG GCTACACACG TGCTACAATG GGCAGAACAA AGGGCAGCGA AGCCGCGAGG CTAAGCCAAT CCCACAAATC TGTTCTCAGT TCGGATCGCA GTCTGCAACT CGACTGCGTG AAGCTGGAAT CGCTAGTAAT CGCGGATCAG CAT-GCCGCG

Bacillus_licheniformis_(KJ5722 CG-GAGGAAG GTGGGGATGA CGTCAAATCA TCATGCCCCT TATGACCTGG GCTACACACG TGCTACAATG GGCAGAACAA AGGGCAGCGA AGCCGCGAGG CTAAGCCAAT CCCACAAATC TGTTCTCAGT TCGGATCGCA GTCTGCAACT CGACTGCGTG AAGCTGGAAT CGCTAGTAAT CGCGGATCAG CAT-GCCGCG

Bacillus_licheniformis_(KT1536 CG-GAGGAAG GTGGGGATGA CGTCAAATCA TCATGCCCCT TATGACCTGG GCTACACACG TGCTACAATG GGCAGAACAA AGGGCAGCGA AGCCGCGAGG CTAAGCCAAT CCCACAAATC TGTTCTCAGT TCGGATCGCA GTCTGCAACT CGACTGCGTG AAGCTGGAAT CGCTAGTAAT CGCGGATCAG CAT-GCCGCG

PaKu20_-__Pantoea_dispersa CG-GAGGAAG GTGGGGATGA CGTCAAGTCA TCATGGCCCT TACGGCCAGG GCTACACACG TGCTACAATG GCGCATACAA AGAGAAGCGA CCTCGCGAGA GCAAGCGGAC CTCATAAAGT GCGTCGTAGT CCGGATTGGA GTCTGCAACT CGACTCCATG AAGTCGGAAT CGCTAGTAAT CGTAGATCAG AAT-GCTACG

Pantoea_dispersa_(KY292463.1) CG-GAGGAAG GTGGGGATGA CGTCAAGTCA TCATGGCCCT TACGGCCAGG GCTACACACG TGCTACAATG GCGCATACAA AGAGAAGCGA CCTCGCGAGA GCAAGCGGAC CTCATAAAGT GCGTCGTAGT CCGGATTGGA GTCTGCAACT CGACTCCATG AAGTCGGAAT CGCTAGTAAT CGTAGATCAG AAT-GCTACG

Pantoea_dispersa_(KY882077.1) CG-GAGGAAG GTGGGGATGA CGTCAAGTCA TCATGGCCCT TACGGCCAGG GCTACACACG TGCTACAATG GCGCATACAA AGAGAAGCGA CCTCGCGAGA GCAAGCGGAC CTCATAAAGT GCGTCGTAGT CCGGATTGGA GTCTGCAACT CGACTCCATG AAGTCGGAAT CGCTAGTAAT CGTAGATCAG AAT-GCTACG

PaKu21_-_Bacillus_sonorensis CG-GAGGAAG GTGGGGATGA CGTCAAATCA TCATGCCCCT TATGACCTGG GCTACACACG TGCTACAATG GGCAGAACAA AGGGCAGCGA AACCGCGAGG CTAAGCCAAT CCCACAAATC TGCTCTCAGT TCGGATCGCA GTCTGCAACT CGACTGCGTG AAGCTGGAAT CGCTAGTAAT CGCGGATCAG CAT-GCCGCG

Bacillus_sonorensis_(KU551167. CG-GAGGAAG GTGGGGATGA CGTCAAATCA TCATGCCCCT TATGACCTGG GCTACACACG TGCTACAATG GGCAGAACAA AGGGCAGCGA AGCCGCGAGG CTAAGCCAAT CCCACAAATC TGCTCTCAGT TCGGATCGCA GTCTGCAACT CGACTGCGTG AAGCTGGAAT CGCTAGTAAT CGCGGATCAG CAT-GCCGCG

Bacillus_sonorensis_(FN397516. CG-GAGGAAG GTGGGGATGA CGTCAAATCA TCATGCCCCT TATGACCTGG GCTACACACG TGCTACAATG GGCAGAACAA AGGGCAGCGA AGCCGCGAGG CTAAGCCAAT CCCACAAATC TGCTCTCAGT TCGGATCGCA GTCTGCAACT CGACTGCGTG AAGCTGGAAT CGCTAGTAAT CGCGGATCAG CAT-GCCGCG

Bacillus_sonorensis_(KU551137. CG-GAGGAAG GTGGGGATGA CGTCAAATCA TCATGCCCCT TATGACCTGG GCTACACACG TGCTACAATG GGCAGAACAA AGGGCAGCGA AGCCGCGAGG CTAAGCCAAT CCCACAAATC TGCTCTCAGT TCGGATCGCA GTCTGCAACT CGACTGCGTG AAGCTGGAAT CGCTAGTAAT CGCGGATCAG CAT-GCCGCG

PaKu24_-_Bacillus_sonorensis CG-GAGGAAG GTGGGGATGA CGTCAAATCA TCATGCCCCT TATGACCTGG GCTACACACG TGCTACAATG GGCAGAACAA AGGGCAGCGA AACCGCGAGG CTAAGCCAAT CCCACAAATC TGCTCTCAGT TCGGATCGCA GTCTGCAACT CGACTGCGTG AAGCTGGAAT CGCTAGTAAT CGCGGATCAG CAT-GCCGCG

PaKu22_-_Bacillus_subtilis CG-GAGGAAG GTGGGGATGA CGTCAAATCA TCATGCCCCT TATGACCTGG GCTACACACG TGCTACAATG GACAGAACAA AGGGCAGCGA AACCGCGAGG TTAAGCCAAT CCCACAAATC TGTTCTCAGT TCGGATCGCA GTCTGCAACT CGACTGCGTG AAGCTGGAAT CGCTAGTAAT CGCGGATCAG CAT-GCCGCG

Bacillus_tequilensis_(MK880583 CG-GAGGAAG GTGGGGATGA CGTCAAATCA TCATGCCCCT TATGACCTGG GCTACACACG TGCTACAATG GACAGAACAA AGGGCAGCGA AACCGCGAGG TTAAGCCAAT CCCACAAATC TGTTCTCAGT TCGGATCGCA GTCTGCAACT CGACTGCGTG AAGCTGGAAT CGCTAGTAAT CGCGGATCAG CAT-GCCGCG

Bacillus_subtilis_(MK765023.1) CG-GAGGAAG GTGGGGATGA CGTCAAATCA TCATGCCCCT TATGACCTGG GCTACACACG TGCTACAATG GACAGAACAA AGGGCAGCGA AACCGCGAGG TTAAGCCAAT CCCACAAATC TGTTCTCAGT TCGGATCGCA GTCTGCAACT CGACTGCGTG AAGCTGGAAT CGCTAGTAAT CGCGGATCAG CAT-GCCGCG

PaKu23_-_Bacillus_subtilis CG-GAGGAAG GTGGGGATGA CGTCAAATCA TCATGCCCCT TATGACCTGG GCTACACACG TGCTACAATG GACAGAACAA AGGGCAGCGA AACCGCGAGG TTAAGCCAAT CCCACAAATC TGTTCTCAGT TCGGATCGCA GTCTGCAACT CGACTGCGTG AAGCTGGAAT CGCTAGTAAT CGCGGATCAG CAT-GCCGCG

Bacillus_subtilis_(MK511833.1) CG-GAGGAAG GTGGGGATGA CGTCAAATCA TCATGCCCCT TATGACCTGG GCTACACACG TGCTACAATG GACAGAACAA AGGGCAGCGA AACCGCGAGG TTAAGCCAAT CCCACAAATC TGTTCTCAGT TCGGATCGCA GTCTGCAACT CGACTGCGTG AAGCTGGAAT CGCTAGTAAT CGCGGATCAG CAT-GCCGCG

Bacillus_subtilis_(KX450400.1) CG-GAGGAAG GTGGGGATGA CGTCAAATCA TCATGCCCCT TATGACCTGG GCTACACACG TGCTACAATG GACAGAACAA AGGGCAGCGA AACCGCGAGG TTAAGCCAAT CCCACAAATC TGTTCTCAGT TCGGATCGCA GTCTGCAACT CGACTGCGTG AAGCTGGAAT CGCTAGTAAT CGCGGATCAG CAT-GCCGCG

PaKu25_-_Bacillus_cereus CG-GAGGAAG GTGGGGATGA CGTCAAATCA TCATGCCCCT TATGACCTGG GCTACACACG TGCTACAATG GACGGTACAA AGAGCTGCAA GACCGCGAGG TGGAGCTAAT CTCATAAAAC CGTTCTCAGT TCGGATTGTA GGCTGCAACT CGCCTACATG AAGCTGGAAT CGCTAGTAAT CGCGGATCAG CAT-GCCGCG

Bacillus_thuringiensis_(CP0378 CG-GAGGAAG GTGGGGATGA CGTCAAATCA TCATGCCCCT TATGACCTGG GCTACACACG TGCTACAATG GACGGTACAA AGAGCTGCAA GACCGCGAGG TGGAGCTAAT CTCATAAAAC CGTTCTCAGT TCGGATTGTA GGCTGCAACT CGCCTACATG AAGCTGGAAT CGCTAGTAAT CGCGGATCAG CAT-GCCGCG

Bacillus_mobilis_(CP031443.1) CG-GAGGAAG GTGGGGATGA CGTCAAATCA TCATGCCCCT TATGACCTGG GCTACACACG TGCTACAATG GACGGTACAA AGAGCTGCAA GACCGCGAGG TGGAGCTAAT CTCATAAAAC CGTTCTCAGT TCGGATTGTA GGCTGCAACT CGCCTACATG AAGCTGGAAT CGCTAGTAAT CGCGGATCAG CAT-GCCGCG

Bacillus_thuringiensis_(CP0357 CG-GAGGAAG GTGGGGATGA CGTCAAATCA TCATGCCCCT TATGACCTGG GCTACACACG TGCTACAATG GACGGTACAA AGAGCTGCAA GACCGCGAGG TGGAGCTAAT CTCATAAAAC CGTTCTCAGT TCGGATTGTA GGCTGCAACT CGCCTACATG AAGCTGGAAT CGCTAGTAAT CGCGGATCAG CAT-GCCGCG

Bacillus_cereus_(CP030982.1) CG-GAGGAAG GTGGGGATGA CGTCAAATCA TCATGCCCCT TATGACCTGG GCTACACACG TGCTACAATG GACGGTACAA AGAGCTGCAA GACCGCGAGG TGGAGCTAAT CTCATAAAAC CGTTCTCAGT TCGGATTGTA GGCTGCAACT CGCCTACATG AAGCTGGAAT CGCTAGTAAT CGCGGATCAG CAT-GCCGCG

Bacillus_cereus_(MH068823.1) CG-GAGGAAG GTGGGGATGA CGTCAAATCA TCATGCCCCT TATGACCTGG GCTACACACG TGCTACAATG GACGGTACAA AGAGCTGCAA GACCGCGAGG TGGAGCTAAT CTCATAAAAC CGTTCTCAGT TCGGATTGTA GGCTGCAACT CGCCTACATG AAGCTGGAAT CGCTAGTAAT CGCGGATCAG CAT-GCCGCG

Bacillus_thuringiensis_(MG7453 CG-GAGGAAG GTGGGGATGA CGTCAAATCA TCATGCCCCT TATGACCTGG GCTACACACG TGCTACAATG GACGGTACAA AGAGCTGCAA GACCGCGAGG TGGAGCTAAT CTCATAAAAC CGTTCTCAGT TCGGATTGTA GGCTGCAACT CGCCTACATG AAGCTGGAAT CGCTAGTAAT CGCGGATCAG CAT-GCCGCG

Rhizobium_massiliae(Country-_T CGAGAGGAAG GTGGGGATGA CGTCAAGTCC TCATGGCCCT TACGGGCTGG GCTACACACG TGCTACAATG GTGGTGACAG TGGGCAGCGA GACAGCGATG TCGAGCTAAT CTCCAAAAGC CA-TCTCAGT TCGGATTGCA CTCTGCAACT CGAGTGCATG AAGTTGGAAT CGCTAGTAAT CGCAGATCAG CAT-GCTGCG

Stenotrophomonas_maltophilia_( CG-GAGGAAG GTGGGGATGA CGTCAAGTCA TCATGGCCCT TACGGCCAGG GCTACACACG TACTACAATG GTAGGGACAG AGGGCTGCAA GCCGGCGACG GTAAGCCAAT CCCAGAAACC CTATCTCAGT CCGGATTGGA GTCTGCAACT CGACTCCATG AAGTCGGAAT TGCTAGTAAT CGCAGATCAG TATTGCTGCG

Serratia_sp._(Country-_Tunisia ---------- ---------- ---------- ---------- ---------- ---------- ---------- ---------- ---------- ---------- ---------- ---------- ---------- ---------- ---------- ---------- ---------- ---------- ---------- ----------

Staphylococcus_saprophyticus_( CG-GAGGAAG GTGGGGATGA CGTCAAATCA TCATGCCCCT TATGATTTGG GCTACACACG TGCTACAATG GACAATACAA AGGGCAGCTA AACCGCGAGG TCATGCAAAT CCCATAAAGT TGTTCTCAGT TCGGATTGTA GTCTGCAACT CGACTACATG AAGCTGGAAT CGCTAGTAAT CGTAGATCAG CAT-GCTACG

Staphylococcus_saprophyticus_( CG-GAGGAAG GTGGGGATGA CGTCAAATCA TCATGCCCCT TATGATTTGG GCTACACACG TGCTACAATG GACAATACAA AGGGCAGCTA AACCGCGAGG TCATGCAAAT CCCATAAAGT TGTTCTCAGT TCGGATTGTA GTCTGCAACT CGACTACATG AAGCTGGAAT CGCTAGTAAT CGTAGATCAG CAT-GCTACG

Bacillus_subtilis_(Country-Ira CG-GAGGAAG GTGGGGATGA CGTCAAATCA TCATGCCCCT TATGACCTGG GCTACACACG TGCTACAATG GACAGAACAA AGGGCAGCGA AACCGCGAGG TTAAGCCAAT CCCACAAATC TGTTCTCAGT TCGGATCGCA GTCTGCAACT CGACTGCGTG AAGCTGGAAT CGCTAGTAAT CGCGGATCAG CAT-GCCGCG

Bacillus_cereus_(Country-UK)_( CG-GAGGAAG GTGGGGATGA CGTCAAATCA TCATGCCCCT TATGACCTGG GCTACACACG TGCTACAATG GACGGTACAA AGAGCTGCAA GACCGCGAGG TGGAGCTAAT CTCATAAAAC CGTTCTCAGT TCGGATTGTA GGCTGCAACT CGCCTACATG AAGCTGGAAT CGCTAGTAAT CGCGGATCA- ----------

....|....| ....|....| ....|....| ....|....| ....|....| ....|....| ....|....| ....|....| ....|....| ....|....| ....|....| ....|....| ....|....| ....|....| ....|....| ....|....| ....|....| ....|....| ....|....|

1405 1415 1425 1435 1445 1455 1465 1475 1485 1495 1505 1515 1525 1535 1545 1555 1565 1575 1585

PaKu1_-_Serratia_marcescens GTGAATACGT TCCCGGGCCT TGTACACACC GCCCGTCACA CCATGGGAGT GGGTTGCAAA AGAAGTAGGT AGCTTAACCT TCG--GGAGG GCGCTTACCA CTTTGTGATT CATGACTGGG GTGAAGTCGT ---------- ---------- ---------- ---------- ---------- ----------

Serratia_marcescens_(KX911721. GTGAATACGT TCCCGGGCCT TGTACACACC GCCCGTCACA CCATGGGAGT GGGTTGCAAA AGAAGTAGGT AGCTTAACCT TCG--GGAGG GCGCTTACCA CTTTGTGATT CATGACTGGG GTGAAGTCGT A--------- ---------- ---------- ---------- ---------- ----------

Serratia_marcescens_(KT992361. GTGAATACGT TCCCGGGCCT TGTACACACC GCCCGTCACA CCATGGGAGT GGGTTGCAAA AGAAGTAGGT AGCTTAACCT TCG--GGAGG GCGCTTACCA CTTTGTGATT CATGACTGGG GTGAAGTCGT AACAAGGTAA CCGTAGGGGA ACCTGCGGTT GGATCACCT- ---------- ----------

PaKu2_-_Enterobacter_sp. GTGAATACGT TCCCGGGCCT TGTACACACC GCCCGTCACA CCATGGGAGT GGGTTGCAAA AGAAGTAGGT AGCTTAACCT TCG--GGAGG GCGCTTACCA CTTTGTGATT CATGACTGGG GTGAAGTC-- ---------- ---------- ---------- ---------- ---------- ----------

Enterobacter_sp._(MG280962.1) GTGAATACGT TCCCGGGCCT TGTACACACC GCCCGTCACA CCATGGGAGT GGGTTGCAAA AGAAGTAGGT AGCTTAACCT TCG--GGAGG GCGCTTACCA CTTTGTGATT CATGACTGGG GTGAAGTC-- ---------- ---------- ---------- ---------- ---------- ----------

Enterobacter_hormaechei_(CP029 GTGAATACGT TCCCGGGCCT TGTACACACC GCCCGTCACA CCATGGGAGT GGGTTGCAAA AGAAGTAGGT AGCTTAACCT TCG--GGAGG GCGCTTACCA CTTTGTGATT CATGACTGGG GTGAAGTC-- ---------- ---------- ---------- ---------- ---------- ----------

PaKu3_-_Staphylococcus_saproph GTGAATACGT TCCCGGGTCT TGTACACACC GCCCGTCACA CCACGAGAGT TTGTAACACC CGAAGCCGGT GGAGTAACCA TTTATGGAGC TAGCCGTCGA AGGTGGGACA AATGATTGGG GTGAAGTC-- ---------- ---------- ---------- ---------- ---------- ----------

Staphylococcus_saprophyticus_( GTGAATACGT TCCCGGGTCT TGTACACACC GCCCGTCACA CCACGAGAGT TTGTAACACC CGAAGCCGGT GGAGTAACCA TTTATGGAGC TAGCCGTCGA AGGTGGGACA AATGATTGGG GTGAAGTCGT ---------- ---------- ---------- ---------- ---------- ----------

Staphylococcus_saprophyticus_( GTGAATACGT TCCCGGGTCT TGTACACACC GCCCGTCACA CCACGAGAGT TTGTAACACC CGAAGCCGGT GGAGTAACCA TTTATGGAGC TAGCCGTCGA AGGTGGGACA AATGATTGGG GTGAAGTCGT AACAAGGTAG CCGTATCGGA AGGTGCGGCT GGATCACC-- ---------- ----------

PaKu4_-_Enterobacter_cloacae GTGAATACGT TCCCGGGCCT TGTACACACC GCCCGTCACA CCATGGGAGT GGGTTGCAAA AGAAGTAGGT AGCTTAACCT TCG--GGAGG GCGCTTACCA CTTTGTGATT CATGACTGGG GTGAAGTC-- ---------- ---------- ---------- ---------- ---------- ----------

Uncultured_Enterobacter_sp._(M GTGAATACGT TCCCGGGCCT TGTACACACC GCCCGTCACA CCATGGGAGT GGGTTGCAAA AGAAGTAGGT AGCTTAACCT TCG--GGAGG GCGCTTACCA CTTTGTGATT CATGACTGGG GTGAAGTCGT AACAAGGTAA CCGTAGGGGA ACCTGCGGTT GGATCACCTC CTT------- ----------

Enterobacter_cloacae_(KY930709 GTGAATACGT TCCCGGGCCT TGTACACACC GCCCGTCACA CCATGGGAGT GGGTTGCAAA AGAAGTAGGT AGCTTAACCT TCG--GGAGG GCGCTTACCA CTTTGTGATT CATGACTGGG GTGAAGTCGT AAAAAGGTAA CC-------- ---------- ---------- ---------- ----------

PaKu5_-_Enterobacter_cloacae GTGAATACGT TCCCGGGCCT TGTACACACC GCCCGTCACA CCATGGGAGT GGGTTGCAAA AGAAGTAGGT AGCTTAACCT TCG--GGAGG GCGCTTACCA CTTTGTGATT CATGACTGGG GTGAAGTCGT C--------- ---------- ---------- ---------- ---------- ----------

Enterobacter_sp._(KU867838.1) GTGAATACGT TCCCGGGCCT TGTACACACC GCCCGTCACA CCATGGGAGT GGGTTGCAAA AGAAGTAGGT AGCTTAACCT TCG--GGAGG GCGCTTACCA CTTTGTGATT CATGACTGGG GTGAAGTCGT AAC------- ---------- ---------- ---------- ---------- ----------

Enterobacter_cloacae_(KX242265 GTGAATACGT TCCCGGGCCT TGTACACACC GCCCGTCACA CCATGGGAGT GGGTTGCAAA AGAAGTAGGT AGCTTAACCT TCG--GGAGG GCGCTTACCA CTTTGTGATT CATGACTGGG GTGAAGTCGT ACAGGGA--- ---------- ---------- ---------- ---------- ----------

Enterobacter_cloacae_(KP794925 GTGAATACGT TCCCGGGCCT TGTACACACC GCCCGTCACA CCATGGGAGT GGGTTGCAAA AGAAGTAGGT AGCTTAACCT TCG--GGAGG GCGCTTACCA CTTTGTGATT CATGACTGGG GTGAAGTCGT AACAAGGTAA CCGTA----- ---------- ---------- ---------- ----------

PaKu6_-_Staphylococcus_sciuri GTGAATACGT TCCCGGGTCT TGTACACACC GCCCGTCACA CCACGAGAGT TTGTAACACC CGAAGCCGGT GGAGTAACCT TTTA-GGAGC TAGCCGTCGA AGGTGGGACA AATGATTGGG GTGAAGTCGT AA-------- ---------- ---------- ---------- ---------- ----------

Staphylococcus_sciuri_(MG70600 GTGAATACGT TCCCGGGTCT TGTACACACC GCCCGTCACA CCACGAGAGT TTGTAACACC CGAAGCCGGT GGAGTAACCT TTTA-GGAGC TAGCCGTCGA AGGTGGGACA AATGATTGGG GTGAAGTCGT AACAAGGTAG CCGTATCGGA AGGTGCGGCT GGATCACCTC CTTTCTAAGG ----------

Staphylococcus_lentus_(MG98829 GTGAATACGT TCCCGGGTCT TGTACACACC GCCCGTCACA CCACGAGAGT TTGTAACACC CGAAGCCGGT GGAGTAACCT TTTA-GGAGC TAGCCGTCGA AGGTGGGACA AATGATTGGG GTGAAGTCTA CGGG------ ---------- ---------- ---------- ---------- ----------

Staphylococcus_sciuri_(MG51742 GTGAATACGT TCCCGGGTCT TGTACACACC GCCCGTCACA CCACGAGAGT TTGTAACACC CGAAGCCGGT GGAGTAACCT TTTA-GGAGC TAGCCGTCGA AGGTGGGACA AATGATTGGG GTGAAGTCGT AACAAGGTAG CCGTATCGGA AGGTGCGGCT GGATCACCTC CTTTCTAAGG ----------

PaKu8_-_Pseudomonas_aeruginosa GTGAATACGT TCCCGGGCCT TGTACACACC GCCCGTCACA CCATGGGAGT GGGTTGCTCC AGAAGTAGCT AGTCTAACCG CAA--GGGGG ACGGTTACCA CGGAGTGATT CATGACTGGG GTGAAGTCGT ---------- ---------- ---------- ---------- ---------- ----------

Uncultured_bacterium_(FM996243 GTGAATACGT TCCCGGGCCT TGTACACACC GCCCGTCACA CCATGGGAGT GGGTTGCTCC AGAAGTAGCT AGTCTAACCG CAA--GGGGG ACGGTTACCA CGGAGTGATT CATGACTGGG GTGAAGTCGT AACAAGGT-- ---------- ---------- ---------- ---------- ----------

Pseudomonas_aeruginosa_(MK4304 GTGAATACGT TCCCGGGCCT TGTACACACC GCCCGTCACA CCATGGGAGT GGGTTGCTCC AGAAGTAGCT AGTCTAACCG CAA--GGGGG ACGGTTACCA CGGAGTGATT CATGACTGGG GTGAAGTCGT AACAAGGTAA CC-------- ---------- ---------- ---------- ----------

PaKu9_-_Staphylococcus_arletta GTGAATACGT TCCCGGGTCT TGTACACACC GCCCGTCACA CCACGAGAGT TTGTAACACC CGAAGCCGGT GGAGTAACCA TTTATGGAGC TAGCCGTCGA AGGTGGGACA AATGATTGGG GTGAAGTCGT ---------- ---------- ---------- ---------- ---------- ----------

Staphylococcus_arlettae_(KX344 GTGAATACGT TCCCGGGTCT TGTACACACC GCCCGTCACA CCACGAGAGT TTGTAACACC CGAAGCCGGT GGAGTAACCA TTTATGGAGC TAGCCGTCGA AGGTGGGACA AATGATTGGG GTGAAGTCGT AACAGGGTAA CCCGTAAAAC ---------- ---------- ---------- ----------

Staphylococcus_sp._(KU891836.1 GTGAATACGT TCCCGGGTCT TGTACACACC GCCCGTCACA CCACGAGAGT TTGTAACACC CGAAGCCGGT GGAGTAACCA TTTATGGAGC TAGCCGTCGA AGGTGGGACA AATGATTGGG GTGAAGTCGT AACAAGGTAA CCGTA----- ---------- ---------- ---------- ----------

PaKu10_-_Aeromonas_caviae GTGAATACGT TCCCGGGCCT TGTACACACC GCCCGTCACA CCATGGGAGT GGGTTGCACC AGAAGTAGAT AGCTTAACCT TCG--GGAGG GCGTTTACCA CGGTGTGATT CATGACTGGG GTGAAGTCGT ---------- ---------- ---------- ---------- ---------- ----------

Uncultured_bacterium_(KX242397 GTGAATACGT TCCCGGGCCT TGTACACACC GCCCGTCACA CCATGGGAGT GGGTTGCACC AGAAGTAGAT AGCTTAACCT TCG--GGAGG GCGTTTACCA CGGTGTGATT CATGACTGGG GTGAAGTCGT AACAAGGTAA CC-------- ---------- ---------- ---------- ----------

Aeromonas_caviae_(HQ407268.1) GTGAATACGT TCCCGGGCCT TGTACACACC GCCCGTCACA CCATGGGAGT GGGTTGCACC AGAAGTAGAT AGCTTAACCT TCG--GGAGG GCGTTTACCA CGGTGTGATT CATGACTGGG GTGAAGTCGT AACAAGGTAA CCCTAGGGGA ACCTGGGGCT G--------- ---------- ----------

Aeromonas_aquariorum_(EU085557 GTGAATACGT TCCCGGGCCT TGTACACACC GCCCGTCACA CCATGGGAGT GGGTTGCACC AGAAGTAGAT AGCTTAACCT TCG--GGAGG GCGTTTACCA CGGTGTGATT CATGACTGGG GTGAAGTCGT AACAAGGTAA ---------- ---------- ---------- ---------- ----------

PaKu11_-_Aeromonas_caviae GTGAATACGT TCCCGGGCCT TGTACACACC GCCCGTCACA CCATGGGAGT GGGTTGCACC AGAAGTAGAT AGCTTAACCT TCG--GGAGG GCGTTTACCA CGGTGTGATT CATGACTGGG GTGAAGTC-- ---------- ---------- ---------- ---------- ---------- ----------

Aeromonas_caviae_(KY087979.1) GTGAATACGT TCCCGGGCCT TGTACACACC GCCCGTCACA CCATGGGAGT GGGTTGCACC AGAAGTAGAT AGCTTAACCT TCG--GGAGG GCGTTTACCA CGGTGTGATT CATGACTGGG GTGAAGTCGT AACAAGGTAA CCC------- ---------- ---------- ---------- ----------

Uncultured_Aeromonas_sp._(KU94 GTGAATACGT TCCCGGGCCT TGTACACACC GCCCGTCACA CCATGGGAGT GGGTTGCACC AGAAGTAGAT AGCTTAACCT TCG--GGAGG GCGTTTACCA CGGTGTGATT CATGACTGGG GTGAAGTC-- ---------- ---------- ---------- ---------- ---------- ----------

PaKu12_-_Staphylococcus_warner GTGAATACGT TCCCGGGTCT TGTACACACC GCCCGTCACA CCACGAGAGT TTGTAACACC CGAAGCCGGT GGAGTAACCA TTTATGGAGC TAGCCGTCGA AGGTGGGACA AATGATTGGG GTGAAGTCGT ---------- ---------- ---------- ---------- ---------- ----------

Staphylococcus_warneri_(MH0149 GTGAATACGT TCCCGGGTCT TGTACACACC GCCCGTCACA CCACGAGAGT TTGTAACACC CGAAGCCGGT GGAGTAACCA TTTATGAAGC TAGCCGTCGA AGGTGGGACA AATGATTGGG GTGAAGTCGT AACAAGGTAA CCGT------ ---------- ---------- ---------- ----------

Staphylococcus_warneri_(KF3063 GTGAATACGT TCCCGGGTCT TGTACACACC GCCCGTCACA CCACGAGAGT TTGTAACACC CGAAGCCGGT GGAGTAACCA TTTATGGAGC TAGCCGTCGA AGGTGGGACA AATGATTGGG GTGAAGTCGT AACAAGGTAA CC-------- ---------- ---------- ---------- ----------

PaKu13_-_Bacillus_megaterium GTGAATACGT TCCCGGGCCT TGTACACACC GCCCGTCACA CCACGAGAGT TTGTAACACC CGAAGTCGGT GGAGTAACCG TAA--GGAGC TAGCCGCCTA AGGTGGGACA GATGATTGGG GTGAAGTCGT ---------- ---------- ---------- ---------- ---------- ----------

Bacillus_sp._(MK691444.1) GTGAATACGT TCCCGGGCCT TGTACACACC GCCCGTCACA CCACGAGAGT TTGTAACACC CGAAGTCGGT GGAGTAACCG TAA--GGAGC TAGCCGCCTA AGGTGGGACA GATGATTGGG GTGAAGTCGT AACAAGGTAA CCGTA----- ---------- ---------- ---------- ----------

Bacillus_sp._(LC373523.1) GTGAATACGT TCCCGGGCCT TGTACACACC GCCCGTCACA CCACGAGAGT TTGTAACACC CGAAGTCGGT GGAGTAACCG TAA--GGAGC TAGCCGCCTA AGGTGGGACA GATGATTGGG GTGAAGTCGT AACAAGGTAA CC-------- ---------- ---------- ---------- ----------

Bacillus_aryabhattai_(MK474942 GTGAATACGT TCCCGGGCCT TGTACACACC GCCCGTCACA CCACGAGAGT TTGTAACACC CGAAGTCGGT GGAGTAACCG TAA--GGAGC TAGCCGCCTA AGGTGGGACA GATGATTGGG GTGAAGTCGT AACAAGG--- ---------- ---------- ---------- ---------- ----------

Bacillus_aryabhattai_(MK474941 GTGAATACGT TCCCGGGCCT TGTACACACC GCCCGTCACA CCACGAGAGT TTGTAACACC CGAAGTCGGT GGAGTAACCG TAA--GGAGC TAGCCGCCTA AGGTGGGACA GATGATTGGG GTGAAGTCGT AACAAGGTA- ---------- ---------- ---------- ---------- ----------

Bacillus_megaterium_(KX298860. GTGAATACGT TCCCGGGCCT TGTACACACC GCCCGTCACA CCACGAGAGT TTGTAACACC CGAAGTCGGT GGAGTAACCG TAA--GGAGC TAGCCGCCTA AGGTGGGACA GATGATTGGG GTGAAGTCGT AACAGGGTAA CCCGTAA--- ---------- ---------- ---------- ----------

PaKu14_-_Pseudomonas_stutzeri GTGAATACGT TCCCGGGCCT TGTACACACC GCCCGTCACA CCATGGGAGT GGGTTGCTCC AGAAGTAGCT AGTCTAACCT TCG--GGGGG ACGGTTACCA CGGAGTGATT CATGACTGGG GTGAA----- ---------- ---------- ---------- ---------- ---------- ----------

Pseudomonas_stutzeri_(MG892782 GTGAATACGT TCCCGGGCCT TGTACACACC GCCCGTCACA CCATGGGAGT GGGTTGCTCC AGAAGTAGCT AGTCTAACCT TCG--GGGGG ACGGTTACCA CGGAGTGATT CATGACTGGG GTGAAGTCGT ACAGGG---- ---------- ---------- ---------- ---------- ----------

Pseudomonas_stutzeri_(MG595371 GTGAATACGT TCCCGGGCCT TGTACACACC GCCCGTCACA CCATGGGAGT GGGTTGCTCC AGAAGTAGCT AGTCTAACCT TCG--GGGGG ACGGTTACCA CGGAGTGATT CATGACTGGG GTGAAGTCGT ACAGGG---- ---------- ---------- ---------- ---------- ----------

PaKu15_-_Rhizobium_sp. GTGAATACGT TCCCGGGCCT TGTACACACC GCCCGTCACA CCATGGGAGT TGGTTTTACC CGAAGGTAGT GCGCTAACCG CAA--GGAGG CAGCTAACCA CGGTAGGGTC AGCGACTGGG GTGAAGTCG- ---------- ---------- ---------- ---------- ---------- ----------

Rhizobium_pusense_(CP039895.1) GTGAATACGT TCCCGGGCCT TGTACACACC GCCCGTCACA CCATGGGAGT TGGTTTTACC CGAAGGTAGT GCGCTAACCG CAA--GGAGG CAGCTAACCA CGGTAGGGTC AGCGACTGGG GTGAAGTCG- ---------- ---------- ---------- ---------- ---------- ----------

Rhizobium_sp._(KY971009.1) GTGAATACGT TCCCGGGCCT TGTACACACC GCCCGTCACA CCATGGGAGT TGGTTTTACC CGAAGGTAGT GCGCTAACCG CAA--GGAGG CAGCTAACCA CGGTAGGGTC AGCGACTGGG GTGAAGTCGT AACAAGGTAG CCGTAGGGGA ACCTGCGGCT GGATCACCTC CTTAAGCTTG GATCCCGGGA

PaKu7_-_Stenotrophomonas_malto GTGAATACGT TCCCGGGCCT TGTACACACC GCCCGTCACA CCATGGGAGT TTGTTGCACC AGAAGCAGGT AGCTTAACCT TCG--GGAGG GCGCTTGCCA CGGTGTGGCC GATGACTGGG GTGAAGTCGT AACAAGG--- ---------- ---------- ---------- ---------- ----------

Stenotrophomonas_maltophilia_( GTGAATACGT TCCCGGGCCT TGTACACACC GCCCGTCACA CCATGGGAGT TTGTTGCACC AGAAGCAGGT AGCTTAACCT TCG--GGAGG GCGCTTGCCA CGGTGTGGCC GATGACTGGG GTGAAGTCGT AACAAGGTAA CCGT------ ---------- ---------- ---------- ----------

Stenotrophomonas_maltophilia_( GTGAATACGT TCCCGGGCCT TGTACACACC GCCCGTCACA CCATGGGAGT TTGTTGCACC AGAAGCAGGT AGCTTAACCT TCG--GGAGG GCGCTTGCCA CGGTGTGGCC GATGACTGGG GTGAAGTCGT AACAAGGTAG CCGTATCGGA AGGTGCG--- ---------- ---------- ----------

PaKu16_-_Stenotrophomonas_malt GTGAATACGT TCCCGGGCCT TGTACACACC GCCCGTCACA CCATGGGAGT TTGTTGCACC AGAAGCAGGT AGCTTAACCT TCG--GGAGG GCGCTTGCCA CGGTGTGGCC GATGACTGGG GTGA------ ---------- ---------- ---------- ---------- ---------- ----------

Stenotrophomonas_maltophilia_( GTGAATACGT TCCCGGGCCT TGTACACACC GCCCGTCACA CCATGGGAGT TTGTTGCACC AGAAGCAGGT AGCTTAACCT TCG--GGAGG GCGCTTGCCA CGGTGTGGCC GATGACTGGG GTGAAGTCGT ACAGGGAAAC ---------- ---------- ---------- ---------- ----------

Stenotrophomonas_maltophilia_( GTGAATACGT TCCCGGGCCT TGTACACACC GCCCGTCACA CCATGGGAGT TTGTTGCACC AGAAGCAGGT AGCTTAACCT TCG--GGAGG GCGCTTGCCA CGGTGTGGCC GATGACTGGG GTGAAGTCGT AACAAGGTAG CCGTATCGGA AGGTGCGG-- ---------- ---------- ----------

PaKu17_-_Stenotrophomonas_malt GTGAATACGT TCCCGGGCCT TGTACACACC GCCCGTCACA CCATGGGAGT TTGTTGCACC AGAAGCAGGT AGCTTAACCT TCG--GGAGG GCGCTTGCCA CGGTGTGGCC GATGACTGGG GTGAAGTC-- ---------- ---------- ---------- ---------- ---------- ----------

PaKu26_-_Stenotrophomonas_malt GTGAATACGT TCCCGGGCCT TGTACACACC GCCCGTCACA CCATGGGAGT TTGTTGCACC AGAAGCAGGT AGCTTAACCT TCG--GGAGG GCGCTTGCCA CGGTGTGGCC GATGACTGGG GTGA------ ---------- ---------- ---------- ---------- ---------- ----------

Stenotrophomonas_maltophilia_( GTGAATACGT TCCCGGGCCT TGTACACACC GCCCGTCACA CCATGGGAGT TTGTTGCACC AGAAGCAGGT AGCTTAACCT TCG--GGAGG GCGCTTGCCA CGGTGTGGCC GATGACTGGG GTGAAGTCGT AACAAGGTAG CCGTATCGGA AGGTGCGGCT GGATCACCTC CTT------- ----------

PaKu18_-_Stenotrophomonas_pana GTGAATACGT TCCCGGGCCT TGTACACACC GCCCGTCACA CCATGGGAGT TTGTTGCACC AGAAGCAGGT AGCTTAACCT TCG--GGAGG GCGCTTGCCA CGGTGTGGCC GATGACTGGG GTGAAG---- ---------- ---------- ---------- ---------- ---------- ----------

Stenotrophomonas_panacihumi_(M GTGAATACGT TCCCGGGCCT TGTACACACC GCCCGTCACA CCATGGGAGT TTGTTGCACC AGAAGCAGGT AGCTTAACCT TCG--GGAGG GCGCTTGCCA CGGTGTGGCC GATGACTGGG GTGAAGTCGT AGAAGG---- ---------- ---------- ---------- ---------- ----------

Stenotrophomonas_panacihumi_(K GTGAATACGT TCCCGGGCCT TGTACACACC GCCCGTCACA CCATGGGAGT TTGTTGCACC AGAAGCAGGT AGCTTAACCT TCG--GGAGG GCGCTTGCCA CGGTGTGGCC GATGACTGGG GTGAAGTCGT AACAAGGTAG CCGTATCGGA AGGTGCGGGT GGATCACTCC CCTT------ ----------

PaKu19_-_Bacillus_licheniformi GTGAATACGT TCCCGGGCCT TGTACACACC GCCCGTCACA CCACGAGAGT TTGTAACACC CGAAGTCGGT GAGGTAACCT TTT--GGAGC CAGCCGCCGA AGGTGGGACA GATGATTGGG GTGAA----- ---------- ---------- ---------- ---------- ---------- ----------

Bacillus_licheniformis_(KJ5722 GTGAATACGT TCCCGGGCCT TGTACACACC GCCCGTCACA CCACGAGAGT TTGTAACACC CGAAGTCGGT GAGGTAACCT TTT--GGAGC CAGCCGCCGA AGGTGGGACA GATGATTGGG GTGAA----- ---------- ---------- ---------- ---------- ---------- ----------

Bacillus_licheniformis_(KT1536 GTGAATACGT TCCCGGGCCT TGTACACACC GCCCGTCACA CCACGAGAGT TTGTAACACC CGAAGTCGGT GAGGTAACCT TTT--GGAGC CAGCCGCCGA AGGTGGGACA GATGATTGGG GTGAATCTAG G--------- ---------- ---------- ---------- ---------- ----------

PaKu20_-__Pantoea_dispersa GTGAATACGT TCCCGGGCCT TGTACACACC GCCCGTCACA CCATGGGAGT GGGTTGCAAA AGAAGTAGGT AGCTTAACCT TCG--GGAGG GCGCTTACCA CTTTGTGATT CATGACTGGG GTGA------ ---------- ---------- ---------- ---------- ---------- ----------

Pantoea_dispersa_(KY292463.1) GTGAATACGT TCCCGGGCCT TGTACACACC GCCCGTCACA CCATGGGAGT GGGTTGCAAA AGAAGTAGGT AGCTTAACCT TCG--GGAGG GCGCTTACCA CTTTGTGATT CATGACTGGG GTGAAGTCGT AACAAG---- ---------- ---------- ---------- ---------- ----------

Pantoea_dispersa_(KY882077.1) GTGAATACGT TCCCGGGCCT TGTACACACC GCCCGTCACA CCATGGGAGT GGGTTGCAAA AGAAGTAGGT AGCTTAACCT TCG--GGAGG GCGCTTACCA CTTTGTGATT CATGACTGGG GTGA------ ---------- ---------- ---------- ---------- ---------- ----------

PaKu21_-_Bacillus_sonorensis GTGAATACGT TCCCGGGCCT TGTACACACC GCCCGTCACA CCACGAGAGT TTGTAACACC CGAAGTCGGT GAGGTAACCT TTT--GGAGC CAGCCGCCGA AGGTGGGACA GATGATTGGG GTGAAGTC-- ---------- ---------- ---------- ---------- ---------- ----------

Bacillus_sonorensis_(KU551167. GTGAATACGT TCCCGGGCCT TGTACACACC GCCCGTCACA CCACGAGAGT TTGTAACACC CGAAGTCGGT GAGGTAACCT TTT--GGAGC CAGCCGCCGA AGGTGGGACA GATGATTGGG GTGAAGTCGT AACAAGGTAA CC-------- ---------- ---------- ---------- ----------

Bacillus_sonorensis_(FN397516. GTGAATACGT TCCCGGGCCT TGTACACACC GCCCGTCACA CCACGAGAGT TTGTAACACC CGAAGTCGGT GAGGTAACCT TTT--GGAGC CAGCCGCCGA AGGTGGGACA GATGATTGGG GTGAAGTCGT AACAA----- ---------- ---------- ---------- ---------- ----------

Bacillus_sonorensis_(KU551137. GTGAATACGT TCCCGGGCCT TGTACACACC GCCCGTCACA CCACGAGAGT TTGTAACACC CGAAGTCGGT GAGGTAACCT TTT--GGAGC CAGCCGCCGA AGGTGGGACA GATGATTGGG GTGAAGTCGT AACAAGGTAA CC-------- ---------- ---------- ---------- ----------

PaKu24_-_Bacillus_sonorensis GTGAATACGT TCCCGGGCCT TGTACACACC GCCCGTCACA CCACGAGAGT TTGTAACACC CGAAGTCGGT GAGGTAACCT TTT--GGAGC CAGCCGCCGA AGGTGGGACA GATGATTGGG GTGAA----- ---------- ---------- ---------- ---------- ---------- ----------

PaKu22_-_Bacillus_subtilis GTGAATACGT TCCCGGGCCT TGTACACACC GCCCGTCACA CCACGAGAGT TTGTAACACC CGAAGTCGGT GAGGTAACCT TTTA-GGAGC CAGCCGCCGA AGGTGGGACA GATGATTGGG GTGAAGTC-- ---------- ---------- ---------- ---------- ---------- ----------

Bacillus_tequilensis_(MK880583 GTGAATACGT TCCCGGGCCT TGTACACACC GCCCGTCACA CCACGAGAGT TTGTAACACC CGAAGTCGGT GAGGTAACCT TTTA-GGAGC CAGCCGCCGA AGGTGGGACA GATGATTGGG GTGAAGTCTA CAAA------ ---------- ---------- ---------- ---------- ----------

Bacillus_subtilis_(MK765023.1) GTGAATACGT TCCCGGGCCT TGTACACACC GCCCGTCACA CCACGAGAGT TTGTAACACC CGAAGTCGGT GAGGTAACCT TTTA-GGAGC CAGCCGCCGA AGGTGGGACA GATGATTGGG GTGAAGTC-- ---------- ---------- ---------- ---------- ---------- ----------

PaKu23_-_Bacillus_subtilis GTGAATACGT TCCCGGGCCT TGTACACACC GCCCGTCACA CCACGAGAGT TTGTAACACC CGAAGTCGGT GAGGTAACCT TTTA-GGAGC CAGCCGCCGA AGGTGGGACA GATGATTGGG GTGAAGTCG- ---------- ---------- ---------- ---------- ---------- ----------

Bacillus_subtilis_(MK511833.1) GTGAATACGT TCCCGGGCCT TGTACACACC GCCCGTCACA CCACGAGAGT TTGTAACACC CGAAGTCGGT GAGGTAACCT TTTA-GGAGC CAGCCGCCGA AGGTGGGACA GATGATTGGG GTGAAGTCGT AAAGGGGAAA CCC------- ---------- ---------- ---------- ----------

Bacillus_subtilis_(KX450400.1) GTGAATACGT TCCCGGGCCT TGTACACACC GCCCGTCACA CCACGAGAGT TTGTAACACC CGAAGTCGGT GAGGTAACCT TTTA-GGAGC CAGCCGCCGA AGGTGGGACA GATGATTGGG GTGAAGTCGT AAAGGGGAAA CCCCAAA--- ---------- ---------- ---------- ----------

PaKu25_-_Bacillus_cereus GTGAATACGT TCCCGGGCCT TGTACACACC GCCCGTCACA CCACGAGAGT TTGTAACACC CGAAGTCGGT GGGGTAACCT TTTT-GGAGC CAGCCGCCTA AGGTGGGACA GATGATTGGG GTGAAGTCG- ---------- ---------- ---------- ---------- ---------- ----------

Bacillus_thuringiensis_(CP0378 GTGAATACGT TCCCGGGCCT TGTACACACC GCCCGTCACA CCACGAGAGT TTGTAACACC CGAAGTCGGT GGGGTAACCT TTTT-GGAGC CAGCCGCCTA AGGTGGGACA GATGATTGGG GTGAA----- ---------- ---------- ---------- ---------- ---------- ----------

Bacillus_mobilis_(CP031443.1) GTGAATACGT TCCCGGGCCT TGTACACACC GCCCGTCACA CCACGAGAGT TTGTAACACC CGAAGTCGGT GGGGTAACCT TTTT-GGAGC CAGCCGCCTA AGGTGGGACA GATGATTGGG GTGAA----- ---------- ---------- ---------- ---------- ---------- ----------

Bacillus_thuringiensis_(CP0357 GTGAATACGT TCCCGGGCCT TGTACACACC GCCCGTCACA CCACGAGAGT TTGTAACACC CGAAGTCGGT GGGGTAACCT TTTT-GGAGC CAGCCGCCTA AGGTGGGACA GATGATTGGG GTGAA----- ---------- ---------- ---------- ---------- ---------- ----------

Bacillus_cereus_(CP030982.1) GTGAATACGT TCCCGGGCCT TGTACACACC GCCCGTCACA CCACGAGAGT TTGTAACACC CGAAGTCGGT GGGGTAACCT TTTT-GGAGC CAGCCGCCTA AGGTGGGACA GATGATTGGG GTGAA----- ---------- ---------- ---------- ---------- ---------- ----------

Bacillus_cereus_(MH068823.1) GTGAATACGT TCCCGGGCCT TGTACACACC GCCCGTCACA CCACGAGAGT TTGTAACACC CGAAGTCGGT GGGGTAACCT TTTT-GGAGC CAGCCGCCTA AGGTGGGACA GATGATTGGG GTGAAGTCGT AACAAGGTAG CCGTATCGGA AGGTGCGGCT GGATCACCT- ---------- ----------

Bacillus_thuringiensis_(MG7453 GTGAATACGT TCCCGGGCCT TGTACACACC GCCCGTCACA CCACGAGAGT TTGTAACACC CGAAGTCGGT GGGGTAACCT TTTT-GGAGC CAGCCGCCTA AGGTGGGACA GATGATTGGG GTGAA----- ---------- ---------- ---------- ---------- ---------- ----------

Rhizobium_massiliae(Country-_T GTGAATACGT TCCCGGGCCT TGTACACACC GCCCGTCACA CCATGGGAGT TGGTTTTACC CGAAGACA-- ---------- ---------- ---------- ---------- ---------- ---------- ---------- ---------- ---------- ---------- ---------- ----------

Stenotrophomonas_maltophilia_( GTGAATACGT TCCCGGGCCT TG-------- ---------- ---------- ---------- ---------- ---------- ---------- ---------- ---------- ---------- ---------- ---------- ---------- ---------- ---------- ---------- ----------

Serratia_sp._(Country-_Tunisia ---------- ---------- ---------- ---------- ---------- ---------- ---------- ---------- ---------- ---------- ---------- ---------- ---------- ---------- ---------- ---------- ---------- ---------- ----------

Staphylococcus_saprophyticus_( GTGAATACGT TCCCGGGTCT TGTACACACC GCCCGTCACA CCACGAGAGT TTGTAACACC CGAAGCCGGT GGAGTAACCA TTTATGGAGC TAGCCGTCGA AGGTG----- ---------- ---------- ---------- ---------- ---------- ---------- ---------- ----------

Staphylococcus_saprophyticus_( GTGAATACGT TCCCGGGTCT TGTACACACC GCCCGTCACA CCACGAGAGT TTGTAACACC CGAAGCCGGT GGAGTAACCA TTTATGGAGC TAGCCGTCGA AGGTGG-ACA ---------- ---------- ---------- ---------- ---------- ---------- ---------- ----------

Bacillus_subtilis_(Country-Ira GTGAATACGT TCCCGGGCCT TGTACACACC GCCCGTCACA CCACGAGAGT TTGTAACACC CGAAGTCGGT GAGGTAACCT TTTA-GGAGC CAGCCGCCGA A--------- ---------- ---------- ---------- ---------- ---------- ---------- ---------- ----------

Bacillus_cereus_(Country-UK)_( ---------- ---------- ---------- ---------- ---------- ---------- ---------- ---------- ---------- ---------- ---------- ---------- ---------- ---------- ---------- ---------- ---------- ---------- ----------
